# MCL-CAw: a refinement of MCL for detecting yeast complexes from weighted PPI networks by incorporating core-attachment structure

**DOI:** 10.1186/1471-2105-11-504

**Published:** 2010-10-12

**Authors:** Sriganesh Srihari, Kang Ning, Hon Wai Leong

**Affiliations:** 1 Department of Computer Science, National University of Singapore, 117590, Singapore; 2 Department of Pathology, University of Michigan, Ann Arbor, MI 48109, USA; 3 Qingdao Institute of Bioenergy and Bioprocess Technology, Qingdao 266101, China

## Abstract

**Background:**

The reconstruction of protein complexes from the physical interactome of organisms serves as a building block towards understanding the higher level organization of the cell. Over the past few years, several independent high-throughput experiments have helped to catalogue enormous amount of physical protein interaction data from organisms such as yeast. However, these individual datasets show lack of correlation with each other and also contain substantial number of false positives (noise). Over these years, several affinity scoring schemes have also been devised to improve the qualities of these datasets. Therefore, the challenge now is to detect meaningful as well as novel complexes from protein interaction (PPI) networks derived by combining datasets from multiple sources and by making use of these affinity scoring schemes. In the attempt towards tackling this challenge, the Markov Clustering algorithm (MCL) has proved to be a popular and reasonably successful method, mainly due to its scalability, robustness, and ability to work on scored (weighted) networks. However, MCL produces many noisy clusters, which either do not match known complexes or have additional proteins that reduce the accuracies of correctly predicted complexes.

**Results:**

Inspired by recent experimental observations by Gavin and colleagues on the modularity structure in yeast complexes and the distinctive properties of "core" and "attachment" proteins, we develop a core-attachment based refinement method coupled to MCL for reconstruction of yeast complexes from scored (weighted) PPI networks. We combine physical interactions from two recent "pull-down" experiments to generate an unscored PPI network. We then score this network using available affinity scoring schemes to generate multiple scored PPI networks. The evaluation of our method (called MCL-CAw) on these networks shows that: (i) MCL-CAw derives larger number of yeast complexes and with better accuracies than MCL, particularly in the presence of natural noise; (ii) Affinity scoring can effectively reduce the impact of noise on MCL-CAw and thereby improve the quality (precision and recall) of its predicted complexes; (iii) MCL-CAw responds well to most available scoring schemes. We discuss several instances where MCL-CAw was successful in deriving meaningful complexes, and where it missed a few proteins or whole complexes due to affinity scoring of the networks. We compare MCL-CAw with several recent complex detection algorithms on unscored and scored networks, and assess the relative performance of the algorithms on these networks. Further, we study the impact of augmenting physical datasets with computationally inferred interactions for complex detection. Finally, we analyse the essentiality of proteins within predicted complexes to understand a possible correlation between protein essentiality and their ability to form complexes.

**Conclusions:**

We demonstrate that core-attachment based refinement in MCL-CAw improves the predictions of MCL on yeast PPI networks. We show that affinity scoring improves the performance of MCL-CAw.

## Background

Most biological processes are carried out by proteins that physically interact to form stoichiometrically stable complexes. Even in the relatively simple model organism *Saccharomyces cerevisiae *(budding yeast), these complexes are comprised of many subunits that work in a coherent fashion. These complexes interact with individual proteins or other complexes to form functional modules and pathways that drive the cellular machinery. Therefore, a faithful reconstruction of the entire set of complexes from the physical interactions between proteins is essential to not only understand complex formations, but also the higher level organization of the cell.

These physical interactions between proteins have been most extensively catalogued for yeast using high-throughput methods like yeast two-hybrid [1,2] and direct purification of complexes using affinity tags followed by mass spectrometry (MS) analyses [3]. In 2002, the direct purification strategy or "pull-down" was first applied to yeast in two independent studies by Gavin *et al. *[4] and Ho *et al. *[5]. More recently (2006), two separate groups, Gavin *et al. *[6] and Krogan *et al. *[7], employed tandem affinity purification (TAP) followed by MS analyses to produce enormous amount of new data, allowing a more complete mapping of the yeast interactome. Although these individual datasets are of high quality, they show surprising lack of correlation with each other [8,9], and some bias towards high abundance proteins [10] and against proteins from certain cellular compartments (like cell wall and plasma membrane) [11]. Also, each dataset still contains a substantial number of false positives (noise) that can compromise the utility of these datasets for more focused studies like complex reconstruction [11]. In order to reduce the impact of such discrepancies, a number of data integration and affinity scoring schemes have been devised [6,7,11-17]. These affinity scores encode the reliabilities (confidence) of physical interactions between pairs of proteins. Therefore, the challenge now is to detect meaningful as well as novel complexes from protein interaction (PPI) networks derived by combining multiple high-throughput datasets and by making use of these affinity scoring schemes.

The interaction data produced from the high-throughput TAP/MS experiments comprise of tagged "bait" proteins and the associated "prey" proteins that co-purify with the baits. Gavin *et al. *[6] considered direct bait-prey as well as indirect prey-prey relationships (a combination of spoke and matrix models), followed by a socio-affinity scoring system to encode the affinities between the protein pairs. The socio-affinity score quantizes the log-ratio of the number of times two proteins are observed together relative to what would be expected from their frequency in the dataset. Subsequently, Gavin *et al. *used an iterative clustering approach to derive complexes. Each complex was then partitioned into groups of proteins called "core", "attachment" or "module" (depicted in Additional files 1, Figure S1). On the other hand, Krogan *et al. *[7] used machine learning techniques (Bayesian networks and C4.5-based decision trees) to define confidence scores for interactions derived from direct bait-prey observations (the spoke model). Subsequently, Krogan *et al. *defined a high-confidence 'Core' dataset of interactions, and used the Markov Clustering algorithm (MCL) [18,19] to derive complexes. Hart *et al. *[12] generated a Probabilistic Integrated Co-complex (PICO) network by integrating matrix modeled relationships of the Gavin *et al*., Krogan *et al. *and Ho *et al. *datasets using a measure similar to socio-affinity scores, and then used a MCL procedure to derive complexes from this network. Collins *et al. *[11] developed a Purification Enrichment (PE) scoring system to generate the 'Consolidated network' from the matrix modeled relationships of the Gavin *et al*., and Krogan *et al. *datasets. Collins *et al. *used a Bayes classifier to generate the PE scores in the Consolidated network by incorporating diverse evidence from hand-curated co-complexed protein pairs, Gene Ontology (GO) annotations, mRNA expression patterns, and cellular co-localization and co-expression profiles. This new network was shown to be of high quality - comparable to that of PPIs derived from small-scale experiments stored at the Munich Information Center for Protein Sequences (MIPS). Zhang *et al. *[13] used Dice coefficient (DC) to assign affinities to protein pairs, and evaluated their affinity measure against socio-affinity and PE measures. They concluded that DC and PE offered the best representation for protein affinity, and subsequently used them for complex prediction. Pu *et al. *[20] used MCL combined with cluster overlaps on the Consolidated network to reveal interesting insights into complex organization. Wang *et al. *[21] proposed HACO, a hierarchical clustering with overlap algorithm, to reconstruct complexes and used them to build the 'ComplexNet', an interaction network of proteins and complexes, in order to study the higher-level organization of complexes. Chua *et al. *[14] and Liu *et al. *[15] developed network topology-based scoring schemes called Functional Similarity Weight (FS Weight) and Iterative-Czekanowski-Dice (Iterative-CD), respectively, to assign reliability scores to the interactions in networks. Subsequently, Liu *et al. *[16] used a maximal clique merging strategy (called CMC) to derive complexes from networks scored using these two systems. Friedel *et al. *[17] developed a bootstrapped scoring system to score TAP/MS interactions from Gavin *et al. *and Krogan *et al*., and subsequently derived complexes using a variant of MCL. Friedel *et al. *[22] also developed a minimum spanning tree-based method to reconstruct the topology of complexes from co-purified proteins in TAP/MS assays. Voevodski *et al. *[23] used PageRank, a random walk-based method employed in context-sensitive web search, to define the affinities between proteins within PPI networks. Subsequently, Voevodski *et al. *used it to predict co-complexed proteins within the network. Approaches like CORE [24] and COACH [25] adopted local dense neighborhood search to derive cores and attachments from unscored networks. Mitrofanova *et al. *[26] measured the connectivity between proteins in unweighted PPI networks by edge-disjoint paths instead of edges to overcome noise, and modeled these paths as a network flow and represented it in Gomory-Hu trees. They subsequently isolated groups of nodes in the trees that shared edge-disjoint paths in order to identify complexes. Very recently, Ozawa *et al. *[27] used domain-domain interactions to validate and refine the complexes predicted by MCL.

In this study, we develop an algorithm to derive yeast complexes from weighted (affinity-scored) PPI networks. Inspired by the experimental findings by Gavin *et al. *[6] on the modularity structure in yeast complexes, and the distinctive properties of "core" and "attachment" proteins, we develop a novel core-attachment based refinement method coupled to MCL for reconstruction of yeast complexes. We had proposed the idea of core-attachment based refinement in a preliminary work [28] and called it MCL-CA.

However, MCL-CA worked only on unscored networks. Here, we devise an improved algorithm (called MCL-CAw) and provide a natural extension to work on scored (weighted) PPI networks. Even though most eukaryotic complexes are hypothesized to display such core-attachment modularity, here we design our algorithm specific to yeast complexes because of lack of sufficient evidence, high-throughput datasets and reference complexes from other organisms. We combine TAP/MS physical datasets from Gavin *et al. *[6] and Krogan *et al. *[7] to generate an unscored PPI network (Table 1). We then score this network using two topology-based affinity scoring schemes, FS Weight [14] and Iterative-CD [15], to generate scored PPI networks. We gather two additional readily-available scored PPI networks from Collins *et al. *[11] and Friedel *et al. *[17]. The evaluation of MCL-CAw on these networks demonstrates that: (a) MCL-CAw is able to derive higher number of yeast complexes and with better accuracies than MCL; (b) Affinity scoring effectively reduces the impact of noise on MCL-CAw and thereby improves the quality (precision and recall) of its predicted complexes; (c) MCL-CAw responds well to most available affinity scoring schemes for PPI networks. We compare MCL-CAw with several recent complex detection algorithms on both unscored and scored PPI networks. Finally, we perform in-depth analysis of the predicted complexes from MCL-CAw.

**Table 1:** Properties of the PPI networks used for the evaluation of MCL-CAw

PPI Network	# Proteins	# Interactions	Avg node degree
Gavin	1430	7592	10.62
Krogan 'Core'	2708	7123	5.26

Gavin+Krogan	2964	13507	9.12
ICD(Gavin+Krogan)	1628	8707	10.69
FSW(Gavin+Krogan)	1628	8688	10.67
Consolidated_3.19_	1622	9704	11.96
Bootstrap_0.094_	2719	10290	7.56

Inferred	954	11892	24.93
Gavin+Krogan+Inferred	3418	25352	14.83
ICD(Gavin+Krogan+Inferred)	2034	12009	11.81
FSW(Gavin+Krogan+Inferred)	1892	11705	12.37

The Gavin+Krogan network was generated by combining the Gavin and Krogan-Core datasets, obtained from BioGrid [32]. The ICD(Gavin+Krogan) and FSW(Gavin+Krogan) networks were generated by scoring the Gavin+Krogan network using the Iterative-CD*^k ^*and FS Weight*^k ^*schemes [14,15] (with *k *= 2 iterations). The Consolidated_3.19 _network refers to the high-confidence subset (PE cut- off: 3.19) of the Consolidated network derived by Collins *et al. *[11]. The Bootstrap_0.094 _network refers to the high-confidence subset (BT cut-off: 0.094) of the Bootstrap network derived by Friedel *et al. *[17]. The Inferred network comprised of computationally inferred interactions from the Predictome database [40]. The Gavin+Krogan+Inferred network was generated by augmenting the Gavin+Krogan network with these inferred interactions. The ICD(Gavin+Krogan+Inferred) and FSW(Gavin+Krogan+Inferred) networks were generated by scoring this augmented network using the Iterative-CD and FS Weight systems, respectively.

## Methods

### The MCL-CAw algorithm: Identifying complexes embedded in the interaction network

Our MCL-CAw algorithm broadly consists of two phases. In the first phase, we partition the PPI network into multiple dense clusters using MCL. Following this (in the second phase), we post-process (refine) these clusters to obtain meaningful complexes. The MCL-CAw algorithm consists of the following steps:

1. Clustering the PPI network using MCL hierarchically

2. Categorizing proteins as cores within clusters

3. Filtering noisy clusters

4. Recruiting proteins as attachments into clusters

5. Extracting out complexes from clusters

6. Ranking the predicted complexes

We use the following notations while describing our algorithm. The PPI network is represented as *G *= (*V*, *E*), where *V *is the set of proteins, and E is the set of interactions between these proteins. For each *e *= *(p, q) *∈ *E*, there is a confidence score (weight) *w*(*p*, *q*) encoding the affinity between the proteins *p *and *q*. These affinity scores depend on the scoring system used.

#### Clustering the PPI network using MCL hierarchically

The first step of our algorithm is to partition (cluster) the PPI network using MCL [18], which simulates random walks (called a flow) to identify relatively dense regions in the network. The inflation coefficient parameter *I *in MCL is used to regulate the granularity of the clusters - higher the value more finer are the generated clusters (how to choose *I *in practice is discussed in the "Results" section). MCL tends to produce several large clusters (sizes ≥ 30) that amalgamate smaller clusters [7,20]. On the other hand, the size distributions of hand-curated complexes from Wodak lab [29], MIPS [30] and Aloy *et al. *[31] (Table 2) reveal that most complexes are of sizes less than 10. Therefore, we perform hierarchical clustering by iteratively selecting all clusters of sizes at least 30 and re-clustering them using MCL.

**Table 2:** Properties of hand-curated yeast complexes from Wodak lab [29], MIPS [30] and Aloy [31]

			*# Complexes of size*				
Benchmark	#Complexes	# Proteins	< 3	3-10	11-25	> 25	Avg density
Wodak	408	1627	172	204	27	5	0.639
MIPS	313	1225	106	138	42	27	0.412
Aloy	101	630	23	58	19	1	0.747

After iterative rounds of MCL-based hierarchical clustering on the protein network *G *= (*V*, *E*), we obtain a collection of *k *disjoint (non-overlapping) clusters {*C_i _*: *C_i _*= (*V_i_*, *E_i_*), 1 ≤ *i *≤ *k*}, where *V_i _*⊆ *V *and *E_i _*⊆ *E*.

#### Categorizing proteins as cores within clusters

Microarray analysis by Gavin *et al. *[6] of their predicted complex components showed that a large percentage of pairs of proteins within cores were co-expressed at the same time during the cell cycle and sporulation, consistent with the view that cores represent main functional units within complexes. Three-dimensional structural and yeast two-hybrid analysis showed that the core components were most likely to be in direct physical contact with each other. To reflect these findings in our post-processing steps, we expect:

• Every complex we predict to comprise of a non-empty set of core proteins; and

• The proteins within these cores to display relatively high degree of physical interactivity among themselves.

We identify the core proteins within a cluster in two stages: we first identify the set of preliminary cores and subsequently extend this to form the final set of cores. We categorize a protein *p*∈*V_i _*to be a 'preliminary core' protein in cluster *C_i _*= (*V_i_*, *E_i_*), given by *p *∈ *PCore*(*C_i_*), if:

• The *weighted in-connectivity of p with respect to C_i _*is at least the *average weighted in-connectivity of C_i_*, given by: *d_in_*(*p*, *C_i_*) ≥ *d_avg _*(*C_i_*); and

• The weighted in-connectivity of *p *with respect to *C_i _*is greater than the *weighted out-connectivity of p with respect to C_i_*, given by: *d_in_*(*p*, *C_i_*) >*d_out_*(*p*, *C_i_*).

The weighted in-connectivity *d_in_*(*p*, *C_i_*) of *p *with respect to *C_i _*is the total weight (score) of interactions *p *has with proteins within *C_i_*. Similarly, the weighted out-connectivity *d_out_*(*p*, *C_i_*) of *p *with respect to *C_i _*is the total weight of interactions *p *has with proteins outside *C_i_*. These are given by d*_in _*(*p*, *C_i_*) = ∑ {*w *(*p*, *q*): *q *∈ *V_i_*} and d*_out _*(*p*, *C_i_*) = ∑ {*w *(*p*, *q*): *q *∉ *V_i_*} respectively. The average weighted in-connectivity *d_avg_*(*C_i_*) of cluster *C_i _*is therefore the average of the weighted in-connectivities of all proteins within *Ci*, given by $d_{avg}(C_{i})=\frac{1}{\left|C_{i}\right|}\cdot \sum_{q\in V_{i}}d_{in}(q,\;C_{i})$.

We use these preliminary cores to find the 'extended core' proteins. We categorize a protein *p *∉ *PCore*(*C_i_*) to be an extended core protein in cluster *C_i_*, given by *p *∈ *ECore*(*C_i_*), if:

• *The weighted in-connectivity of p with respect to PCore*(*C_i_*) is at least the average of the weighted in-connectivities of all non-cores *r *∉ *PCore *(*C_i_*) to the preliminary cores, given by: *d_in _*(*p*, *PCore *(*C_i_*)) ≥ *d_avg _*(*r*, *PCore *(*C_i_*)); and

• The weighted in-connectivity of *p *with respect to *PCore*(*C_i_*) is greater than the *weighted out-connectivity of p with respect to PCore*(*C_i_*), given by: *d_in_*(*p*, *PCore*(*C_i_*)) >*d_out_*(*p, PCore*(*C_i_*)).

Here, *d_in_*(*p*, *PCore*(*C_i_*)) is the total weight of interactions *p *has with the preliminary cores of *C_i_*, given by: *d_in _*(*p*, *PCore *(*C_i_*)) = ∑ {*w *(*p*, *q*): *q *∈ *PCore *(*C_i_*)}. Similarly, *d_out_*(*p*, *PCore*(*C_i_*)) is the total weight of interactions *p *has with all the non-core proteins within *C_i_*, given by:

*d_in _*(*p*, *PCore *(*C_i_*)) = ∑ {*w *(*p*, *r*): *r *∈ *PCore *(*C_i_*)}. Finally, *d_avg_*(*r*, *PCore*(*C_i_*)) is the average weight of interactions of all non-cores *r *with the preliminary cores, given by:

davg(r, PCore(Ci))=1(|Ci|−|PCore(Ci)|)⋅∑r∈PCore(Ci)din(r, PCore(Ci)).

Combining the preliminary and extended core proteins, we form the final set of core proteins of cluster *C_i_*, given by:

(1) $$Core(C_{i})=\{PCore(C_{i})\cup ECore(C_{i})\}.$$

#### Filtering noisy clusters

Consistent with the assumption that every complex comprises of a set of core proteins, we consider a cluster as noisy if it does not include any core protein as per our above criteria. We discard all such noisy clusters.

#### Recruiting proteins as attachments into clusters

Microarray analysis by Gavin *et al. *[6] of their predicted complex components showed that attachment proteins were closely associated with core proteins within complexes and yet showed a greater degree of heterogeneity in expression levels, supporting the notion that attachments might represent non-stoichiometric components. Also, attachment proteins were seen shared between two or more complexes, consistent with the view that the same protein may participate in multiple complexes [20,21]. On the other hand, the application of MCL to PPI networks yields clusters that do not share proteins (non-overlapping clusters). Mapping these clusters back to the original PPI network shows that proteins having similar connectivities to multiple clusters are assigned arbitrarily to only one of the clusters. These proteins might as well be assigned to multiple clusters. To reflect these findings in our algorithm, we expect the attachment proteins to be those proteins within complexes that are:

• Non-core proteins;

• Closely interacting with the core proteins; and

• May be shared across multiple complexes.

We consider the following criteria to assign a non-core protein *p *belonging to a cluster *C_j _*(called donor cluster) as an attachment in an acceptor cluster *C_i _*(the donor and acceptor clusters may be the same), that is, *p *∈ Attach(*C_i_*):

• Protein *p *has sufficiently strong interactions with the core proteins *Core*(*C_i_*) of the cluster *C_i_*;

• The stronger the interactions among the core proteins, the stronger have to be the interactions of *p *with the core proteins;

• For large core sets, strong interactions are required to only some of the core proteins or, alternatively, weaker interactions to most of them.

Combining these criteria, we assign non-core p as an attachment in the acceptor cluster *C_i_*, that is *p *∈ *Attach*(*C_i_*), if:

(2) $$I_{p}\geq \alpha .I_{c}.{\left(\frac{S_{c}}{2}\right)}^{-\gamma },$$

where *I_p _*= *I*(*p*, *Core*(*C_i_*)) is the total weight of interactions of *p *with *Core*(*C_i_*), given by *I*(*p*, *Core*(*C_i_*)) = ∑{*w*(*p*, *q*): *q *∈ *Core*(*C_i_*)}, while *I_c _*= *I*(*Core*(*C_i_*)) is the total weight of interactions among the core proteins of *C_i_*, given by $I(Core(C_{i}))=\frac{1}{2}\cdot \sum \{w(q,\;r)\;:\;q,\;r\in Core(C_{i})\}$, and *S_c _*= |*Core*(*C_i_*)|, which is is normalized to yield 1 for core sets of size two. The parameters *α *and *γ *are used to control the effects of *I *(*Core*(*C_i_*)) and |*Core*(*C_i_*)|. For a simple illustration, let *α *= 0.5 and *γ *= 1, and consider all interactions to be of equal weight 1. Therefore, *p *is attached to a core set of four proteins, if the total weight of its interactions with the core proteins is at least 3, which is possible if *p *is connected to at least three core proteins (how to choose values for *α *and *γ *in practice is discussed in the "Results" section). This step ensures that non-core proteins having sufficiently strong interactions with the cores in more than one clusters are recruited as attachments into all those clusters.

#### Extracting out complexes from clusters

For each cluster we group together its constituent core and attachment proteins to define a unique complex. We expect all the remaining proteins within the cluster to have weaker associations with this resultant complex, and therefore categorize them as noisy proteins. In fact, experiments [28] have shown that MCL clusters tend to include several such noisy proteins leading to reduction in accuracies of the clusters. Therefore, our step ensures that such noisy proteins are discarded in order to extract out more accurate complexes. Additionally, since these resulting complexes include attachment proteins that potentially may be recruited by multiple complexes, this step ensures that our predicted complexes adhere to the protein-sharing phenomenon observed in real complexes [6,20,21]. We discard all complexes of size less than 4 because many of these are false positives. It is difficult to predict small real complexes solely based on interaction (topological) information (also noted in [16,24]).

For each cluster *C_i_*, we define a unique complex *Cmplx*(*C_i_*) as:

(3) $$Cmplx(C_{i})=\{Core(C_{i})\cup Attach(C_{i})\}.$$

Each interaction (*p*, *q*) among the constituent proteins *p *and *q *within this complex carries the weight *w*(*p*, *q*) observed in the PPI network.

#### Ranking the predicted complexes

As a final step, we output our predicted complexes in a reasonably meaningful order of biological significance. For this, we rank our predicted complexes in decreasing order of their weighted densities. The *weighted density *$WD({C^{\prime }}_{i})$ of a predicted complex ${C^{\prime }}_{i}$ is given by [16]:

(4) $$WD(C_{i}{}^{\prime })=\frac{\sum_{p,q\in {C^{\prime }}_{i}}w(p,q)}{\left|C_{i}^{\prime }|\cdot (|{C^{\prime }}_{i}|-1\right)}\text{.}$$

The *unweighted density *of a predicted complex is defined in a similar way by setting the weights of all constituent interactions to 1. This blindly favors very small complexes, or complexes with proteins having large number of interactions without considering the reliability of those interactions. On the other hand, the weighted density considers the reliability (by means of affinity scores) of such interactions. If two complexes have the same unweighted density, the complex with higher weighted density is ranked higher.

## Results

### Preparation of experimental data

We gathered high-confidence Gavin and Krogan-Core interactions deposited in BioGrid http://thebiogrid.org/[32] (version as of July 2009). These were assembled from a combination of bait-prey and prey-prey relationships (the spoke and matrix models) observed by Gavin *et al. *[6], and the bait-prey relationships (the spoke model) observed by Krogan *et al. *[7]. We combined these interactions to build the unscored Gavin+Krogan network (all edge-weights were set to 1). We then applied Iterative-CD*^k ^*[15,16] and FS Weight*^k ^*[14] scoring (with *k *= 2 iterations, recommended in [16]) on the Gavin+Krogan network, and selected all interactions with non-zero scores. This resulted in the ICD(Gavin+Krogan) and FSW(Gavin+Krogan) networks, respectively. In addition to these two scored networks, we downloaded the Consolidated_3.19 _network (with PE cut-off: 3.19, recommended by Collins *et al. *[11]) from http://interactome-cmp.ucsf.edu/, and the Bootstrap_0.094 _network [17] (with BT cut-off 0.094) from http://www.bio.ifi.lmu.de/Complexes/ProCope/. The Consolidated network was derived from the matrix modeled relationships of the original Gavin and Krogan datasets using the PE system [11]. Therefore, this network comprised of additional prey-prey interactions that were missed in the Krogan 'Core' dataset. The Bootstrap network was derived from the matrix modeled relationships using the bootstrapped scores [17]. Table 1 summarizes some properties of these networks.

The benchmark (reference) set of complexes was built from hand-curated complexes derived from three sources: 408 complexes of the Wodak lab CYC2008 catalogue [29], 313 complexes of MIPS [30], and 101 complexes curated by Aloy *et al. *[31]. The properties of these reference sets are shown in Table 2. We considered each of these reference sets independently for the evaluation of MCL-CAw. We did not merge them into one comprehensive list of complexes because the individual complex compositions are different across the three sources and some complexes may also get double-counted (because of different names used for the same complex). An alternative strategy was adopted by Wang *et al. *[21] by integrating the complexes from three sources (MIPS [30], SGD [33] and their own in-house curated complexes) using the Jaccard score: two complexes overlapping with a Jaccard score of at least 0.7 were merged together - the proteins to be included into the resultant complex were chosen based on a voting scheme.

To be accurate (as well as fair) while evaluating our method on these benchmark sets, we considered only the set of *derivable benchmark complexes *from each of the PPI networks: if a protein is not present in a PPI network, we remove it from the set of benchmark complexes. By repeated removals, if the size of a benchmark complex shrinks below 3, we remove the complex from our benchmark set to generate the final set of derivable benchmark complexes for each of the PPI networks.

In order to evaluate the biological coherence of our predicted complexes, we downloaded the list of cellular localizations (GO terms under "Cellular Component") of proteins from Gene Ontology (GO) [34]. We selected only the informative GO terms. A GO term is informative if no less than 30 proteins are annotated with this term and none of its descendant terms are annotated to no less than 30 proteins [35]. The list of essential genes was obtained from the *Saccharomyces *Genome Deletion Project [36,37]: http://www-sequence.stanford.edu/group/yeast_deletion_project/deletions3.html

### Evaluation metrics for matching predicted and benchmark complexes

Let *B *= {*B*_1_,*B*_2_,...,*B_m_*} and *C *= {*C*_1_,*C*_2_,...,*C_n_*} be the sets of benchmark and predicted complexes, respectively. We use the Jaccard coefficient *J *to quantify the overlap between a benchmark complex *B_i _*and a predicted complex *C_j _*:

(5) $$J(B_{i},C_{j})=\frac{\left|B_{i}\cap C_{j}\right|}{\left|B_{i}\int C_{j}\right|}\text{.}$$

We consider *B_i _*to be covered by *C_j_*, if *J*(*B_i_*, *C_j_*) ≥ *overlap threshold t*. In our experiments, we set the threshold *t *= 0.5, which requires $|B_{i}\cap C_{j}|\;\;\geq \frac{\left|B_{i}|+|C_{j}\right|}{3}$. For example, if |*B_i_*| = |*C_j _*| = 8, then the overlap between *B_i _*and *C_j _*should be at least 6.

We use previously reported [16] definitions of *recall Rc *(coverage) and *precision Pr *(sensitivity) of the set of predicted complexes:

(6) $$Rc=\frac{\left|\{B_{i}|B_{i}\in B\wedge \exists C_{j}\in C;J(B_{i},C_{j})\geq t\}\right|}{\left|B\right|}$$

Here, |{*B_i_*|*B_i _*∈ *B *Λ ∃*C_j _*∈ *C*; *J*(*B_i_*, *C_j_*) ≥ *t*}| gives the number of *derived benchmarks*.

(7) $$Pr=\frac{\left|\{C_{j}|C_{j}\in C\wedge \exists B_{i}\in B;J(B_{i},C_{j})\geq t\}\right|}{\left|C\right|}$$

Here, |{*C_j _*|*C_j _*∈ *C *Λ ∃*B_i _*∈ *B*; *J*(*B_i_*, *C_j_*) ≥ *t*}| gives the number of *matched predictions*.

We also evaluate the performance of our method by plotting the precision *versus *recall curves for the predicted complexes. These curves are plotted by tuning a threshold on the number of predicted complexes considered for the evaluation. The predicted complexes are considered in decreasing order of their weighted densities (that is, in increasing order of their complex ranks).

### Biological coherence of predicted complexes

A complex can be formed if its proteins are localized within the same compartment of the cell. So, we use the localization coherence of the predicted complexes as a measure their quality. Let *L *= {*L*_1_, *L*_2_,..., *L_k _*} be the set of known localization groups, where each *L_i _*contains a set of proteins with similar localization annotations. The *co-localization score LS*(*C_j_*) of a predicted complex *C_j _*is defined as the maximal fraction of its constituent proteins that are co-localized within the same localization group among the proteins that have annotations. This is given as follows [16]:

(8) $$LS(C_{j})=\frac{\max\{|C_{j}\cap L_{i}|:i=1,2,\ldots ,k\}}{\left|p:p\in C_{j}\wedge \exists L_{i}\in L,p\in L_{i}\right|}\text{.}$$

Therefore, the co-localization score *LS*(*C*) for the set of predicted complexes *C *is just the weighted average over all complexes [16]:

(9) $$LS(C)=\frac{\sum_{C_{j}\in C}\max\{|C_{j}\cap L_{i}|:i=1,2,\ldots ,k\}}{\sum_{C_{j}\in C}\left|p:p\in C_{j}\wedge \exists L_{i}\in L,p\in L_{i}\right|}\text{.}$$

### Setting the parameters *I*, *α *and *γ *for MCL-CAw

Before evaluating the performance of MCL-CAw, we describe the procedure used for setting inflation parameter *I *for MCL, and *α *and *γ *for core-attachment refinement in order to determine a good combination of parameters for MCL-CAw in practice. Only the predicted complexes of size ≥ 4 from MCL and MCL-CAw were considered for setting the parameters as well as for further experiments. We used F1 (harmonic mean of precision and recall) measured against the Wodak lab [29], MIPS [30] and Aloy [31] benchmarks as our basis for choosing the best values for these parameters.

We adopted the following four-step procedure for each PPI network:

1. Run MCL for a range of *I *values and choose *I *that offers the best F1 measure;

2. Set *I *to the chosen value, set a certain *α *for MCL-CAw, and choose *γ *from a range of values that offers the best F1 measure;

3. Set *I *and *γ *to the chosen values, and choose *α *for MCL-CAw from a range of values that offers the best F1 measure;

4. Set *α *and *γ *for MCL-CAw to the chosen values, and reconfirm the value chosen for *I*.

#### Setting I for MCL

**Figure 1:**
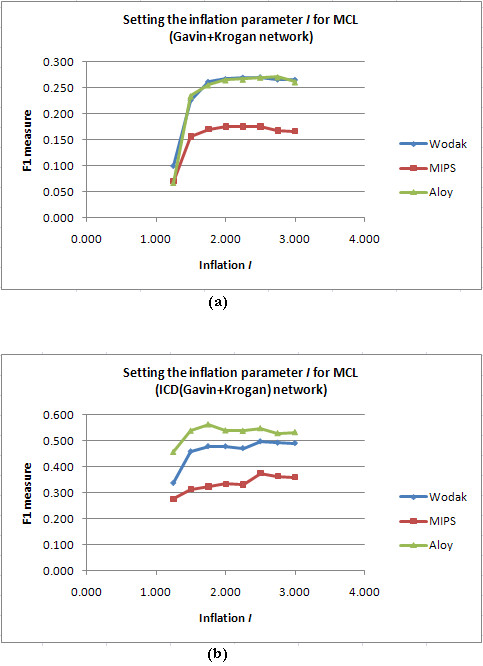
**Setting the inflation parameter *I *in MCL: F1 versus *I *plot**. (a): Plot for the unscored Gavin+Krogan network; (b): Plot for the scored ICD(Gavin+Krogan) network. *I *= 2.5 gave the best F1 for both unscored and scored networks.

Inflation *I *in MCL determines the granularity of the clustering - the higher the value more finer are the clusters produced. Typical values used for clustering PPI networks are *I *= 1.8 and 1.9 [16,19,38]. For each PPI network, we ran MCL over a range of *I *, and measured F1 against the three benchmark sets. We then normalized these F1 values against the best F1 obtained on each benchmark, summed up these normalized F1 values across benchmarks, and finally normalized these sums to obtain a final ranking for the *I *values. The detailed calculations are presented in Additional files 1, Tables S1 and S2. In Figure 1, we show sample F1 *versus I *plots for the unscored Gavin+Krogan and scored ICD(Gavin+Krogan) networks for the range of *I *= 1.25 to 3.0. We noticed that inflation *I *= 2.5 gave the best F1 on both unscored and scored networks. The F1 obtained at *I *= 1.8 and 1.9 was only marginally less than that at *I *= 2.5.

#### Setting α and γ for CA refinement

**Figure 2:**
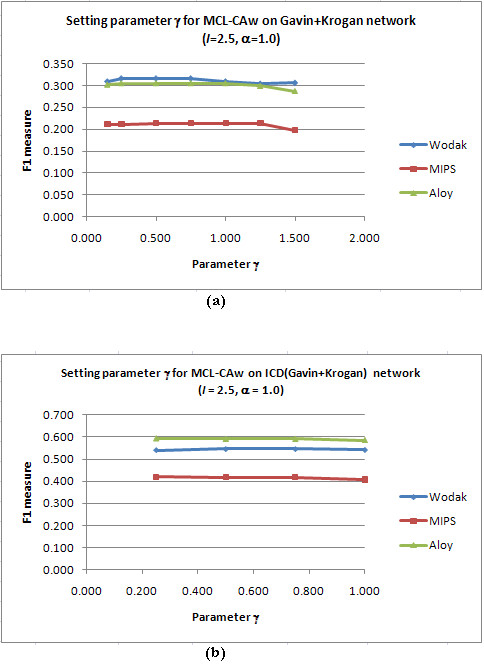
**Setting the parameter *γ *in core-attachment refinement: F1 versus *γ *plot**. (a): Plot for the unscored Gavin+Krogan network; (b): Plot for the scored ICD(Gavin+Krogan) network. *γ *= 0.75 gave the best F1 for both unscored and scored networks (*I *= 2.50 and *α *= 1.00).

For each PPI network, we set *I *to the chosen value, fixed a certain *α*, and ran MCL-CAw over a range of *γ*. We adopted the same method as above to choose the value of *γ *offering the best F1 measure. Figure 2 shows sample F1 *versus γ *plots on the unscored Gavin+Krogan and scored ICD(Gavin+Krogan) networks for *I *= 2.5, *α *= 1.00 and *γ *= 0.15 to 1.50. The detailed calculations are presented in Additional files 1, Table S3. We noticed that *γ *= 0.75 gave the best F1 on both unscored and scored networks.

**Figure 3:**
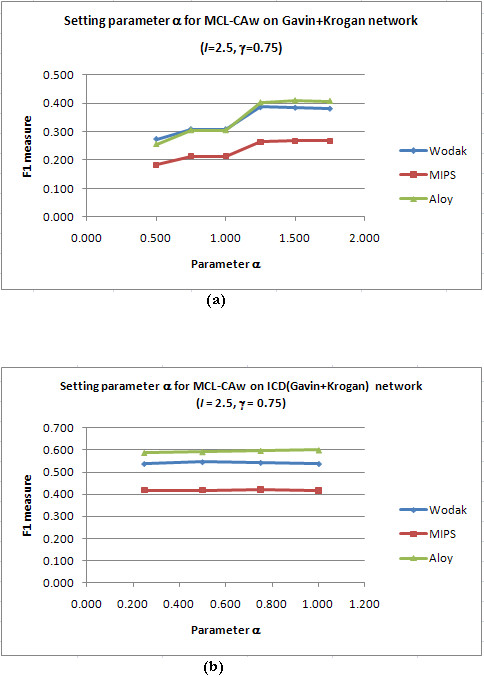
**Setting the parameter *α *in core-attachment refinement: F1 versus *α *plot**. (a): Plot for the unscored Gavin+Krogan network; (b): Plot for the scored ICD(Gavin+Krogan) network. *α *= 1.50 gave the best F1 for the unscored network (*I *= 2.50 and *γ *= 0.75). *α *= 1.00 gave the best F1 for the scored networks (*I *= 2.50 and *γ *= 0.75)..

Next, we set *I *and *γ *to the chosen values, and ran MCL-CAw over a range of α. Figure 3 shows sample F1 *versus α *plots on the unscored Gavin+Krogan and scored ICD(Gavin+Krogan) networks for *I *= 2.5, = *γ *= 0.75 and *α *= 0.50 to 1.75. The detailed calculations are presented in Additional files 1, Table S4. We noticed that *α *= 1.50 gave the best F1 on the unscored network, while *α *= 1.0 gave the best F1 on the scored networks.

#### Reconfirming I for the chosen values of α and γ

**Figure 4:**
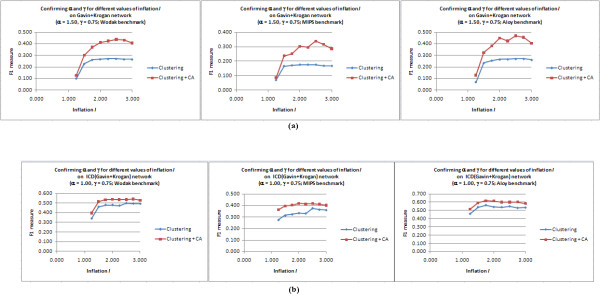
**Reconfirming inflation *I *for MCL-Caw**. (a): Plot for the unscored Gavin+Krogan network with *α *= 1.50 and *γ *= 0.75. (b): Plot for the scored ICD(Gavin+Krogan) network *α *= 1.00 and *γ *= 0.75. *I *= 2.5 gave the best F1 for these chosen values of *α *and *γ *(except on the Aloy benchmark, the smallest benchmark among the three, on which *I *= 1.75 gave marginally better F1).

Finally, for each PPI network, we ran core-attachment refinement with the chosen values of *α *and *γ *over a range of *I *for MCL. Figure 4 compares the F1 *versus I *plots for plain-MCL and MCL followed by CA refinement on the unscored Gavin+Krogan and scored ICD(Gavin+Krogan) networks for range *I *= 1.25 to 3.0. The plots reconfirmed that the chosen values for *α *and *γ *gave the best performance for CA refinement when *I *= 2.5 (except for the Aloy benchmark, the smallest benchmark among the three, for which F1 was best at *I *= 1.75 and was marginally lower for *I *= 2.5). The detailed calculations are presented in Additional files 1, Tables S5 and S6. We settled on *I *= 2.5, *α *= 1.50 and *γ *= 0.75 for the unscored Gavin+Krogan network, and *I *= 2.5, *α *= 1.0 and *γ *= 0.75 for the scored networks as our final combination of parameters for MCL-CAw.

### Evaluating the performance of MCL-CAw

**Figure 5:**
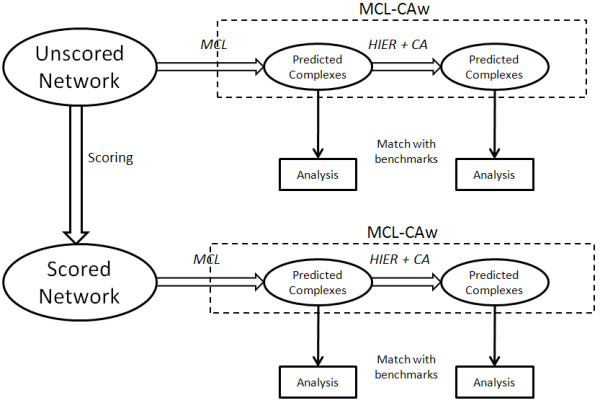
**Workflow for evaluation of MCL-Caw**. The predicted complexes of MCL-CAw were tapped at two stages: (i) Clustering using MCL; (ii) Hierarchical clustering followed by core-attachment refinement using MCL-CAw. These predicted complexes were evaluated by matching them to the set of benchmark complexes.

Figure 5 shows the *workflow *considered for the evaluation of MCL-CAw. The predicted complexes were tapped at two successive stages:

1. After clustering using MCL;

2. After hierarchical clustering followed by core-attachment refinement using MCL-CAw.

#### The effect of core-attachment refinement on the predictions of MCL

Compare the *topmost rows *in Table 3 for MCL and MCL-CAw evaluated on the unscored Gavin+Krogan network. They show that MCL-CAw achieved significantly higher recall compared to MCL on Gavin+Krogan - on an average 31% higher number of complexes derived than MCL. In fact referring back to Figure 4(a), MCL-CAw achieved higher F1 compared to MCL for the entire range *I *= 1.25 to 3.00. In order to further analyse this improvement, we considered two sets of complexes derived from Gavin+Krogan. (a) Set *A *= MCL ∩ MCL-CAw, consisting of all complexes correctly predicted by both MCL and MCL-CAw, but with different Jaccard accuracies; (b) Set *B *= MCL-CAw\MCL, consisting of all complexes correctly predicted by MCL-CAw, but not by MCL. There was no complex correctly predicted by MCL that was missed by MCL-CAw. We calculated the increase (percentage) in accuracies for complexes from *A *and *B*. This increase for *A *was noticably high, the average being 7.53% on the Wodak set. The increase for *B *was significantly high, the average being 62.26% on the Wodak set. This shows: (a) CA-refinement was successful in improving the accuracies of MCL clusters; (b) This improvement was particularly high for low quality clusters of MCL (that is, set B). MCL-CAw was successful in elevating the accuracies above the threshold *t *= 0.5 for those clusters that were difficult to be matched to known complexes using MCL alone. Consequently, MCL-CAw derived significantly higher number of benchmark complexes than MCL.

**Table 3:** (i) Impact of core-attachment refinement on MCL; (ii) Role of affinity scoring in reducing the impact of natural noise on MCL and MCL-CAw

Benchmark	Method	PPI Network	#Predicted complexes	#Matched prediction	Precision	#Derivable benchmarks	#Derived benchmarks	Recall
**Wodak**	MCL	G+K	242	55	0.226	182	62	0.338
		ICD(G+K)	136	68	0.500	153	76	0.497
		FSW(G+K)	120	69	0.575	153	78	0.510
		Consol_3.19_	116	70	0.603	145	79	0.545
		Boot_0.094_	203	76	0.374	172	85	0.494
	
	MCL-CAw	G+K	310	77	0.248	182	77	0.423
		ICD(G+K)	129	80	0.620	153	80	0.523
		FSW(G+K)	117	72	0.615	153	83	0.542
		Consol_3.19_	122	82	0.672	145	82	0.566
		Boot_0.094_	199	79	0.397	172	88	0.512

**MIPS**	MCL	G+K	242	35	0.143	177	40	0.226
		ICD(G+K)	136	47	0.346	151	60	0.397
		FSW(G+K)	120	46	0.383	151	61	0.404
		Consol_3.19_	116	48	0.414	157	63	0.401
		Boot_0.094_	203	44	0.271	168	56	0.333
	
	MCL-CAw	G+K	310	53	0.171	177	53	0.300
		ICD(G+K)	129	63	0.488	151	63	0.417
		FSW(G+K)	117	48	0.410	151	66	0.437
		Consol_3.19_	122	68	0.557	157	68	0.433
		Boot_0.094_	199	47	0.236	168	59	0.351

**Aloy**	MCL	G+K	242	43	0.179	76	42	0.556
		ICD(G+K)	136	58	0.426	75	56	0.747
		FSW(G+K)	120	57	0.475	75	57	0.760
		Consol_3.19_	116	54	0.466	76	55	0.724
		Boot_0.094_	203	56	0.276	76	55	0.724
	
	MCL-CAw	G+K	310	52	0.168	76	52	0.684
		ICD(G+K)	129	59	0.457	75	59	0.787
		FSW(G+K)	117	60	0.513	75	60	0.800
		Consol_3.19_	122	57	0.467	76	57	0.750
		Boot_0.094_	199	57	0.286	76	58	0.763

Affinity scoring of PPI networks improved the performance of MCL and MCL-CAw. Affinity scoring followed by CA refinement had a compounded effect in improving the performance of MCL.

#### Impact of noise on MCL and MCL-CAw and the role of affinity scoring in reducing this impact

**Figure 6:**
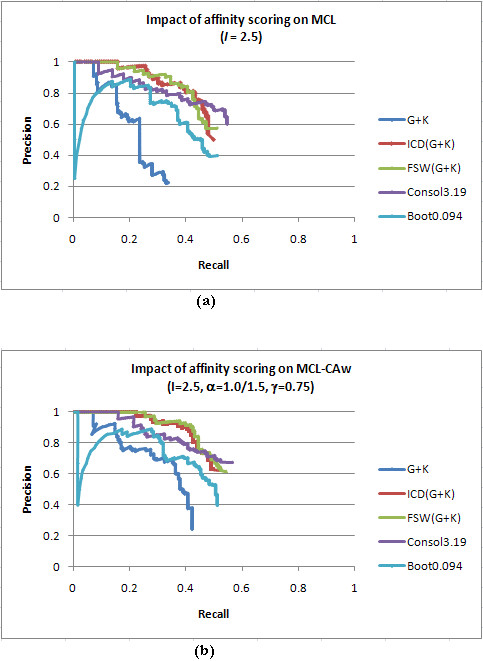
**Impact of affinity scoring on the performance of MCL and MCL-Caw**. (a). and (b): Precision *versus *recall curves on Gavin+Krogan and the four scored networks (ICD(Gavin+Krogan), FSW(Gavin+Krogan), Consolidated_3.19 _and Bootstrap_0.094_) for MCL and MCL-CAw, respectively, evaluated on Wodak benchmark with *t *= 0.5. Both the methods showed significant improvement on the scored networks compared to unscored Gavin+Krogan.

Table 3 compares different evaluation metrics for MCL and MCL-CAw on the unscored Gavin+Krogan with the four scored PPI networks. Very clearly, both MCL and MCL-CAw showed considerable improvement in precision and recall on the scored networks. For example, MCL achieved about 127% higher precision and 51.3% higher recall (on average), while MCL-CAw achieved about 132% higher precision and 26.6% higher recall (on average on Wodak lab benchmark) on the four scored networks than on the unscored Gavin+Krogan network. The precision *versus *recall curves (Figure 6) on Gavin+Krogan dropped sharply, while those for the three scored networks - ICD(Gavin+Krogan), FSW (Gavin+Krogan) and Consolidated_3.19 _- displayed a more "graceful" decline. The curve for Bootstrap_0.094 _displayed a sudden dip towards the beginning, but stabilized subsequently to achieve a higher (final) precision and recall compared to the unscored Gavin+Krogan network.

Among the four scored PPI networks, both MCL and MCL-CAw showed best precision and recall on the Consolidated_3.19 _network, which can be directly attributed to the high quality of this network. However, this high quality of Consolidated_3.19 _came at the expense of lower protein coverage (see Table 4; also noted in [20]), resulting in reduced number of derivable complexes. In order to counter this, we gathered a larger subset of the Consolidated network with PE cut-off 0.623 (the average PE score), which accounted for a higher protein coverage (Table 4). We noticed that the improvement of MCL-CAw over MCL was significantly higher on Consolidated_0.623_, compared to the improvement seen on Consolidated_3.19_. We also noticed that ICD scoring of Consolidated_0.623 _drastically reduced the size of this network, revealing that this larger subset in fact included significant amount of false positives (noise). These experiments indicate that any reasonably good algorithm like MCL can perform well on high quality networks. However, due to the lack of protein coverage as well as scarcity of such high quality networks, we need to consider larger networks for complex detection (particularly to be able to detect novel complexes). This in turn exposes the algorithms to higher amount of natural noise (even in scored networks). Therefore, the need is to develop algorithms that can detect larger number of complexes in the presence of such noise. In this scenario, our results show that MCL-CAw is able to derive considerably higher number of complexes than MCL. Taking this further, we introduced different levels of random noise to study its impact on MCL and MCL-CAw. We introduced 10% to 75% random noise (2000 to 10000 random interactions) to the Gavin+Krogan network. We noticed that MCL-CAw performed better than MCL even upon introducing 50% random noise (Table 5). However, at 75% random noise, the performance of MCL-CAw marginally dropped below that of MCL. Therefore, MCL-CAw was reasonably robust to random noise - it was stable in the range 10% - 40% noise, which covers the typical levels of noise seen in TAP/MS datasets [9] (we say this keeping in mind that MCL has been shown to be robust even at 80% random noise [38]). We next scored these noisy networks using the ICD scheme. We found that the performance of both MCL and MCL-CAw improved considerably on these scored networks. MCL-CAw performed considerably better than MCL even at 50% to 75% random noise (Table 5). Therefore, affinity scoring helped MCL-CAw to maintain its performance gain over MCL.

**Table 4:** MCL-CAw performed considerably better than MCL in the presence of natural noise

PPI Network	#Proteins	#Interactions	Avg node deg	#Derived MCL	complexes (Recall) MCL-CAw
Consol_3.19_	1622	9704	11.96	79 (0.545)	82 (0.566)
Consol_0.623_	5423	102393	37.76	74 (0.321)	84 (0.375)

ICD(Cons_3.19_)	1161	8688	14.96	58 (0.408)	63 (0.443)
ICD(Cons_0.623_)	1273	19996	31.41	52 (0.353)	56 (0.381)

FSW(Cons_3.19_)	1123	8694	15.48	59 (0.401)	65 (0.442)
FSW(Cons_0.623_)	1341	20696	30.87	54 (0.360)	57 (0.380)

The Consolidated_3.19 _and Consolidated_0.623 _networks were subsets of the Consolidated network [11] derived with PE cut-offs 3.19 and 0.623, respectively. We ran ICD and FSW schemes on these networks. Consolidated_0.623 _had significant amount of false positives (about 81%) that were discarded by the scoring. The performance of MCL-CAw was only marginally better than MCL on Consolidated_3.19_, but MCL-CAw performed considerably better than MCL on the "more noisy" Consolidated_0.623_.

**Table 5:** (i) Impact of introducing different levels of artificial noise on MCL and MCL-CAw (ii) Role of affinity scoring in reducing the impact of noise

Method	PPI Network	#Predicted complexes	#Matched predictions	Precisions	#Derivable benchmarks	#Derived benchmarks	Recall
MCL	G+K	242	55	0.226	182	62	0.338
	G+K+Rand2k	265	56	0.215	182	64	0.352
	G+K+Rand5k	274	61	0.223	182	68	0.379
	G+K+Rand10k	316	64	0.202	182	69	0.379
	ICD(G+K)	119	73	0.613	153	73	0.477
	ICD(G+K+Rand2k)	104	59	0.567	153	66	0.431
	ICD(G+K+Rand5k)	108	60	0.546	151	65	0.430
	ICD(G+K+Rand10k)	112	60	0.546	150	65	0.433

MCL-CAw	G+K	310	77	0.248	182	77	0.423
	G+K+Rand2k	140	59	0.421	182	68	0.374
	G+K+Rand5k	116	62	0.534	182	70	0.384
	G+K+Rand10k	176	64	0.363	182	68	0.373
	ICD(G+K)	129	80	0.620	153	80	0.523
	ICD(G+K+Rand2k)	102	62	0.608	153	73	0.477
	ICD(G+K+Rand5k)	102	64	0.627	151	76	0.503
	ICD(G+K+Rand10k)	106	64	0.603	150	76	0.506

The Gavin+Krogan network was introduced with 2000 - 10000 (10% to 75%) random interactions. Following this, these noisy networks were scored using the ICD scheme. With the aid of scoring, MCL-CAw was able to perform better than MCL even at 50% random noise.

#### Biological coherence of predicted components

The co-localization scores for the various predicted components (cores and whole complexes) of MCL-CAw are shown in Table 6. The table shows that: (a) The predicted complexes of MCL-CAw showed high co-localization scores compared to MCL on both the unscored and scored PPI networks. MCL included several noisy proteins into the predicted clusters, thereby reducing their biological coherence; (b) The predicted cores of MCL-CAw displayed higher scores compared to complexes, indicating that proteins within cores were highly localized; (c) The complexes of both MCL and MCL-CAw displayed higher scores on the four scored networks compared to the Gavin+Krogan network.

**Table 6:** Co-localization scores for predicted components from MCL and MCL-CAw

	*Co-localization scores*		
PPI Network	MCL clusters	MCL-CAw cores	MCL-CAw complexes
Gavin+Krogan	0.730	0.890	0.866
ICD(Gavin+Krogan)	0.830	0.936	0.912
FSW(Gavin+Krogan)	0.830	0.931	0.912
Consolidated_3.19_	0.790	0.923	0.908
Bootstrap_0.094_	0.788	0.895	0.874

Findings: (i) The complexes produced after CA-refinement showed higher scores than those of MCL; (ii) The complexes predicted from the scored networks showed higher scores than from the Gavin+Krogan network; (iii) The cores in MCL-CAw showed higher scores than whole complexes.

### Relative ranking of complex prediction algorithms and affinity-scored networks

In order to gauge the performance of MCL-CAw relative to existing techniques, we selected the following recent algorithms proposed for complex detection:

• On the unscored Gavin+Krogan network, we compared against MCL [18,19], our preliminary work MCL-CA (2009) [28], CORE by Leung *et al. *(2009) [24], COACH by Wu Min *et al. *(2009) [25], CMC by Liu *et al. *(2009) [16], and HACO by Wang *et al. *(2009) [21];

• On the affinity-scored networks, we compared against MCL, MCL incorporated with cluster overlaps by Pu *et al. *(2007) [20] (our implementation of this, called MCLO), CMC and HACO.

Table 7 summarizes some of the properties and the parameters used for these methods. We consider only complexes of size at least 4 from all algorithms in this entire evaluation. We dropped MCL-CA, CORE and COACH for the comparisons on the affinity-scored networks because these methods assume unweighted networks as inputs. Further, we do not show results for older methods namely MCODE by Bader and Hogue (2003) [8] and RNSC by King *et al. *(2004) [39], instead include MCL into all our comparisons, because MCL significantly outperforms these methods [16,38].

**Table 7:** Existing complex detection methods selected for comparisons with MCL-CAw

	*Method*						
Property	MCL	MCL-CA	MCLO	CORE	COACH	CMC	HACO
**Principle**	Flow simulation	Core-attach refinement over MCL	MCL with cluster overlaps	Core-attach by *p*-values	Core-attach by dense neighborhood	Maximal clique merging	Hier agglo cluster with overlaps
**Scored Networks**	Yes	No	Yes	No	No	Yes	Yes
**Unassigned Proteins**	No	Yes	No	Yes	Yes	Yes	Yes
**Parameters** **(default)**	Inflation *I* (*I *= 2.5)	Inflation *I* (*I *= 2.5)	Inflation *I *, Overlap *a, b *(2.5, 1.0, 0.5)	/	Filter *t* (*t *= 0.225)	Merge *m*, Overlap *t*, Min clust size (0.5, 0.25, 4)	UPGMA cutoff (0.2)
**References**	Dongen 2000 [18]	Srihari *et al*. 2009 [28]	Pu *et al*. 2007 [20]	Leung *et al*. 2009 [24]	Wu Min *et al*. 2009 [25]	Liu *et al*. 2009 [16]	Wang *et al*. 2009 [21]

CORE (2009), COACH (2009), MCL-CA (2009) were compared against MCL-CAw only on the unscored Gavin+Krogan network, while MCL (2000, 2002), MCLO (2007), CMC (2009) and HACO (2009) were evaluated also on the scored networks.

**Figure 7:**
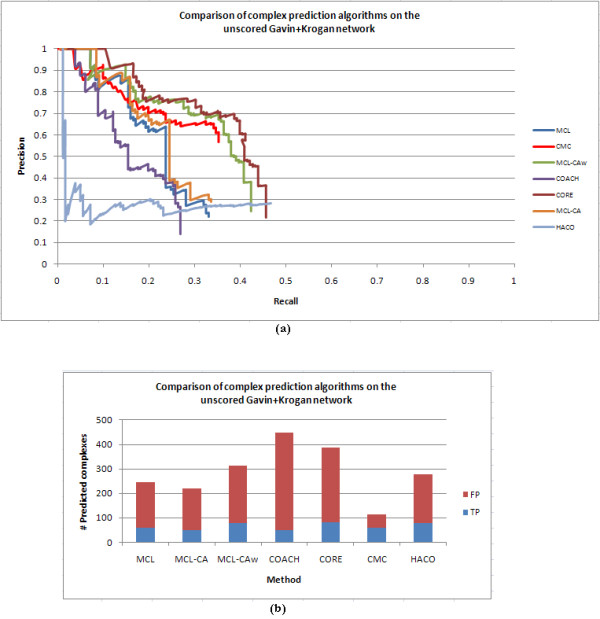
**Comparison of complex detection algorithms on unscored Gavin+Krogan network**. (a): Precision *versus *recall curves and area-under-the-curve (AUC) values for complex detection algorithms on the unscored Gavin+Krogan network, evaluated on Wodak reference with *t *= 0.5. AUC for MCL = 0.225, COACH = 0.169, CORE = 0.361, MCL-CAw = 0.323, CMC = 0.271, MCL-CA = 0.238, HACO = 0.136. (b): Number of predicted complexes, proportion of true positives (correctly matched to benchmark(s)) and false positives (not matched to any benchmark) for the algorithms.

**Figure 8:**
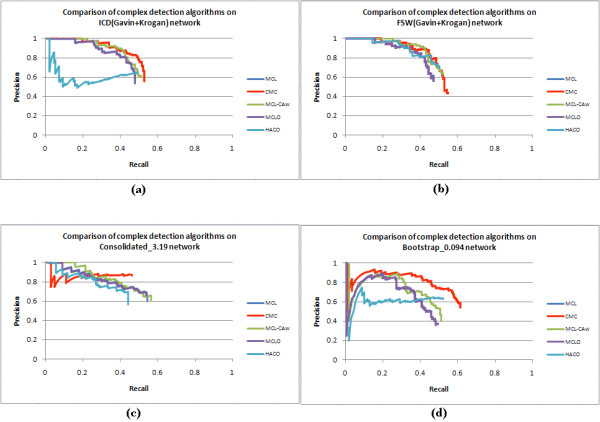
**Comparison of complex detection algorithms on four scored networks**. Precision *versus *recall curves and area-under-the-curve (AUC) values for complex detection algorithms evaluated on Wodak reference with *t *= 0.5. (a) ICD(Gavin+Krogan): AUC values MCL = 0.436, CMC = 0.494, MCL-CAw = 0.472, MCLO = 0.435, HACO = 0.305. (b) FSW(Gavin+Krogan): AUC values MCL = 0.431, CMC = 0.481, MCL-CAw = 0.487, MCLO = 0.430, HACO = 0.461. (c) Consolidated_3.19_: AUC values MCL = 0.469, CMC = 0.399, MCL-CAw = 0.488, MCLO = 0.463, HACO = 0.367. (d) Bootstrap_0.094_: AUC values MCL = 0.349, CMC = 0.513, MCL-CAw = 0.389, MCLO = 0.353, HACO = 0.317.

Tables 8, 9, 10, 11 and 12 show detailed comparisons between complex detection algorithms on the unscored and scored networks. Figures 7 and 8 show the precision versus recall curves on these networks, while Table 13 shows the area-under-the-curve (AUC) values for these curves. Considering ± 5% error in AUC values, the table shows that CORE attained the highest AUC followed by MCL-CAw and CMC on the unscored network, while MCL-CAw and CMC achieved the overall highest AUC on the scored networks. In addition to this, on each network we ranked the algorithms based on their normalized final F1 measures (with respect to the best performing algorithm on that network), as shown in Table 14. We summed up the normalized F1 values for each algorithm across all the networks to obtain an overall ranking of the algorithms as shown in Table 15. The detailed calculations are presented in Additional files 1, Table S7. On the unscored network CMC showed the best F1 value, while on the scored networks MCL-CAw showed the best overall F1 value. In particular, MCL-CAw performed the best on ICD(Gavin+Krogan), FSW(Gavin+Krogan) and Consolidated_3.19 _networks, while HACO performed the best on Bootstrap_0.094_. This more or less agreed with the relative performance gathered from the AUC values (Table 13).

**Table 8:** Comparisons between the different methods on the unscored Gavin+Krogan network

		*Method*						
		MCL	MCL-CA	MCL-CAw	COACH	CORE	CMC	HACO
		
	#Predicted	242	219	310	447	386	113	278
**Wodak** **(#182)**	#Matched	55	49	77	62	83	60	78
	Precision	0.226	0.224	0.248	0.139	0.215	0.531	0.281
	#Derived	62	49	77	49	83	60	85
	Recall	0.338	0.269	0.423	0.269	0.456	0.330	0.467

**MIPS** **(#177)**	#Matched	35	42	53	45	59	41	45
	Precision	0.143	0.192	0.171	0.101	0.153	0.363	0.162
	#Derived	40	42	53	38	59	41	57
	Recall	0.226	0.237	0.300	0.215	0.333	0.232	0.322

**Aloy** **(#76)**	#Matched	43	41	52	54	59	43	59
	Precision	0.179	0.187	0.168	0.121	0.153	0.381	0.212
	#Derived	42	41	52	37	59	43	59
	Recall	0.556	0.539	0.684	0.487	0.776	0.566	0.776

Methods considered: MCL, MCL-CA, MCL-CAw, COACH, CORE, CMC and HACO. CMC performed the best in terms of precision, while HACO and CORE performed the best in terms of recall. MCL-CAw stood third among of the seven algorithms in both precision and recall. #Matched: #Predictions matching some benchmark complex(es). #Derived: #Benchmark complexes derived by some predicted complex(es).

The unscored Gavin+Krogan network

#Proteins 2964; #Interactions 13507

**Table 9:** Comparisons between the different methods on the ICD(Gavin+Krogan) network

		*Method*				
		MCL	MCLO	MCL-CAw	CMC	HACO
		
	#Predicted	136	121	129	171	104
**Wodak** **(#153)**	#Matched	68	73	80	86	68
	Precision	0.500	0.603	0.620	0.503	0.654
	#Derived	76	73	80	86	76
	Recall	0.497	0.477	0.523	0.562	0.497

**MIPS'** **(#151)**	#Matched	47	56	63	65	41
	Precision	0.346	0.463	0.488	0.380	0.394
	#Derived	60	56	63	65	55
	Recall	0.397	0.371	0.417	0.430	0.364

**Aloy** **(#75)**	#Matched	58	56	59	59	53
	Precision	0.426	0.463	0.457	0.345	0.510
	#Derived	56	56	59	59	53
	Recall	0.747	0.747	0.787	0.787	0.707

Methods considered: MCL, MCLO, MCL-CAw, CMC and HACO. HACO performed the best in terms of precision, while CMC performed the best in terms of recall. MCL-CAw was a close second in both precision and recall. #Matched: #Predictions matching some benchmark complex(es). #Derived: #Benchmark complexes derived by some predicted complex(es).

The ICD(Gavin+Krogan) network

#Proteins 1628; #Interactions 8707

**Table 10:** Comparisons between the different methods on the FSW(Gavin+Krogan) network

		*Method*				
		MCL	MCLO	MCL-CAw	CMC	HACO
		
	#Predicted	120	108	117	176	99
**Wodak** **(#153)**	#Matched	69	61	72	76	68
	Precision	0.575	0.564	0.615	0.432	0.687
	#Derived	78	72	83	84	77
	Recall	0.510	0.471	0.542	0.549	0.503

**MIPS** **(#151)**	#Matched	46	42	48	49	42
	Precision	0.383	0.388	0.410	0.278	0.424
	#Derived	61	55	66	65	56
	Recall	0.404	0.364	0.437	0.430	0.371

**Aloy** **(#75)**	#Matched	57	56	60	59	53
	Precision	0.475	0.518	0.513	0.335	0.535
	#Derived	57	56	60	57	53
	Recall	0.760	0.747	0.800	0.760	0.707

Methods considered: MCL, MCLO, MCL-CAw, CMC and HACO. HACO performed the best in terms of precision, while MCL-CAw and CMC performed the best in terms of recall. MCL-CAw was a close second in terms of precision. #Matched: #Predictions matching some benchmark complex(es). #Derived: #Benchmark complexes derived by some predicted complex(es).

The FSW(Gavin+Krogan) network

#Proteins 1628; #Interactions 8688

**Table 11:** Comparisons between the different methods on the Consolidated_3.19 _network

		*Method*				
		MCL	MCLO	MCL-CAw	CMC	HACO
		
	#Predicted	116	119	122	77	101
**Wodak** **(#145)**	#Matched	70	80	82	67	57
	Precision	0.603	0.672	0.672	0.870	0.564
	#Derived	79	80	82	67	64
	Recall	0.545	0.552	0.566	0.462	0.441

**MIPS** **(#157)**	#Matched	48	65	68	56	40
	Precision	0.414	0.546	0.557	0.727	0.396
	#Derived	63	65	68	56	57
	Recall	0.401	0.414	0.433	0.357	0.363

**Aloy** **(#76)**	#Matched	54	56	57	45	44
	Precision	0.466	0.471	0.467	0.584	0.436
	#Derived	55	56	57	45	45
	Recall	0.724	0.737	0.750	0.592	0.592

Methods considered: MCL, MCLO, MCL-CAw, CMC and HACO. CMC performed the best in terms of precision, while MCL-CAw performed the best in recall. #Matched: #Predictions matching some benchmark complex(es). #Derived: #Benchmark complexes derived by some predicted complex(es).

*The Consolidated*_3.19 _*network*

#Proteins 1622; #Interactions 9704

**Table 12:** Comparisons between the different methods on the Bootstrap_0.094 _network

		*Method*				
		MCL	MCLO	MCL-CAw	CMC	HACO
		
	#Predicted	203	204	199	203	127
**Wodak** **(#172)**	#Matched	76	76	79	110	80
	Precision	0.374	0.372	0.397	0.542	0.630
	#Derived	85	85	88	106	90
	Recall	0.494	0.494	0.512	0.616	0.523

**MIPS** **(#168)**	#Matched	44	45	47	67	49
	Precision	0.271	0.220	0.236	0.330	0.386
	#Derived	56	57	59	69	63
	Recall	0.333	0.339	0.351	0.411	0.375

**Aloy** **(#76)**	#Matched	56	55	57	76	59
	Precision	0.276	0.269	0.286	0.374	0.465
	#Derived	55	55	58	63	60
	Recall	0.724	0.723	0.763	0.829	0.789

Methods considered: MCL, MCLO, MCL-CAw, CMC and HACO. HACO performed the best in terms of precision, while CMC performed the best in terms of recall. MCL-CAw was positioned third in both precision and recall. #Matched: #Predictions matching some benchmark complex(es). #Derived: #Benchmark complexes derived by some predicted complex(es).

*The Bootstrap*_0.094 _*network*

#Proteins 2719; #Interactions 10290

**Table 13:** Area under the curve (AUC) values of precision versus recall curves for complex detection methods on the unscored and scored PPI networks

PPI network	MCL	MCLO	MCL-CAw	CMC	HACO	COACH	CORE
G+K	0.225		0.323	0.271	0.136	0.169	**0.361**

ICD(G+K)	0.436	0.435	0.472	**0.494**	0.305		
FSW(G+K)	0.431	0.430	**0.487**	0.481	0.461		
Consol_3.19_	0.469	0.463	**0.488**	0.399	0.367		
Boot_0.094_	0.349	0.353	0.389	**0.513**	0.317		

Considering ± 5% error in AUC values, CORE showed the highest value on unscored network, while MCL-CAw and CMC showed the overall highest on the scored networks.

**Table 14:** Relative ranking of complex detection algorithms on unscored and affinity-scored networks

PPI network		Wodak		MIPS		Aloy			
	Method	F1	Norm	F1	Norm	F1	Norm	Total	Norm
G+K	CMC	0.407	1.000	0.283	1.000	0.455	1.000	3.000	1.000
	HACO	0.351	0.862	0.216	0.761	0.333	0.731	2.355	0.785
	MCL-CAw	0.313	0.768	0.218	0.770	0.270	0.592	2.130	0.710
	CORE	0.292	0.718	0.210	0.741	0.256	0.561	2.020	0.673
	MCL	0.271	0.665	0.175	0.619	0.271	0.595	1.879	0.626
	MCL-CA	0.244	0.601	0.212	0.749	0.278	0.610	1.960	0.653
	COACH	0.183	0.450	0.137	0.486	0.194	0.426	1.361	0.454

ICD(G+K)	MCL-CAw	0.567	1.000	0.450	1.000	0.578	0.976	2.976	1.000
	HACO	0.565	0.995	0.378	0.841	0.593	1.000	2.837	0.953
	MCLO	0.533	0.939	0.412	0.916	0.572	0.965	2.820	0.947
	CMC	0.531	0.936	0.403	0.897	0.480	0.810	2.642	0.888
	MCL	0.498	0.879	0.370	0.822	0.543	0.916	2.616	0.879

FSW(G+K)	MCL-CAw	0.576	0.992	0.423	1.000	0.625	1.000	2.992	1.000
	HACO	0.581	1.000	0.396	0.935	0.609	0.974	2.910	0.972
	MCL	0.541	0.931	0.393	0.929	0.585	0.935	2.795	0.934
	MCLO	0.513	0.884	0.376	0.888	0.612	0.979	2.750	0.919
	CMC	0.484	0.833	0.338	0.798	0.465	0.744	2.375	0.794

Cons_3.19_	MCL-CAw	0.614	1.000	0.487	1.000	0.576	0.979	2.979	1.000
	MCLO	0.606	0.986	0.471	0.967	0.575	0.977	2.930	0.984
	CMC	0.604	0.982	0.479	0.983	0.588	1.000	2.965	0.995
	MCL	0.573	0.932	0.407	0.836	0.567	0.964	2.732	0.917
	HACO	0.475	0.774	0.379	0.777	0.502	0.854	2.405	0.807

Boot_0.094_	HACO	0.572	0.991	0.380	1.000	0.585	1.000	2.991	1.000
	CMC	0.577	1.000	0.367	0.965	0.515	0.881	2.846	0.952
	MCL-CAw	0.447	0.776	0.282	0.742	0.416	0.711	2.229	0.745
	MCL	0.426	0.738	0.299	0.785	0.400	0.683	2.207	0.738
	MCLO	0.424	0.736	0.267	0.701	0.392	0.670	2.108	0.705

Ranking obtained from normalized F1 values. The G+K network is unscored, while the remaining are scored using affinity scoring schemes.

**Table 15:** Overall relative ranking of complex detection algorithms on unscored and affinity-scored networks

Category	Method	Relative score	Normalized score
Unscored	CMC	3.000	1.000
	HACO	2.355	0.785
	MCL-CAw	2.130	0.710
	CORE	2.020	0.673
	MCL	1.879	0.626
	MCL-CA	1.960	0.653
	COACH	1.361	0.454

Scored	MCL-CAw	3.745	1.000
	HACO	3.733	0.997
	CMC	3.628	0.969
	MCLO	3.555	0.949
	MCL	3.468	0.926

Ranking obtained from normalized F1 values.

The precision of MCL-CAw (0.397) was lower on Bootstrap_0.094 _compared to other scored networks (ICD - 0.620, FSW - 0.615, Consolidated_3.19 _- 0.672). MCL-CAw produced many redundant complexes from this network compared to other scored networks, leading to the drop in precision. In fact we observed such variance in CMC and HACO algorithms as well. For example, CMC achieved the best recall on the ICD network, but lowest on the Consolidated network. Also, CMC produced significantly fewer complexes (#77) on the Consolidated network compared to other networks (ICD - 171, FSW - 179, Bootstrap - 203). Further, all algorithms displayed "sudden dips" in precision *versus *recall curves towards the beginning on the Bootstrap_0.094 _network (see Figure 8). All these findings indicate that the choice of affinity scoring schemes affected the performance of algorithms. In other words, each algorithm made use of certain characteristics of the PPI networks, and favored a scoring scheme that magnified or reinforced those characteristics. There was no single algorithm which performed relatively best on all the scored networks. Having said that, we note MCL-CAw was ranked among the top three algorithms on all scored networks, and therefore MCL-CAw responded reasonably well to the considered affinity scoring schemes.

We also ranked the different affinity-scored networks based on the F1 measures offered to the complex detection algorithms, as shown in Tables 16 and 17. The table shows that the Consolidated_3.19 _network offered the best F1 measures to the algorithms, followed by the FSW(Gavin+Krogan), ICD(Gavin+Krogan) and Bootstrap_0.094 _networks (the detailed calculations are presented in Additional files 2, Table S8). This agreed well with the fact that the Consolidated_3.19 _network was shown to have a TP/FP ratio comparable to small-scale experiments from MIPS, and therefore was of very high quality [11].

**Table 16:** Relative ranking of affinity scoring schemes for complex detection

PPI network		Wodak		MIPS		Aloy			
	Method	F1	Norm	F1	Norm	F1	Norm	Total	Norm
MCL	Cons_3.19_	0.573	1.000	0.407	1.000	0.567	0.970	2.970	1.000
	FSW(G+K)	0.541	0.944	0.393	0.965	0.585	1.000	2.909	0.980
	ICD(G+K)	0.498	0.871	0.370	0.908	0.543	0.928	2.706	0.911
	Boot_0.094_	0.426	0.744	0.299	0.733	0.400	0.684	2.161	0.728

MCLO	Cons_3.19_	0.606	1.000	0.471	1.000	0.575	0.939	2.939	1.000
	ICD(G+K)	0.533	0.879	0.412	0.875	0.572	0.934	2.688	0.914
	FSW(G+K)	0.513	0.847	0.376	0.798	0.612	1.000	2.645	0.900
	Boot_0.094_	0.424	0.700	0.267	0.567	0.392	0.641	1.908	0.649

MCL-CAw	Cons_3.19_	0.614	1.000	0.487	1.000	0.576	0.921	2.921	1.000
	FSW(G+K)	0.576	0.938	0.423	0.868	0.625	1.000	2.806	0.961
	ICD(G+K)	0.567	0.923	0.450	0.923	0.578	0.925	2.771	0.949
	Boot_0.094_	0.447	0.728	0.282	0.579	0.416	0.666	1.973	0.675

CMC	Cons_3.19_	0.604	1.000	0.479	1.000	0.588	1.000	3.000	1.000
	Boot_0.094_	0.577	0.955	0.366	0.764	0.515	0.877	2.597	0.866
	ICD(G+K)	0.531	0.880	0.403	0.843	0.480	0.816	2.538	0.846
	FSW(G+K)	0.484	0.801	0.338	0.705	0.465	0.791	2.297	0.766

HACO	FSW(G+K)	0.581	1.000	0.396	1.000	0.609	1.000	3.000	1.000
	Boot_0.094_	0.572	0.984	0.380	0.961	0.585	0.961	2.906	0.969
	ICD(G+K)	0.565	0.972	0.378	0.956	0.593	0.973	2.902	0.967
	Cons_3.19_	0.495	0.852	0.379	0.957	0.502	0.824	2.634	0.878

Ranking obtained from normalized F1 values.

**Table 17:** Overall relative ranking of affinity scoring schemes for complex detection

Scored network	Relative score	Normalized score
Cons_3.19_	4.878	1.000
FSW(G+K)	4.606	0.944
ICD(G+K)	4.588	0.941
Boot_0.094_	3.886	0.797

Ranking obtained from normalized F1 values.

### Impact of augmenting physical PPI networks with computationally inferred interactions

In this set of experiments, we studied whether augmenting the physical PPI networks with inferred interactions improved the performance of complex detection algorithms. We gathered interactions in yeast comprising of inferred interlogs (inferred from interactions between orthologous proteins in other organisms like fly, mouse and human), and also based on genetic (gene fusion, chromosomal proximity, gene co-evolution) and functional (traits of neighbors, neighbors of neighbors, etc.) associations; downloaded from the Predictome database [40]http://cagt.bu.edu/page/Predictome_about. These were used to generate the Inferred network (Table 1). We then augmented the Gavin+Krogan network with these interactions to generate the Gavin+Krogan+Inferred network and its scored versions, the ICD(Gavin+Krogan+Inferred) and FSW(Gavin+Krogan+Inferred) networks (Table 1).

We evaluated MCL, MCL-CAw, CMC and HACO on these augmented networks (Table 18). All the algorithms displayed very low precision and recall values on the Inferred network, indicating that the inferred interactions alone were not sufficient to predict meaningful complexes. Interestingly, most algorithms displayed marginal dip in their performance on Gavin+Krogan+Inferred compared to Gavin+Krogan. This dip in performance was explained by the analysis on the two augmented-scored networks, ICD(Gavin+Krogan+Inferred) and FSW(Gavin+Krogan+Inferred). Most algorithms showed higher precision and recall on these two augmented-scored networks compared to Gavin+Krogan and Gavin+Krogan+Inferred. This indicates that augmenting with raw inferred interactions gave little benefit due to presence of false positives (noise), but scoring the augmented networks helped to improve the precision and recall values of the algorithms.

**Table 18:** Impact of augmenting inferred interactions on the performance of MCL, MCL-CAw, CMC and HACO

Method	PPI Network	#Predicted complexes	#Matched predictions	Precision	#Derivable benchmarks	#Derived benchmarks	Recall
MCL	G+K	242	55	0.226	182	62	0.338
	I	50	2	0.040	31	3	0.097
	G+K+I	249	55	0.221	189	58	0.307
	ICD(G+K+I)	115	53	0.461	156	58	0.372
	FSW(G+K+I)	89	54	0.607	141	61	0.433

MCL-Caw	G+K	310	77	0.248	182	77	0.423
	I	42	2	0.048	31	3	0.097
	G+K+I	315	78	0.247	189	78	0.412
	ICD(G+K+I)	118	82	0.694	156	82	0.525
	FSW(G+K+I)	95	84	0.884	141	84	0.596

CMC	G+K	113	60	0.531	182	60	0.330
	I	10	3	0.300	31	5	0.161
	G+K+I	119	60	0.504	189	63	0.333
	ICD(G+K+I)	184	77	0.418	156	83	0.532
	FSW(G+K+I)	186	74	0.398	141	80	0.567

HACO	G+K	278	78	0.281	182	85	0.467
	I	12	2	0.167	31	2	0.064
	G+K+I	309	78	0.252	189	84	0.444
	ICD(G+K+I)	119	66	0.589	156	75	0.481
	FSW(G+K+I)	98	61	0.622	141	70	0.496

Most algorithms showed marginal dip in performance on Gavin+Krogan+Inferred compared to Gavin+Krogan. However, upon scoring the augmented network, their performance was better compared to Gavin+Krogan. This indicated that inferred interactions were useful for complex detection provided affinity scoring is employed to reduce the impact of the noise present in them.

### In-depth analysis of individual predicted complexes

To facilitate the analysis of our individual predicted complexes, we mapped the complexes back to the corresponding PPI networks and examined the interactions between components of the same complex, as well as between components of a given complex and other proteins in the network. We performed this analysis using the Cytoscape visualization environment http://www.cytoscape.org/[41].

#### Instances of correctly predicted complexes of MCL-CAw

The first example is of an attachment protein shared between two predicted complexes of MCL-CAw. The subunits of these predicted complexes (Id# 57 and 22) make up the Compass complex involved in telomeric silencing of gene expression [42], and the mRNA cleavage and polyadenylation specificity factor, a complex involved in RNAP II transcription termination [43]. The shared attachment Swd2 (Ykl018w) formed high confidence connections with the subunits of both predicted complexes. On this basis, the post-processing procedure assigned Swd2 (Ykl018w) to both predicted complexes, in agreement with available evidence [44] that Swd2 (Ykl018w) belongs to both Compass and mRNA cleavage complexes. The next example illustrates the case where a new protein was predicted as a subunit of a known complex. The attachment protein Ski7 (Yor076c) was included into a predicted complex (Id# 28) that matched the Exosome complex involved in RNA processing and degradation [45]. Additionally, Ski7 (Yor076c) was also included into a prediction (Id# 105) matching the Ski complex (Additional files 1, Figure S2). However, the Ski complex in the Wodak lab catalogue [29] did not include this new protein. Further literature survey suggested that Ski7 acts as a mediator between the Ski and Exosome complexes for 3'-to-5' mRNA decay in yeast [46].

The RNA polymerase I, II, and III complexes (also called Pol I, II, and III, respectively) are required for the generation of RNA chains [47]. As per the Wodak lab catalogue [29], all the three complexes share subunits: Yor224c, Ybr154c, Yor210w and Ypr187w, while Pol I and Pol III share Ynl113w and Ypr110c. Due to the extensive sharing of subunits, the corresponding predictions were grouped together into one large cluster by MCL. On the other hand, MCL-CAw segregated the large cluster into three independent complexes, which matched the Pol I, Pol II and Pol III complexes with accuracies of 0.714, 0.734 and 0.824, respectively.

In addition to these cases, a good fraction of already known core-attachment structures (reported in the supplementary materials of Gavin *et al. *[6]) were confirmed, and putative complexes were identified (preparation of a compendium currently in progress). Some examples are worth quoting here. Our predicted complex id# 44 closely matched the HOPS complex. All five cores {Ylr148w, Ylr396c, Ymr231w, Ypl045w, Yal002w} and two attachments {Ydr080w, Ydl077c} that were covered matched those reported in Gavin *et al. *Biological experiments show that the cores have the function of vacuole protein sorting, and with the help of attachments, the complex can perform homotypic vacuole fusion [48]. We identified the ubiquitin ligase ERAD-L complex comprising of Yos9(Ydr057w), Hrd3 (Ylr207w), Usa1 (Yml029w) and Hrd1 (Yol013c) that is involved in the degradation of ER proteins [49]. This matched the Hrd1/Hrd3 purified by Gavin *et al. *Four subunits {Oca4, Oca5, Siw14, Oca1} of a predicted novel complex (Id# 66) showed high similarity in functions (oxidant-induced cell-cycle arrest) and localization (cytoplasmic) when verified in SGD [33]. This complex exactly matched the putative complex 490 in Gavin *et al*.

#### Instances depicting mistakes in the predictions of MCL-CAw

Here we discuss an interesting case in which the sharing of subunits was so extensive and the web of interactions was so dense that separating out the smaller subsumed complexes purely on the basis of the interaction information was much harder. It was the amalgamation of the clusters matching the SAGA, SAGA-like (SLIK), ADA and TFIID complexes. Based on the Wodak lab catalogue [29], the 20 subunits making up the SAGA complex involved in transcriptional regulation [50] include four subunits (Ygr252w, Ydr176w, Ydr448w, Ypl254w) that are members of the ADA complex [51] as well. Sixteen components of the SAGA complex including the four shared with the ADA complex, are also the components of the SLIK complex [52]. Additionally, five subunits (Ybr198c, Ygl112c, Ymr236w, Ydr167w, Ydr145w) of the SAGA complex also belong to the TFIID complex [50]. Because of such extensive sharing of subunits involved in a dense web of interactions (436 interactions among 31 constituent proteins, as seen on the ICD(Gavin+Krogan) network), MCL-CAw was able to segregate out only two distinct complexes - SAGA (0.708) and SLIK (0.625). The clusters matching TFIID and ADA remained amalgamated together. In the next set of analysis, we compared the derived complexes from the Gavin+Krogan and the ICD(Gavin+Krogan) networks, and identified cases where MCL-CAw had missed a few proteins or whole complexes due to affinity scoring. From the Wodak, MIPS and Aloy reference sets, there were 13, 18 and 16 complexes, respectively, that were derived with better accuracies from the Gavin+Krogan network than from the ICD(Gavin+Krogan) network. And, there were 6, 2 and 2 complexes, respectively, that were derived from the Gavin+Krogan network, but missed totally from the ICD(Gavin+Krogan) network. Table 19 shows a sample of such complexes from the Wodak reference set. For the complexes that were derived with lower accuracies (upper half of Table 19), MCL-CAw had missed a few proteins due to low scores assigned to the corresponding interactions. For example, in the predicted complex from the ICD(Gavin+Krogan) network matching the SWI/SNF complex, two proteins (Ymr033w and Ypr034w) out of the four missed ones were absent due to their weak connections with the rest of the members; instead, these proteins were present in the prediction matching the RSC complex. In the Gavin+Krogan network, these two proteins were shared between two complexes matching the SWI/SNF and RSC complexes, which also agreed with the Wodak catalogue [29].

**Table 19:** Complexes derived with lesser accuracy or missed by MCL-CAw due to affinity scoring

*Matched benchmark complex*		*#Proteins in complexes from*		*#Incorrect proteins in complexes from ICD(G+K)*		*Accuracies (Jaccard scores)*	
Name	#Proteins	G+K	ICD(G+K)	Missed	Addnl	G+K	ICD(G+K)
Kornbergs SRB	25	24	23	2	0	0.960	0.920
SWI/SNF	12	11	8	4	0	0.769	0.667
TRAPP	10	10	9	1	0	1.000	0.900
19/22 S reg	22	20	27	0	5	0.909	0.815

TRAMP	3	4	7	0	4	0.750	0.429
Alpha-1,6	5	9	11	0	6	0.556	0.455
eIF3	7	8	14	1	8	0.500	0.400
Protein phosp	3	5	9	0	4	0.600	0.333
Cdc73p/Paf1p	7	7	18	0	11	0.556	0.388
Chs5p/Arf-1	6	8	10	2	6	0.556	0.400

The upper half shows sample complexes from Wodak lab derived with lower accuracies from the ICD(Gavin+Krogan) network compared to those from the Gavin+Krogan network. The lower half shows those missed from the ICD(Gavin+Krogan) network. The #Incorrect proteins in ICD(Gavin+Krogan) network is with respect to the benchmark complexes.

**Figure 9:**
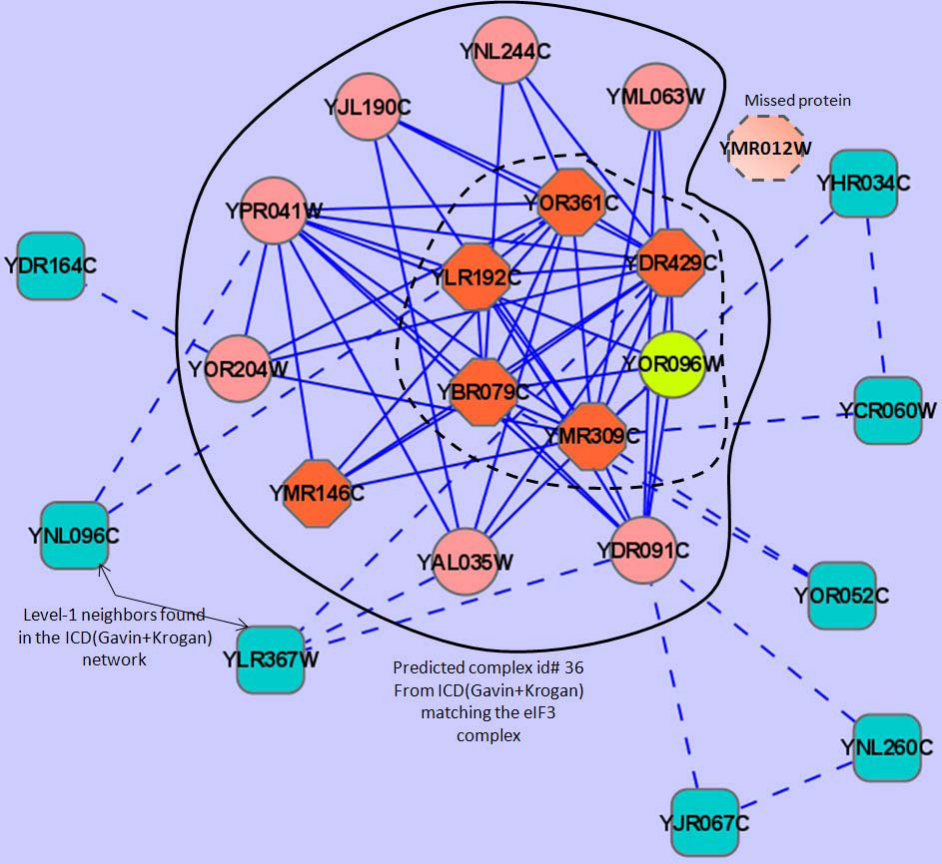
**Example of a complex missed by MCL-CAw from the ICD(Gavin+Krogan) network, but found from the Gavin+Krogan network**. The eIF3 complex from Wodak lab consisted of 7 proteins: Yor361c, Ylr192c, Ybr079c, Ymr309c, Ydr429c, Ymr012w and Ymr146c. The predicted complex id#36 from the ICD(Gavin+Krogan) network consisted of 14 proteins: 6 cores (Yor361c, Ylr192c, Ybr079c, Ymr309c, Ydr429c, Yor096w) and 8 attachments (Yal035w, Ydr091c, Yjl190c, Yml063w, Ymr146c, Ynl244c, Yor204w, Ypr041w). Therefore, there were 1 missed and 8 additional proteins in the prediction, leading to a low accuracy of 0.4. Hexagonal (Orange): eIF3 complex from Wodak lab. Circle (Orange, Yellow and Pink): Predicted complex id#36. Rectangle (Turquoise): Level-1 neighbors to the predicted complex id#36.

In the cases where MCL-CAw had completely missed some complexes from the scored network (lower half of Table 19), it is interesting to note that MCL-CAw had pulled-in many additional (noisy) proteins as attachments into the predicted complexes, which caused the accuracies to drop below 0.5. One such case is of the predicted complex id#36 matching the eIF3 complex with a low Jaccard score of 0.4. The eIF3 complex from Wodak lab consisted of 7 proteins: Yor361c, Ylr192c, Ybr079c, Ymr309c, Ydr429c, Ymr012w and Ymr146c. The predicted complex id#66 from the Gavin+Krogan network consisted of 8 proteins (Figure 9): 5 cores (Yor361c, Ylr192c, Ybr079c, Ymr309c, Ydr429c) and 3 attachments (Yor096w, Yal035w, Ydr091c). Therefore, there were 2 missed and 3 additional proteins in the prediction, leading to an accuracy of 0.5. The predicted complex id#36 from the ICD(Gavin+Krogan) network consisted of 14 proteins: 6 cores (Yor361c, Ylr192c, Ybr079c, Ymr309c, Ydr429c, Yor096w) and 8 attachments (Yal035w, Ydr091c, Yjl190c, Yml063w, Ymr146c, Ynl244c, Yor204w, Ypr041w). Therefore, there were 1 missed and 8 additional proteins in the prediction, leading to an even lower accuracy of 0.4. All the core proteins had same or similar GO annotations (involvement in translation, localized in cytoplasm or ribosomal subunit) [34]. Upon analysing the GO annotations of the 8 attachment proteins, we noticed that only one (Ymr146c) had the *same *annotation as the core proteins. This was also part of the eIF3 complex from Wodak lab [29]. Out of the remaining 7 attachment proteins, five (Ypr041w, Ynl244c, Yml063w, Yjl190c, Ydr091c) had *related *GO annotations (translation initiation, GTPase activity, cytoplasmic, ribosomal subunit) as the core proteins. A literature search revealed that these proteins belonged to the multi-eIF initiation factor conglomerate (containing eIF1, eIF2, eIF3 and eIF5) and the 40 S ribosomal subunit involved in translation [29]. The remaining two (Yal035w, Yor204w) were involved in translation activity, but were absent in the Wodak lab catalogue. These might be potentially new proteins belonging to the eIF3 or related complexes, and need to be further investigated. We also analysed the GO annotations of the level-1 neighbors to the predicted complex seen in the network, none of them had annotations similar to the proteins within the network. This example illustrates that carefully incorporating GO information into our algorithm to include or filter out proteins can be useful in cases where making decisions solely based on interaction information is difficult.

### Correlation between essentiality of proteins and their ability to form complexes

Early works by Jeong *et al. *[53] and Han *et al. *[54] studied the essentialities of proteins based on pairwise interactions within the interaction network, and concluded that hub (high-degree) proteins are more likely to be essential. This formed one of the criteria within the "centrality-lethality" rule [53]. However, a deeper insight can be obtained by studying the essentialities at cluster or group level of proteins rather than pairwise interactions. Recently, Zotenko *et al. *[55] argued that essential proteins often group together into densely connected sets of proteins performing essential functions, and thereby get involved in higher number of interactions resulting in their hubness property. Therefore, hubness may just an indirect indicator of protein essentiality. More recently, Kang *et al. *[56] studied essentiality of proteins by generating the reverse neighbor (RNN) topology [57] out of protein networks. This topology groups those proteins together that are within the reverse neighborhood of a given protein. Kang *et al. *concluded that centrality within the RNN topology is a better estimator of essentiality than hubness or degree in the interaction network. Studies by Hart *et al. *[12] showed that essential proteins are concentrated only in certain complexes, resulting in a dichotomy of essential and non-essential complexes. Wang *et al. *[21] concluded that the size of the (largest) recruiting complex of a protein may be a better indicator of protein essentiality than hubness.

In our work, we attempt to understand the relationship between the essentiality of proteins and their ability to form complexes. Table 20 shows that a high proportion (77.65%, 78.03%, 81.34% and 76.35% from the ICD(Gavin+Krogan), FSW (Gavin+Krogan), Consolidated_3.19 _and Bootstrap_0.094 _networks, respectively) of essential proteins present in the four affinity-scored networks belonged to at least some correctly predicted complex. This indicated that essential proteins are often members of complexes or co-clustered groups of proteins.

**Table 20:** Essential genes in the predicted complexes of MCL-CAw

	*Number (Proportion) of Essential genes present in*			
PPI Network	Whole network	Predicted cores	Predicted complexes	Matched predictions
ICD(Gavin+Krogan)	604 (0.537)	510 (0.454)	552 (0.491)	469 (0.417)
FSW(Gavin+Krogan)	604 (0.537)	510 (0.454)	552 (0.491)	470 (0.418)
Consolidated_3.19_	611 (0.544)	568 (0.506)	576 (0.513)	497 (0.442)
Bootstrap_0.094_	757 (0.674)	634 (0.564)	676 (0.601)	578 (0.514)

The figures in brackets represent the proportion of essential genes present in the corresponding group out of the 1123 total essential genes obtained from the Yeast Genome Deletion project [36,37].

# Essential genes in YDP: 1123

**Figure 10:**
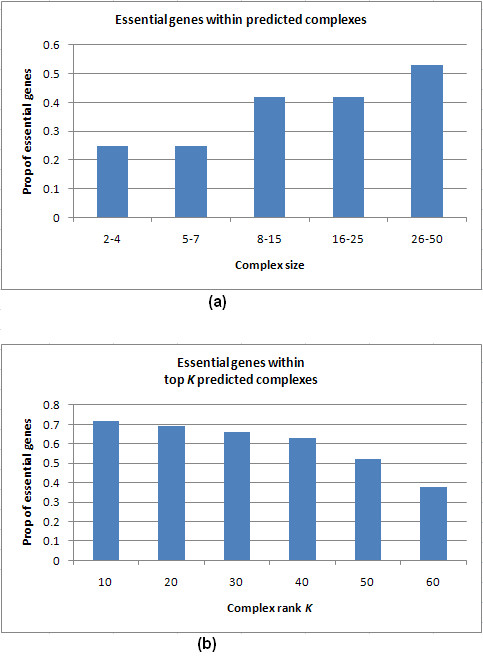
**Correlation between essentiality of proteins and their abilities to form complexes**. (a): Proportion of essential proteins within complexes of different sizes, predicted from ICD(Gavin+Krogan). Proportion of essential proteins in a complex = #essential proteins/total #proteins in the complex. (b): Proportion of essential proteins within top *K *ranked complexes.

To further analyse this ability of essential proteins to form complexes or groups, we binned our correctly predicted complexes based on their sizes and calculated the proportion of essential proteins in all complexes for each bin (like in [21]). Figure 10(a) shows that essential proteins were present in higher proportions within larger complexes. We then calculated the proportion of essential proteins within the top *K *ranked complexes. Figure 10(b) shows that essential proteins were present in higher proportions within higher ranked complexes. Both these figures hint at the same finding: essential proteins come together in large groups to perform essential functions.

## Discussion

In spite of the advances in computational approaches to derive complexes, high-accuracy reconstruction of complexes has still remained a challenging task. In deriving protein complexes from PPI networks, a key assumption made by most computational approaches is that complexes form densely connected regions within the networks. Therefore, these approaches attempt to cluster the networks based on measures related to connectivities between proteins in the network. Some approaches like MCL simulate random walks (called flow) to identify dense regions, while others like CMC merge maximal cliques into larger dense clusters. Therefore, the performance of these methods varies widely depending on network densities. A glance through Tables 8 to 12 reveals that all the methods considered for comparison in this work achieve very low recall on the MIPS set compared to the Wodak and Aloy sets. Table 2 shows that the average density of complexes in MIPS is much lower than that of Wodak and Aloy sets. Only 52 out of 137 (37.95%) derivable MIPS complexes of size ≥ 5 could be detected from the Gavin+Krogan network by all methods put together. We analysed the remaining 85 MIPS complexes and found most of them to have very low densities (average about 0.217) in the Gavin+Krogan network. For example, the MIPS complex 440.30.10 (involved in mRNA splicing) went undetected by all the methods even though 40 of its 42 proteins were present in the Gavin+Krogan network. There were 144 interactions among these 40 proteins, giving a low density of 0.184 to the complex in this network. Continuing with this analysis, we tested MCL and MCL-CAw on a PPI dataset from DIP http://dip.doe-mbi.ucla.edu, comprising of 17491 interactions among 4932 proteins giving a low average node degree of 7.092. MCL-CAw was able to achieve only marginal improvement (22.8% higher precision and 7.4% higher recall) over MCL, due to the low average node degree of the DIP network. These experiments show that all the methods considered here find it difficult to uncover complexes that are very sparse. This should prompt us to rethink whether over importance is being given to model complexes as dense regions in PPI networks.

Apart from these limitations in the existing computational methods, there are some inherent difficulties in the accumulation of interactome data as well that make complex detection difficult. Complexes display different lifetimes, and their compositions vary based on cellular localizations (compartments) and conditions. The same protein may be recruited by different complexes at different times and conditions. Due to such temporal and spatial variability of complexes, repeated purifications using TAP/MS methods yield somewhat different "complex forms" [20]. The PPI networks constructed out of such purifications represent only a probabilistic average picture of the yeast interactome [20]. Therefore, the complexes predicted out of such networks only approximate the actual complex compositions.

Another limitation arises from the bias in TAP/MS purifications against complexes of certain kind (for example, membrane-bound complexes). Since TAP/MS data are acquired in a single condition (rich media), some complexes may not be present in the cell in that condition [21]. Therefore, new experimental assays are needed before such complexes can be reconstructed and studied.

Finally, even though *S. cerevisiae *is used as a model organism for eukaryotic interactome analysis, some key complexes specialized to other organisms (including human) can be studied only by analysing the interaction datasets specific to these organisms. However, the incompleteness of interactome data from these organisms makes the reconstruction of complexes difficult.

## Conclusion

The ultimate goal of interactome analysis is to understand the higher level organization of the cell. Reconstruction of protein complexes serves as a building block towards achieving this goal. In this paper, inspired by the findings of Gavin *et al. *[6], we developed a novel core-attachment based refinement method coupled to MCL to identify yeast complexes from weighted PPI networks. We demonstrated that our algorithm (MCL-CAw) performed better than MCL in deriving meaningful yeast complexes particularly in the presence of natural noise. We also showed that MCL-CAw responded reasonably well to the considered affinity scoring schemes. In the future work, we intend to improve the prediction ability of our algorithm by incorporating information from gene annotations, gene expressions, literature mining as well as domain-domain interactions. We also intend to extend our work to predict complexes of organisms other than yeast. In this context, we intend to use our MCL-CAw model to study the existence (and extent) of core-attachment modularity in complexes from other organisms.

## Availability

The MCL-CAw software is developed using PL/SQL on Oracle 10 g, using the framework in [58]. The source code, yeast PPI datasets, benchmark and predicted yeast complexes used in this work are freely available at the MCL-CAw project homepage hosted on the NUS server: http://www.comp.nus.edu.sg/~leonghw/MCL-CAw/.

## Authors' contributions

SS conceived the initial ideas and discussed them with HWL and KN. SS devised the algorithm, developed the software, performed the experiments and analysis, and wrote and revised the manuscript. HWL supervised the project, advised SS, and reviewed and revised the manuscript. KN took part in the discussions and helped in reviewing the manuscript. All authors have read and approved the manuscript.

## Supplementary Material

Additional files 1 **Additional figures and tables**: Figures for core-attachment modularity and illustration of a predicted complex by MCL-CAw. Tables for setting of MCL-CAw parameters, and ranking of complex detection algorithms and affinity-scored networks. Click here for file

Additional files 2 **The MCL-CAw software package**: The source code and installation details for the MCL-CAw software. Click here for file
