# Supplementary material for: MCL-CAw: a refinement of MCL for detecting yeast complexes from weighted PPI networks by incorporating core-attachment structure
Source: BMC Bioinformatics. 2010 Oct 12;11:504. doi: 10.1186/1471-2105-11-504 (PMC2965181; doi:10.1186/1471-2105-11-504)
Supplement: Additional files 1 — Additional figures and tables: Figures for core-attachment modularity and illustration of a predicted complex by MCL-CAw. Tables for setting of MCL-CAw parameters, and ranking of complex detection algorithms and affinity-scored networks. [file 1471-2105-11-504-S1.PDF]

MCL-CAw: A refinement of MCL for detecting yeast complexes from weighted PPI networks by incorporating core-attachment structure

Sriganesh Srihari, Kang Ning, Hon Wai Leong

SS: srigsri@comp.nus.edu.sg; KN: kning@umich.edu; HWL: leonghw@comp.nus.edu.sg

Additional files 1

Figures

S1: Depicting of core-attachment modularity in protein complexes of yeast (Gavin et al., Nature, 2006)

S2: Sharing of YOR076C between the Exosome and Ski complexes

Tables

S1: Setting the inflation coefficient *I* for MCL (unscored Gavin+Krogan network)

S2: Setting the inflation coefficient *I* for MCL (scored ICD(Gavin+Krogan) network)

S3: Choosing α and γ for MCL-CAw (unscored Gavin+Krogan network)

S4: Choosing α and γ for MCL-CAw (scored ICD(Gavin+Krogan) network)

S5: Reconfirming α and γ for different inflation values *I* (unscored Gavin+Krogan network)

S6: Reconfirming α and γ for different inflation values *I* (scored ICD(Gavin+Krogan) network)

S7: Relative ranking of complex detection algorithms

S8: Relative ranking of affinity scored networks for complex detection

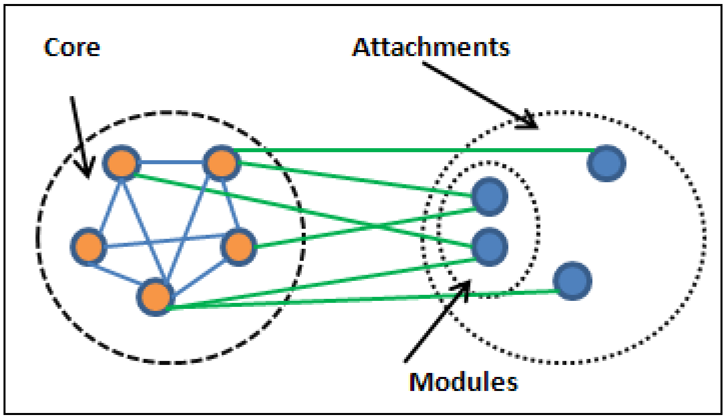

Figure S1

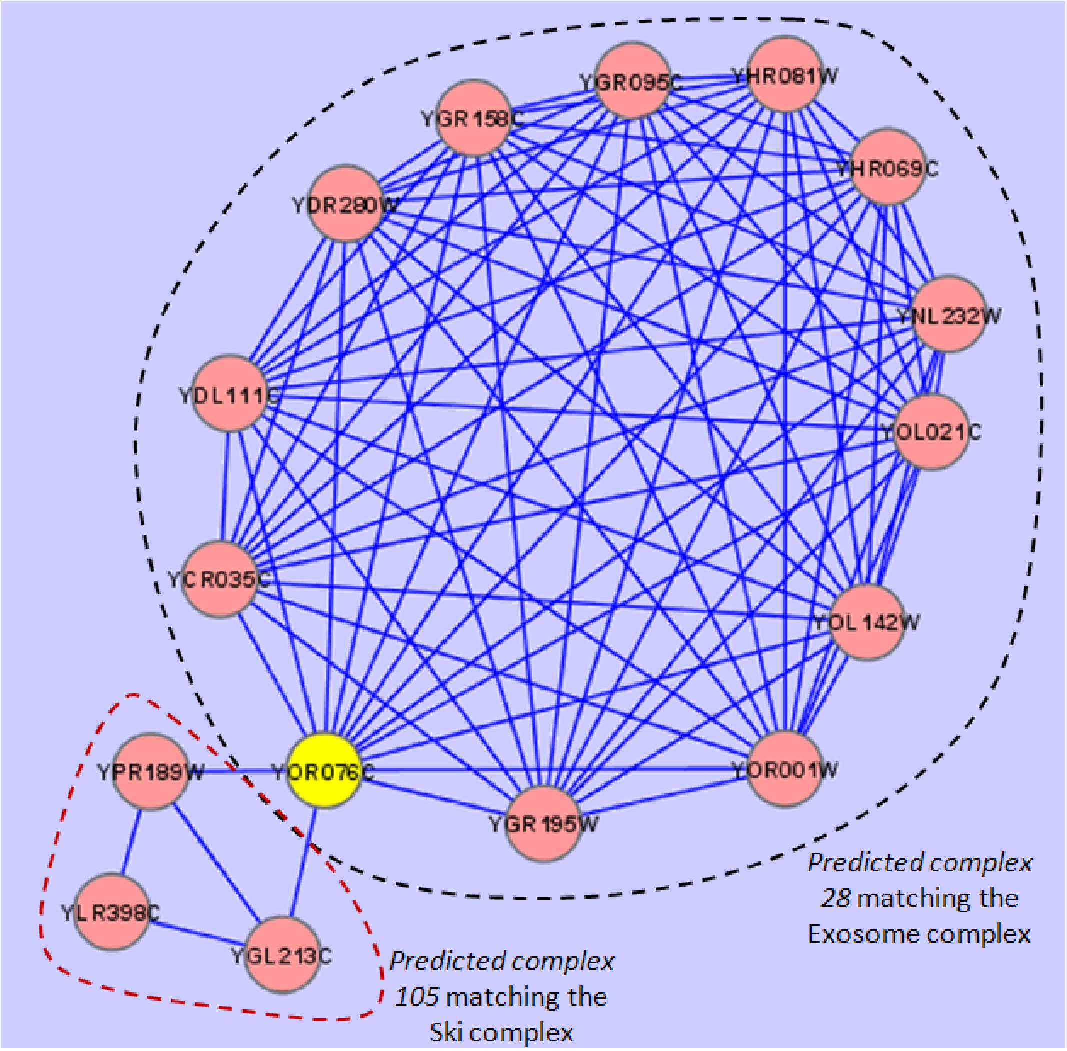

Figure S2

TABLE S1 Setting the inflation coefficient I for MCL

Unscored Gavin+Krogan network

Inflation *I*	#Predicted	Avg cluster size	#Predicted (s≥4)	Avg cluster size (s≥4)	WODAK(#182, 6.835) Precision	WODAK Recall	WODAK F1	MIPS (#177, 14.322) Precision	MIPS Recall	MIPS F1	ALOY (#76, 7.868) Precision	ALOY Recall	ALOY F1
1.250	128.000	23.125	72.000	39.375	0.167	0.071	0.100	0.111	0.051	0.070	0.069	0.066	0.067
1.500	318.000	9.320	205.000	13.107	0.205	0.253	0.226	0.132	0.192	0.156	0.161	0.434	0.235
1.750	507.000	5.846	256.000	9.359	0.215	0.335	0.262	0.137	0.226	0.171	0.168	0.539	0.256
2.000	638.000	4.645	245.000	8.523	0.222	0.336	0.267	0.143	0.226	0.175	0.175	0.553	0.266
2.250	804.000	3.636	244.000	7.590	0.226	0.336	0.270	0.143	0.226	0.175	0.176	0.556	0.267
2.500	892.000	3.322	242.000	7.103	0.226	0.338	0.271	0.143	0.226	0.175	0.179	0.556	0.271
2.750	974.000	3.043	231.000	6.844	0.220	0.338	0.267	0.143	0.203	0.168	0.180	0.553	0.272
3.000	1038.000	2.855	222.000	6.648	0.220	0.336	0.266	0.144	0.198	0.167	0.176	0.513	0.262
						**MAX**	**0.271**			**0.175**			**0.272**

Inflation *I*	WODAK F1	MIPS F1	ALOY F1	Total F1	Normalized
1.250	0.368	0.399	0.248	1.015	0.339
1.500	0.836	0.893	0.865	2.594	0.866
1.750	0.967	0.974	0.943	2.884	0.962
2.000	0.987	1.000	0.979	2.966	0.990
2.250	0.998	1.000	0.984	2.982	0.995
**2.500**	**1.000**	**1.000**	**0.997**	**2.997**	**1.000**
2.750	0.984	0.958	1.000	2.942	0.982
3.000	0.982	0.952	0.965	2.898	0.967
			**MAX**	**2.997**	

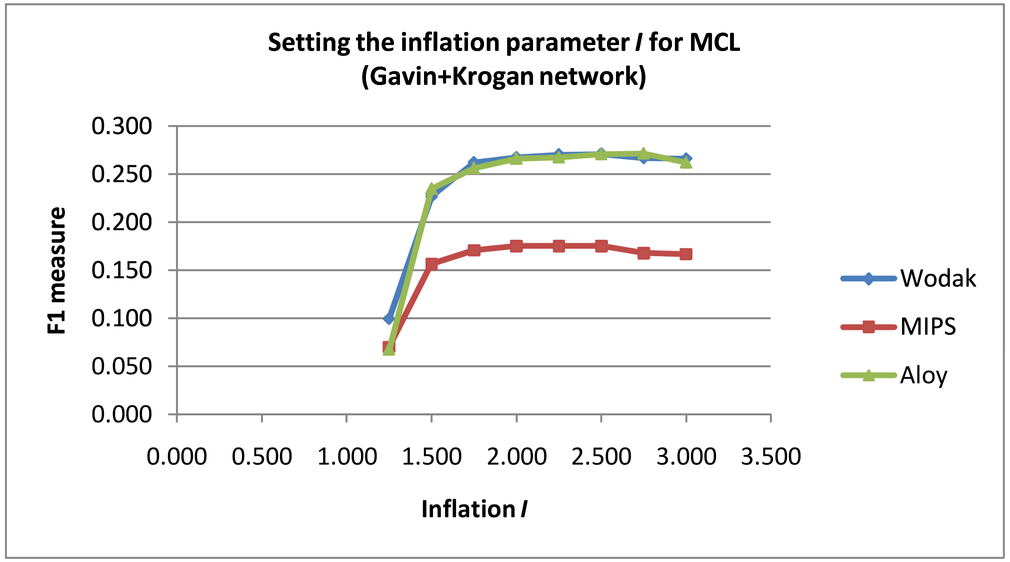

TABLE S2 **Setting the inflation coefficient I for MCL**

ICD(Gavin+Krogan) network

Inflation *I*	#Predicted	Avg cluster size	#Predicted (s≥4)	Avg cluster size (s≥4)	WODAK(#153, 7.00) Precision	Recall	F1	MIPS (#151, 13.55) Precision	Recall	F1	ALOY (#75, 7.60) Precision	Recall	F1
1.250	89.000	18.292	69.000	22.724	0.507	0.255	0.339	0.377	0.218	0.276	0.478	0.440	0.458
1.500	126.000	12.920	103.000	15.135	0.544	0.398	0.460	0.340	0.292	0.314	0.466	0.640	0.539
1.750	141.000	11.546	113.000	13.672	0.531	0.438	0.480	0.336	0.312	0.323	0.469	0.706	0.564
2.000	154.000	10.571	121.000	12.644	0.512	0.451	0.479	0.339	0.331	0.335	0.438	0.706	0.541
2.250	166.000	9.807	129.000	11.775	0.488	0.458	0.473	0.326	0.338	0.332	0.426	0.734	0.539
2.500	175.000	9.302	136.000	11.139	0.500	0.497	0.498	0.346	0.411	0.376	0.426	0.773	0.549
2.750	185.000	8.800	144.000	10.490	0.486	0.501	0.493	0.326	0.411	0.364	0.403	0.773	0.530
3.000	198.000	8.222	147.000	10.129	0.483	0.501	0.492	0.320	0.411	0.360	0.408	0.773	0.534

MAX **0.498** **0.376** **0.564**

Inflation *I*	Wodak	MIPS	Aloy	Total F1	Normalized
1.250	0.680	0.735	0.813	2.228	0.749
1.500	0.923	0.836	0.957	2.716	0.913
1.750	0.963	0.861	1.000	2.824	0.949
2.000	0.962	0.891	0.959	2.812	0.945
2.250	0.948	0.883	0.956	2.787	0.937
**2.500**	**1.000**	**1.000**	**0.974**	**2.974**	**1.000**
2.750	0.990	0.968	0.940	2.897	0.974
3.000	0.987	0.958	0.947	2.892	0.972

2.974

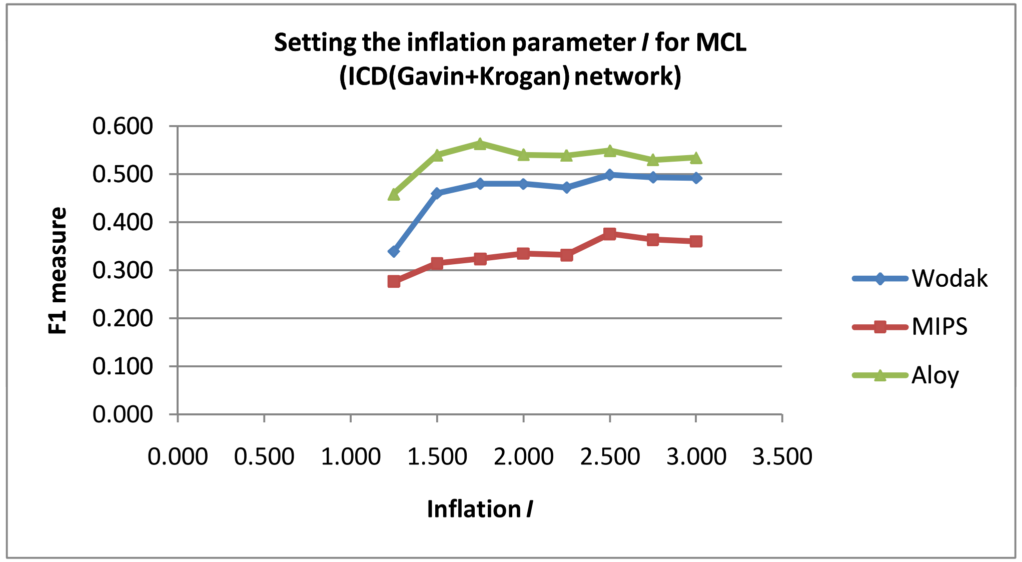

TABLE S3 **Choosing alpha and gamma for MCL-CAw**

Unscored Gavin+Krogan network

$$I(p,Core(C)) \geq \alpha . I(Core(C)) . (|Core(C)|/2)^{(-\gamma)}$$

I=2.5 **α=1.0**

γ	#Clusters (s>4)	Avg cluster size	WODAK (#182, 6.835) Avg Jaccard (Whole)	Avg Jaccard (Attach)	Final precision (threshold t = 0.5)	Final recall	F1	MIPS (#177, 14.322) Avg Jaccard (Whole)	Avg Jaccard (Attach)	Final precision (threshold t = 0.5)	Final recall	F1	ALOY (#76, 7.868) Avg Jaccard (Whole)	Avg Jaccard (Attach)	Final precision (threshold t = 0.5)	Final recall	F1
0.150	244.000	7.963	0.577	0.176	0.258	0.385	0.309	0.487	0.079	0.165	0.293	0.211	0.632	0.154	0.199	0.624	0.302
0.250	244.000	7.968	0.579	0.178	0.262	0.396	0.315	0.489	0.083	0.165	0.293	0.211	0.635	0.155	0.201	0.632	0.305
0.500	244.000	7.971	0.579	0.178	0.262	0.396	0.315	0.489	0.083	0.168	0.294	0.214	0.635	0.155	0.201	0.632	0.305
0.750	244.000	7.987	0.579	0.179	0.262	0.396	0.315	0.489	0.083	0.168	0.294	0.214	0.635	0.155	0.201	0.632	0.305
1.000	244.000	8.201	0.576	0.228	0.258	0.385	0.309	0.498	0.133	0.168	0.294	0.214	0.641	0.199	0.201	0.632	0.305
1.250	244.000	8.368	0.576	0.231	0.254	0.379	0.304	0.499	0.133	0.168	0.294	0.214	0.637	0.192	0.197	0.618	0.299
1.500	244.000	8.979	0.579	0.247	0.254	0.385	0.306	0.494	0.148	0.156	0.271	0.198	0.637	0.233	0.189	0.592	0.287

I=2.5 **γ=0.75**

α	#Clusters (s>4)	Avg cluster size	WODAK (#182, 6.835) Avg Jaccard (Whole)	Avg Jaccard (Attach)	Final precision	Final recall	F1	MIPS (#177, 14.322) Avg Jaccard (Whole)	Avg Jaccard (Attach)	Final precision	Final recall	F1	ALOY (#76, 7.868) Avg Jaccard (Whole)	Avg Jaccard (Attach)	Final precision	Final recall	F1
0.500	283.000	8.964	0.575	0.235	0.216	0.374	0.274	0.497	0.133	0.138	0.271	0.183	0.636	0.265	0.163	0.592	0.256
**0.750**	**244.000**	**8.262**	**0.577**	**0.227**	**0.258**	**0.385**	0.309	**0.500**	**0.133**	**0.168**	**0.294**	0.214	**0.645**	**0.199**	**0.201**	**0.632**	0.305
**1.000**	**244.000**	**8.201**	**0.576**	**0.228**	**0.258**	**0.385**	0.309	**0.498**	**0.133**	**0.168**	**0.294**	0.214	**0.641**	**0.199**	**0.201**	**0.632**	0.305
**1.250**	**157.000**	**8.401**	**0.597**	**0.144**	**0.389**	**0.385**	0.387	**0.494**	**0.063**	**0.248**	**0.282**	0.264	**0.651**	**0.086**	**0.299**	**0.618**	0.403
1.500	153.000	8.477	0.601	0.134	0.392	0.379	0.385	0.496	0.059	0.255	0.282	0.268	0.651	0.086	0.307	0.618	0.410
1.750	149.000	8.349	0.609	0.131	0.392	0.372	0.382	0.496	0.058	0.254	0.282	0.267	0.651	0.082	0.306	0.612	0.408

α = 1.50 γ = 0.75

γ	F1 Wodak	F1 MIPS	F1 Aloy	Norm F1 Wodak	Norm F1 MIPS	Norm F1 Aloy	Total	Norm
0.150	0.3090	0.2111	0.3018	0.97971	0.98735	0.98939	2.95646	0.98549
0.250	0.3154	0.2111	0.3050	1.00000	0.98735	1.00000	2.98735	0.99578
**0.500**	**0.3154**	**0.2138**	**0.3050**	**1.00000**	**1.00000**	**1.00000**	**3.00000**	**1.00000**
**0.750**	**0.3154**	**0.2138**	**0.3050**	**1.00000**	**1.00000**	**1.00000**	**3.00000**	**1.00000**
1.000	0.3090	0.2138	0.3050	0.97971	1.00000	1.00000	2.97971	0.99324
1.250	0.3042	0.2138	0.2988	0.96449	1.00000	0.97956	2.94405	0.98135
1.500	0.3061	0.1980	0.2865	0.97056	0.92609	0.93943	2.83608	0.94536
**MAX**	0.3154	0.2138	0.3050				3.00000	

α	F1 Wodak	F1 MIPS	F1 Aloy	Norm F1 Wodak	Norm F1 MIPS	Norm F1 Aloy	Total	Norm
0.500	0.274	0.183	0.256	0.708	0.683	0.623	2.014	0.672
0.750	0.309	0.214	0.305	0.798	0.798	0.744	2.340	0.781
1.000	0.309	0.214	0.305	0.798	0.798	0.744	2.340	0.781
1.250	0.387	0.264	0.403	1.000	0.985	0.982	2.968	0.991
**1.500**	**0.385**	**0.268**	**0.410**	**0.996**	**1.000**	**1.000**	**2.996**	**1.000**
1.750	0.382	0.267	0.408	0.986	0.998	0.995	2.979	0.994
**MAX**	0.387	0.268	0.410				2.996	

TABLE S4 **Choosing alpha and gamma for MCL-CAw**

ICD(Gavin+Krogan) network

I(p,Core(C)) ≥ α.I(Core(C)).(|Core(C)|/2)^(-γ)

I=2.50 **α=0.50**

γ	#Clusters (s>4)	Avg cluster size	WODAK(#153, 7.00) Avg Jaccard (Whole)	WODAK Avg Jaccard (Attach)	WODAK Final precision (threshold t = 0.5)	WODAK Final recall	WODAK F1	MIPS (#151, 13.55) Avg Jaccard (Whole)	MIPS Avg Jaccard (Attach)	MIPS Final precision (threshold t = 0.5)	MIPS Final recall	MIPS F1	ALOY (#75, 7.60) Avg Jaccard (Whole)	ALOY Avg Jaccard (Attach)	ALOY Final precision (threshold t = 0.5)	ALOY Final recall	ALOY F1
0.250	127.000	9.291	0.679	0.057	0.559	0.523	0.540	0.564	0.033	0.394	0.450	0.420	0.741	0.018	0.472	0.800	0.594
0.500	128.000	9.257	0.680	0.057	0.563	0.529	0.545	0.564	0.033	0.391	0.450	0.418	0.741	0.018	0.469	0.800	0.591
0.750	128.000	9.281	0.680	0.057	0.563	0.529	0.545	0.564	0.033	0.391	0.450	0.418	0.741	0.018	0.469	0.800	0.591
1.000	130.000	9.323	0.674	0.068	0.554	0.529	0.541	0.560	0.025	0.377	0.444	0.408	0.738	0.018	0.462	0.800	0.586

I=2.50 **γ=0.75**

α	#Clusters (s>4)	Avg cluster size	WODAK(#153, 7.00) Avg Jaccard (Whole)	WODAK Avg Jaccard (Attach)	WODAK Final precision	WODAK Final recall	WODAK F1	MIPS (#151, 13.55) Avg Jaccard (Whole)	MIPS Avg Jaccard (Attach)	MIPS Final precision	MIPS Final recall	MIPS F1	ALOY (#75, 7.60) Avg Jaccard (Whole)	ALOY Avg Jaccard (Attach)	ALOY Final precision	ALOY Final recall	ALOY F1
0.250	131.000	9.343	0.678	0.056	0.555	0.520	0.537	0.563	0.033	0.390	0.450	0.418	0.741	0.018	0.467	0.792	0.588
0.500	128.000	9.281	0.680	0.057	0.563	0.529	0.545	0.564	0.033	0.391	0.450	0.418	0.741	0.018	0.469	0.800	0.591
0.750	126.000	9.317	0.683	0.059	0.563	0.523	0.542	0.564	0.033	0.397	0.450	0.422	0.741	0.018	0.476	0.800	0.597
1.000	125.000	9.296	0.678	0.074	0.560	0.516	0.537	0.565	0.038	0.392	0.444	0.416	0.741	0.018	0.480	0.800	0.600

α = 1.000 γ = 0.750

γ	F1 Wodak	F1 MIPS	F1 Aloy	Norm F1 Wodak	Norm F1 MIPS	Norm F1 Aloy	Total	Norm
0.250	0.540	0.420	0.594	0.991	1.000	1.000	2.991	0.999
**0.500**	**0.545**	**0.418**	**0.591**	**1.000**	**0.996**	**0.996**	**2.992**	**1.000**
**0.750**	**0.545**	**0.418**	**0.591**	**1.000**	**0.996**	**0.996**	**2.992**	**1.000**
1.000	0.541	0.408	0.586	0.992	0.971	0.987	2.949	0.985
**MAX**	0.545	0.420	0.594				2.992	

α	F1 Wodak	F1 MIPS	F1 Aloy	Norm F1 Wodak	Norm F1 MIPS	Norm F1 Aloy	Total	Norm
0.250	0.5369	0.3412	0.4172	0.984	0.488	0.687	2.159	0.724
0.500	0.5455	0.5217	0.5333	1.000	0.747	0.878	2.625	0.879
0.750	0.5423	0.6294	0.5826	0.994	0.901	0.959	2.854	0.956
**1.000**	**0.5371**	**0.6988**	**0.6074**	**0.985**	**1.000**	**1.000**	**2.985**	**1.000**
	0.5455	0.6988	0.6074				2.985	

TABLE S5 **Reconfirming α and γ for different values of inflation *I***

Unweighted Gavin+Krogan network $\alpha = 1.50$ $\gamma = 0.75$

					WODAK(#182, 6.835)						MIPS (#177, 14.322)						ALOY (#76, 7.868)					
	# Predicted		Avg cluster size		Precision		Recall		F1		Precision		Recall		F1		Precision		Recall		F1	
Inflation *I*	MCL	MCL-CAw	MCL	MCL-CAw	MCL	MCL-CAw	MCL	MCL-CAw	MCL	MCL-CAw	MCL	MCL-CAw	MCL	MCL-CAw	MCL	MCL-CAw	MCL	MCL-CAw	MCL	MCL-CAw	MCL	MCL-CAw
1.250	72	64	39.375	29.790	0.167	0.234	0.071	0.088	0.100	0.128	0.111	0.141	0.051	0.062	0.070	0.086	0.069	0.141	0.066	0.118	0.067	0.128
1.500	205	152	13.107	10.921	0.205	0.316	0.253	0.286	0.226	0.300	0.146	0.230	0.192	0.243	0.166	0.236	0.161	0.243	0.434	0.487	0.235	0.324
1.750	256	165	9.385	8.624	0.215	0.364	0.335	0.379	0.262	0.371	0.137	0.230	0.226	0.277	0.171	0.251	0.168	0.279	0.539	0.605	0.256	0.382
2.000	245	146	8.523	8.335	0.222	0.432	0.336	0.396	0.267	0.413	0.143	0.295	0.226	0.311	0.175	0.303	0.175	0.342	0.553	0.658	0.266	0.450
2.250	244	131	7.590	7.710	0.226	0.466	0.336	0.390	0.270	0.425	0.143	0.305	0.226	0.288	0.175	0.296	0.176	0.336	0.556	0.579	0.267	0.425
2.500	242	124	7.103	6.830	0.226	0.500	0.338	0.390	0.271	0.438	0.143	0.371	0.226	0.311	0.175	0.338	0.179	0.379	0.556	0.618	0.271	0.470
2.750	231	117	6.844	6.581	0.220	0.513	0.338	0.374	0.267	0.433	0.143	0.359	0.203	0.282	0.168	0.316	0.180	0.376	0.553	0.579	0.272	0.456
3.000	222	116	6.648	6.215	0.220	0.483	0.336	0.352	0.266	0.407	0.144	0.328	0.198	0.254	0.167	0.286	0.176	0.336	0.513	0.513	0.262	0.406

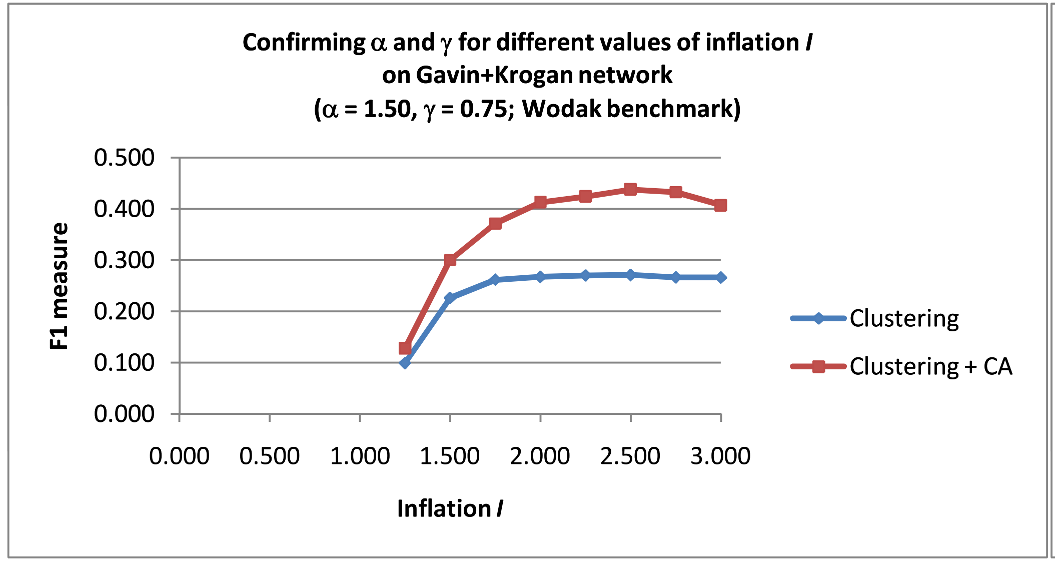

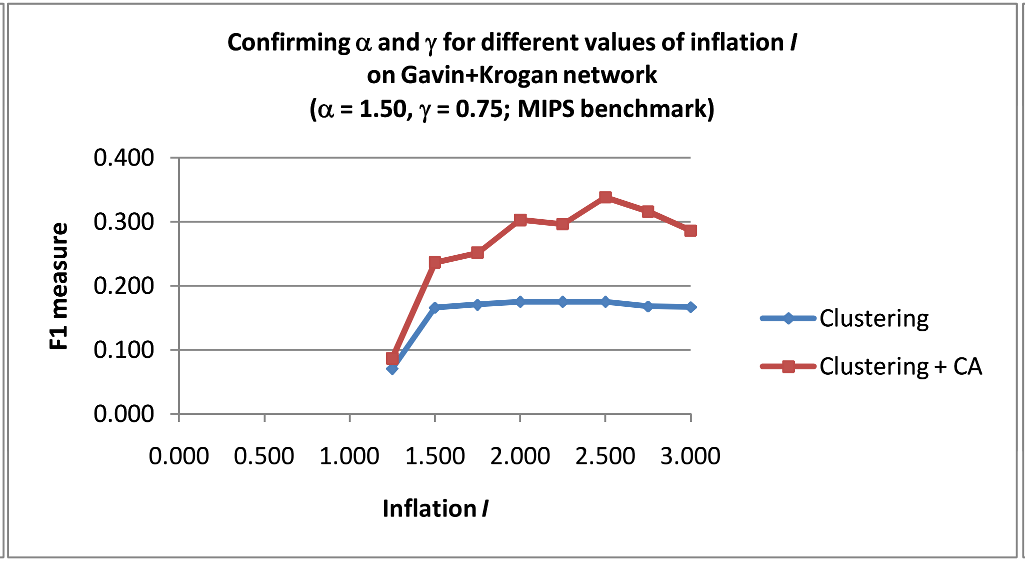

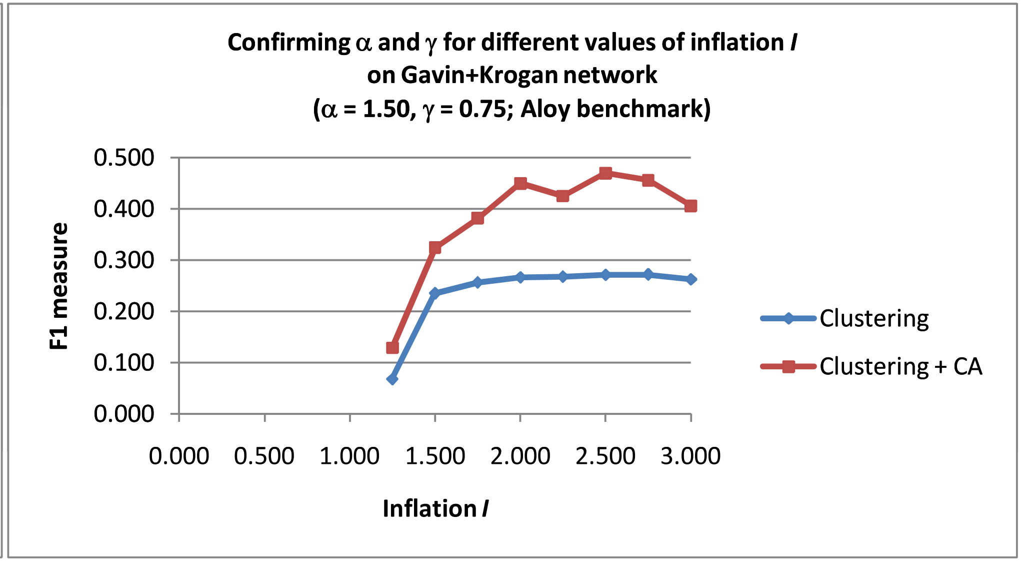

TABLE S6 **Reconfirming α and γ for different values of inflation *I***

ICD (Gavin+Krogan) network α = 1.00 γ = 0.75

					WODAK(#153, 7.00)						MIPS (#151, 13.55)						ALOY (#75, 7.60)					
	# Predicted		Avg cluster size		Precision		Recall		F1		Precision		Recall		F1		Precision		Recall		F1	
Inflation *I*	MCL	MCL-CAw	MCL	MCL-CAw	MCL	MCL-CAw	MCL	MCL-CAw	MCL	MCL-CAw	MCL	MCL-CAw	MCL	MCL-CAw	MCL	MCL-CAw	MCL	MCL-CAw	MCL	MCL-CAw	MCL	MCL-CAw
1.250	69.000	69	22.724	16.043	0.507	0.580	0.255	0.301	0.339	0.396	0.377	0.464	0.218	0.298	0.276	0.363	0.478	0.536	0.440	0.493	0.458	0.514
1.500	103.000	101	15.135	11.722	0.544	0.594	0.398	0.458	0.460	0.517	0.340	0.416	0.292	0.377	0.314	0.396	0.466	0.515	0.640	0.693	0.539	0.591
1.750	113.000	110	13.672	10.736	0.531	0.591	0.438	0.490	0.480	0.536	0.336	0.409	0.312	0.397	0.323	0.403	0.469	0.518	0.706	0.760	0.564	0.616
2.000	121.000	114	12.644	10.254	0.512	0.588	0.451	0.497	0.479	0.539	0.339	0.412	0.331	0.424	0.335	0.418	0.438	0.509	0.706	0.773	0.541	0.614
2.250	129.000	122	11.775	9.541	0.488	0.566	0.458	0.510	0.473	0.537	0.326	0.393	0.338	0.437	0.332	0.414	0.426	0.484	0.734	0.787	0.539	0.599
2.500	136.000	125	11.139	9.296	0.500	0.560	0.497	0.516	0.498	0.537	0.346	0.392	0.411	0.444	0.376	0.416	0.426	0.480	0.773	0.800	0.549	0.600
2.750	144.000	128	10.490	8.906	0.486	0.555	0.501	0.529	0.493	0.542	0.326	0.383	0.411	0.444	0.364	0.411	0.403	0.477	0.773	0.813	0.530	0.601
3.000	147.000	134	10.129	8.858	0.483	0.530	0.501	0.529	0.492	0.529	0.320	0.366	0.411	0.444	0.360	0.401	0.408	0.455	0.773	0.813	0.534	0.583

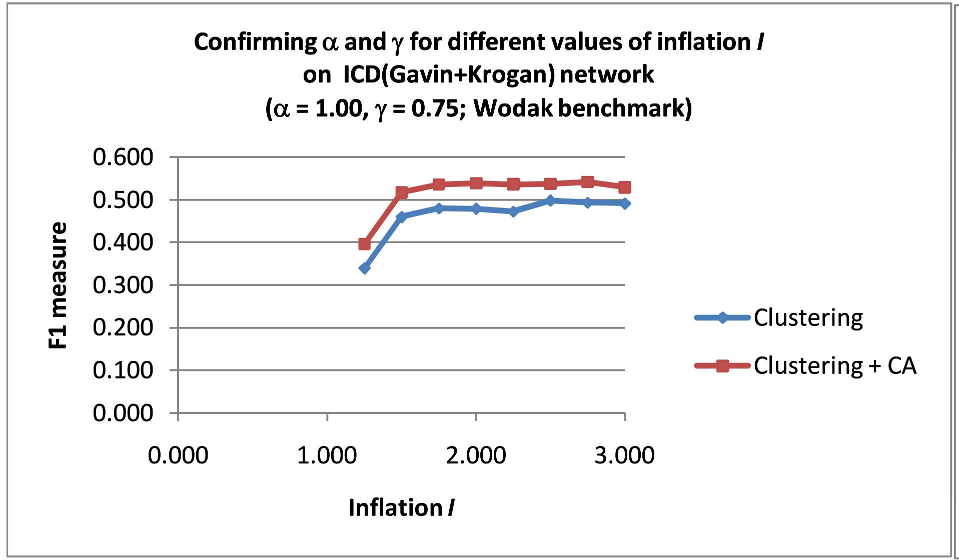

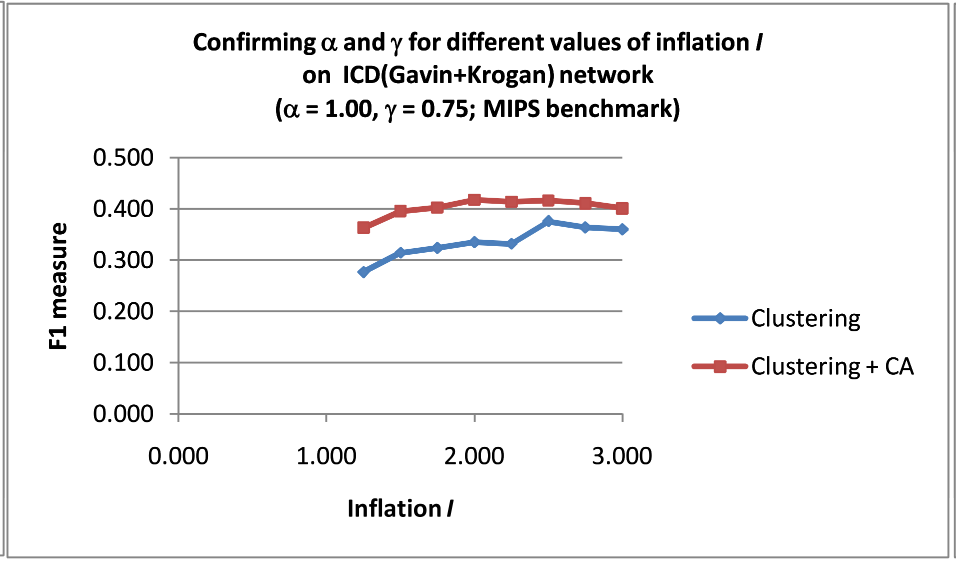

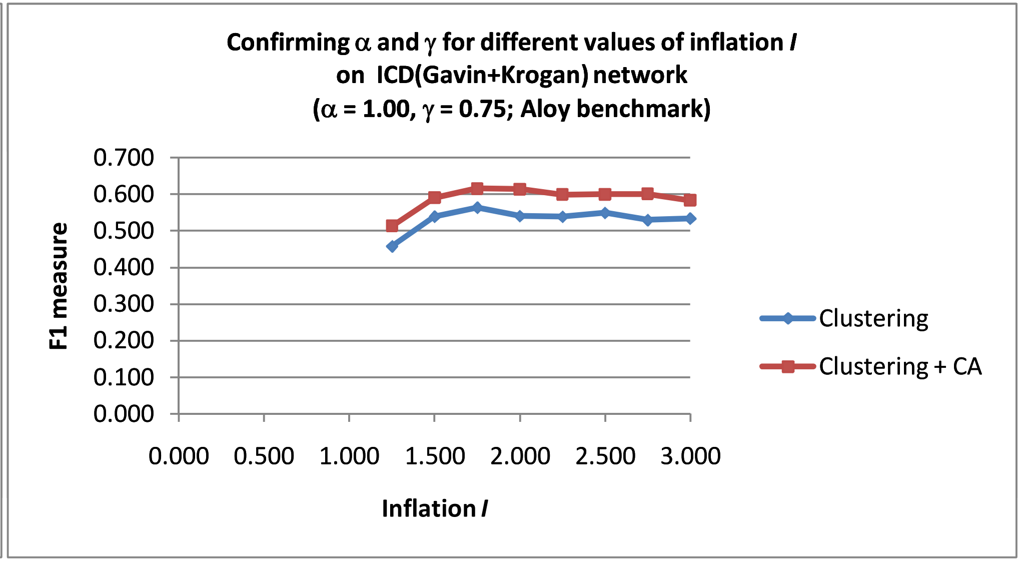

TABLE S7 Relative ranking of the complex detection algorithms

Unscored

Method	WODAK Precision	WODAK Recall	WODAK F1	WODAK Normalized F1	MIPS Precision	MIPS Recall	MIPS F1	MIPS Normalized F1	ALOY Precision	ALOY Recall	ALOY F1	ALOY Normalized F1	Total F1	Norm F1
CMC	0.531	0.330	0.407	1.000	0.363	0.232	0.283	1.000	0.381	0.566	0.455	1.000	3.000	1.000
HACO	0.281	0.467	0.351	0.862	0.162	0.322	0.216	0.761	0.212	0.776	0.333	0.731	2.355	0.785
MCL-CAw	0.248	0.423	0.313	0.768	0.171	0.300	0.218	0.770	0.168	0.684	0.270	0.592	2.130	0.710
CORE	0.215	0.456	0.292	0.718	0.153	0.333	0.210	0.741	0.153	0.776	0.256	0.561	2.020	0.673
MCL	0.226	0.338	0.271	0.665	0.143	0.226	0.175	0.619	0.179	0.556	0.271	0.595	1.879	0.626
MCL-CA	0.224	0.269	0.244	0.601	0.192	0.237	0.212	0.749	0.187	0.539	0.278	0.610	1.960	0.653
COACH	0.139	0.269	0.183	0.450	0.101	0.215	0.137	0.486	0.121	0.487	0.194	0.426	1.361	0.454
			**0.407**				**0.283**				**0.455**		**3.000**	

ICD

Method	WODAK Precision	WODAK Recall	WODAK F1	WODAK Normalized F1	MIPS Precision	MIPS Recall	MIPS F1	MIPS Normalized F1	ALOY Precision	ALOY Recall	ALOY F1	ALOY Normalized F1	Total F1	Norm F1
MCL-CAw	0.620	0.523	0.567	1.000	0.488	0.417	0.450	1.000	0.457	0.787	0.578	0.976	2.976	1.000
HACO	0.654	0.497	0.565	0.995	0.394	0.364	0.378	0.841	0.510	0.707	0.593	1.000	2.837	0.953
MCLO	0.603	0.477	0.533	0.939	0.463	0.371	0.412	0.916	0.463	0.747	0.572	0.965	2.820	0.947
CMC	0.503	0.562	0.531	0.936	0.380	0.430	0.403	0.897	0.345	0.787	0.480	0.810	2.642	0.888
MCL	0.500	0.497	0.498	0.879	0.346	0.397	0.370	0.822	0.426	0.747	0.543	0.916	2.616	0.879
			**0.567**				**0.450**				**0.593**		**2.976**	

FSW

Method	WODAK Precision	WODAK Recall	WODAK F1	WODAK Normalized F1	MIPS Precision	MIPS Recall	MIPS F1	MIPS Normalized F1	ALOY Precision	ALOY Recall	ALOY F1	ALOY Normalized F1	Total F1	Norm F1
MCL-CAw	0.615	0.542	0.576	0.992	0.410	0.437	0.423	1.000	0.513	0.800	0.625	1.000	2.992	1.000
HACO	0.687	0.503	0.581	1.000	0.424	0.371	0.396	0.935	0.535	0.707	0.609	0.974	2.910	0.972
MCL	0.575	0.510	0.541	0.931	0.383	0.404	0.393	0.929	0.475	0.760	0.585	0.935	2.795	0.934
MCLO	0.564	0.471	0.513	0.884	0.388	0.364	0.376	0.888	0.518	0.747	0.612	0.979	2.750	0.919
CMC	0.432	0.549	0.484	0.833	0.278	0.430	0.338	0.798	0.335	0.760	0.465	0.744	2.375	0.794
			**0.581**				**0.423**				**0.625**		**2.992**	

Consolidated

Method	WODAK Precision	WODAK Recall	WODAK F1	WODAK Normalized F1	MIPS Precision	MIPS Recall	MIPS F1	MIPS Normalized F1	ALOY Precision	ALOY Recall	ALOY F1	ALOY Normalized F1	Total F1	Norm F1
MCL-CAw	0.672	0.566	0.614	1.000	0.557	0.433	0.487	1.000	0.467	0.750	0.576	0.979	2.979	1.000
MCLO	0.672	0.552	0.606	0.986	0.546	0.414	0.471	0.967	0.471	0.737	0.575	0.977	2.930	0.984
CMC	0.870	0.462	0.604	0.982	0.727	0.357	0.479	0.983	0.584	0.592	0.588	1.000	2.965	0.995
MCL	0.603	0.545	0.573	0.932	0.414	0.401	0.407	0.836	0.466	0.724	0.567	0.964	2.732	0.917
HACO	0.564	0.411	0.475	0.774	0.396	0.363	0.379	0.777	0.436	0.592	0.502	0.854	2.405	0.807
			**0.614**				**0.487**				**0.588**		**2.979**	

Bootstrap

Method	WODAK Precision	WODAK Recall	WODAK F1	WODAK Normalized F1	MIPS Precision	MIPS Recall	MIPS F1	MIPS Normalized F1	ALOY Precision	ALOY Recall	ALOY F1	ALOY Normalized F1	Total F1	Norm F1
HACO	0.630	0.523	0.572	0.991	0.386	0.375	0.380	1.000	0.465	0.789	0.585	1.000	2.991	1.000
CMC	0.542	0.616	0.577	1.000	0.330	0.414	0.367	0.965	0.374	0.829	0.515	0.881	2.846	0.952
MCL-CAw	0.397	0.512	0.447	0.776	0.236	0.351	0.282	0.742	0.286	0.763	0.416	0.711	2.229	0.745
MCL	0.374	0.494	0.426	0.738	0.271	0.333	0.299	0.785	0.276	0.724	0.400	0.683	2.207	0.738
MCLO	0.372	0.494	0.424	0.736	0.220	0.339	0.267	0.701	0.269	0.723	0.392	0.670	2.108	0.705
			**0.577**				**0.380**				**0.585**		**2.991**	

Method	Relative score	Normalized score
MCL	3.468	0.926
MCLO	3.555	0.949
MCL-CAw	3.745	1.000
CMC	3.628	0.969
HACO	3.733	0.997
	**3.745**	

Method	Relative score	Normalized score
MCL-CAw	3.745	1.000
HACO	3.733	0.997
CMC	3.628	0.969
MCLO	3.555	0.949
MCL	3.468	0.926

TABLE S8 Relative ranking of affinity scored networks for complex detection

		WODAK				MIPS				ALOY					
	Network	Precision	Recall	F1	Norm	Precision	Recall	F1	Norm	Precision	Recall	F1	Norm	Total	Norm
**MCL**	Con	0.603	0.545	0.573	1.000	0.414	0.401	0.407	1.000	0.466	0.724	0.567	0.970	2.970	1.000
	FSW	0.575	0.510	0.541	0.944	0.383	0.404	0.393	0.965	0.475	0.760	0.585	1.000	2.909	0.980
	ICD	0.500	0.497	0.498	0.871	0.346	0.397	0.370	0.908	0.426	0.747	0.543	0.928	2.706	0.911
	BT	0.374	0.494	0.426	0.744	0.271	0.333	0.299	0.733	0.276	0.724	0.400	0.684	2.161	0.728
				**0.573**				**0.407**				**0.585**		**2.970**	
	Network	Precision	Recall	F1	Norm	Precision	Recall	F1	Norm	Precision	Recall	F1	Norm	Total	Norm
**MCLO**	Con	0.672	0.552	0.606	1.000	0.546	0.414	0.471	1.000	0.471	0.737	0.575	0.939	2.939	1.000
	ICD	0.603	0.477	0.533	0.879	0.463	0.371	0.412	0.875	0.463	0.747	0.572	0.934	2.688	0.914
	FSW	0.564	0.471	0.513	0.847	0.388	0.364	0.376	0.798	0.518	0.747	0.612	1.000	2.645	0.900
	BT	0.372	0.494	0.424	0.700	0.220	0.339	0.267	0.567	0.269	0.723	0.392	0.641	1.908	0.649
				**0.606**				**0.471**				**0.612**		**2.939**	
	Network	Precision	Recall	F1	Norm	Precision	Recall	F1	Norm	Precision	Recall	F1	Norm	Total	Norm
**MCL-CAw**	Con	0.672	0.566	0.614	1.000	0.557	0.433	0.487	1.000	0.467	0.750	0.576	0.921	2.921	1.000
	FSW	0.615	0.542	0.576	0.938	0.410	0.437	0.423	0.868	0.513	0.800	0.625	1.000	2.806	0.961
	ICD	0.620	0.523	0.567	0.923	0.488	0.417	0.450	0.923	0.457	0.787	0.578	0.925	2.771	0.949
	BT	0.397	0.512	0.447	0.728	0.236	0.351	0.282	0.579	0.286	0.763	0.416	0.666	1.973	0.675
				**0.614**				**0.487**				**0.625**		**2.921**	
	Network	Precision	Recall	F1	Norm	Precision	Recall	F1	Norm	Precision	Recall	F1	Norm	Total	Norm
**CMC**	Con	0.870	0.462	0.604	1.000	0.727	0.357	0.479	1.000	0.584	0.592	0.588	1.000	3.000	1.000
	BT	0.542	0.616	0.577	0.955	0.330	0.411	0.366	0.764	0.374	0.829	0.515	0.877	2.597	0.866
	ICD	0.503	0.562	0.531	0.880	0.380	0.430	0.403	0.843	0.345	0.787	0.480	0.816	2.538	0.846
	FSW	0.432	0.549	0.484	0.801	0.278	0.430	0.338	0.705	0.335	0.760	0.465	0.791	2.297	0.766
				**0.604**				**0.479**				**0.588**		**3.000**	
	Network	Precision	Recall	F1	Norm	Precision	Recall	F1	Norm	Precision	Recall	F1	Norm	Total	Norm
**HACO**	FSW	0.687	0.503	0.581	1.000	0.424	0.371	0.396	1.000	0.535	0.707	0.609	1.000	3.000	1.000
	BT	0.630	0.523	0.572	0.984	0.386	0.375	0.380	0.961	0.465	0.789	0.585	0.961	2.906	0.969
	ICD	0.654	0.497	0.565	0.972	0.394	0.364	0.378	0.956	0.510	0.707	0.593	0.973	2.902	0.967
	Con	0.564	0.441	0.495	0.852	0.396	0.363	0.379	0.957	0.436	0.592	0.502	0.824	2.634	0.878
				**0.581**				**0.396**				**0.609**		**3.000**	

**Network**	**Total**	**Norm**
Con	4.878	1.000
FSW	4.606	0.944
ICD	4.588	0.941
BT	3.886	0.797
	**4.878**	

**Network**	**Total**	**Norm**
Con	4.878	1.000
FSW	4.606	0.944
ICD	4.588	0.941
BT	3.886	0.797
	**4.878**	
