# Supplementary material for: MCL-CAw: a refinement of MCL for detecting yeast complexes from weighted PPI networks by incorporating core-attachment structure
Source: BMC Bioinformatics. 2010 Oct 12;11:504. doi: 10.1186/1471-2105-11-504 (PMC2965181; doi:10.1186/1471-2105-11-504)
Supplement: Additional files 2 — The MCL-CAw software package: The source code and installation details for the MCL-CAw software. [file 1471-2105-11-504-S2.ZIP › Additional_files_2/mcl-09-149.tar.gz!/mcl-09-149/doc/distindex.html]

MCL manuals


---

# MCL user manuals

### Distribution index 09-149, 1.008

---

### Generic stuff

|  |  |  |
| --- | --- | --- |
| **mcl** - the mcl manual page | html | ps |
| **mclfaq** - the mcl FAQ | html | ps |
| **mcxio** - the mcl matrix format specification | html | ps |
| **mclpipeline** - generic pipeline (parsing/matrix creation/clustering/display) | html | ps |
| **mcx** - interface to basic matrix operations | html | ps |
| **mcxassemble** - prepare input matrix from cooccurrence scores | html | ps |
| **mcxdump** - dump matrices, optionally map indices to labels | html | ps |
| **mcxarray** - transform array data to MCL matrices | html | ps |
| **mcxconvert** - convert between ascii/binary storage types | html | ps |

### Dealing with clustering results

|  |  |  |
| --- | --- | --- |
| **clmformat** - display clusterings in browsable format | html | ps |
| **clmdist** - compute distances between clusterings | html | ps |
| **clminfo** - performance measures for clusterings | html | ps |
| **clmmeet** - compute intersection of clusterings | html | ps |
| **clmmate** - compute best matches between two clusterings | html | ps |
| **clmimac** - Interpret Matrices (c.q. MCL iterands) As Clusterings | html | ps |
| **clmresidue** - extend subgraph clusterings onto encompassing graph | html | ps |
| **clmorder** - reorder indices to represent blocks from different clusterings | html | ps |
| **clmclose** - fetch connected components from graphs or subgraphs | html | ps |

### Dealing with matrices

|  |  |  |
| --- | --- | --- |
| **mcxio** - the mcl matrix format specification | html | ps |
| **mcx** - interface to basic matrix operations | html | ps |
| **mcxsubs** - extract submatrices by specifiying sets of nodes and clusters | html | ps |
| **mcxmap** - remap matrix indices / relabel graph nodes | html | ps |
| **mcxassemble** - prepare input matrix from cooccurrence scores | html | ps |

### Application specific parsers and wrappers

| **BLAST package** - Install with **--enable-blast** | | |
| --- | --- | --- |
| **mclblastline** - pipeline for clustering from blast files | html | ps |
| **mcxdeblast** - prepare input from blast files | html | ps |
