# Supplementary material for: MCL-CAw: a refinement of MCL for detecting yeast complexes from weighted PPI networks by incorporating core-attachment structure
Source: BMC Bioinformatics. 2010 Oct 12;11:504. doi: 10.1186/1471-2105-11-504 (PMC2965181; doi:10.1186/1471-2105-11-504)
Supplement: Additional files 2 — The MCL-CAw software package: The source code and installation details for the MCL-CAw software. [file 1471-2105-11-504-S2.ZIP › Additional_files_2/mcl-09-149.tar.gz!/mcl-09-149/doc/mcxdeblast.html]

The mcxdeblast manual


29 May 2009   
**mcxdeblast**
1.008, 09-149

1.

NAME

2.

SYNOPSIS

3.

DESCRIPTION

4.

STREAM MODE

5.

ASSEMBLE MODE

6.

OPTIONS

7.

AUTHOR

8.

SEE ALSO

## NAME

mcxdeblast — parse BLAST files and stream
output directly into mcl or write mcxassemble input.

## SYNOPSIS

**mcxdeblast** file-name

**mcxdeblast**
**[--score=**<b|e|r> (*bit scores|e-values|normalize bit score*)**]**
**[--m9** (*expect BLAST column format*)**]**
**[--line-mode** abc (*write ID1 ID2 score format*)**]**
**[--sort=**<a|o> (*alphabetic|occurrence sorting*)**]**
**[--xi-dat=**<suf> (*strip <suf> from file-name*)**]**
**[--xo-dat=**<suf> (*add <suf> to base name*)**]**
**[--bcut=**<val> (*bit score cutoff*)**]**
**[--ecut=**<val> (*E-value cutoff*)**]**
**[--tab=**<fname> (*tab file*)**]**
<file-name>

## DESCRIPTION

**mcxdeblast** parses BLAST files.

In *stream mode* mcxdeblast output can be directly streamed to mcl by
using the mcxdeblast **--line-mode**=*abc* option and equipping mcl with
either **--abc** or **--expect-abc**. This can be considered the
easy, lightweight and fast track. This functionality was recently acquired
by mcl.

In the classic and slightly more heavy-handed *assemble mode*, mcxdeblast
creates output files suitable for use by mcxassemble. The latter can in
turn create input suitable for mcl. This is the mode in which mcxdeblast is
employed by **mclblastline**.

## STREAM MODE

Enter stream mode by specifiying **--line-mode**=*abc*.
Use **--m9** if the input is in columnar output. Pipe the result
to a file or directly to mcl. Example invocations can be found
in the mcl manual.

## ASSEMBLE MODE

If the input file is named iput, mcxdeblast will by
default create files iput.hdr, iput.raw, iput.tab,
iput.map, and iput.err. The hdr file contains
information about the number of nodes found. It will be read
by mcxassemble. The raw file contains the co-occurrence
scores between different nodes (peptides), also to be
read by mcxassemble. The co-occurrence scores are in
terms of mcl indentiers, which are subjected to rearranging
by mcxassemble in order to correctly align them with the tab file.
The tab file contains bindings between mcl identifiers
(after rearranging as specified in the map file) and the BLAST labels
(peptide identifiers). Refer to the manual of mcxassemble
for the syntax of a tab file.
The err file contains a list of any errors encountered.

If the **--tab**=**fname** option is used mcxdeblast changes
its behaviour. It will read the file **fname**, and use
the bindings found therein. It will no longer output any
of the hdr, map, or tab files. The hdr
file should be provided by the same application that generated
the specified tab file, and the map file should no longer be
necessary (it might be convenient though and perhaps the
future will bring a new **--map** option).

The **--tab**=**fname** option can be convenient e.g. if
you split a BLAST job over multiple machines and want
to apply mcxdeblast to each of the subresults. You do
this by generating a single central tab file in advance.
Then provide each instance of mcxdeblast with this tab file.
Each instance will generate a raw file. The respective
raw files can simply be concatenated and fed to mcxassemble
using its **-raw** option. Additionally, a hdr file
has to be specified for mcxassemble using its **-hdr**
option. The syntax of hdr files is described in
the mcxassemble manual.
Currently there is usually only one variable
in the hdr file, which is the number of bindings
listed in the tab file. This number, in turn, should equal
the total number of different peptides encountered in the BLAST input.

## OPTIONS

**--tab**=<fname> (*tab file*)

Specify a tab file to use, rather than automatically generating one.

**--score**=<b|e|r> (*bit scores|e-values|norm bit score*)

Specify whether to use bit scores, E-values, or bit scores
normalized by length of the HSP.

**--sort**=<a|o> (*alphabetic|occurrence sorting*)

Specify how to sort labels.

**--m9** (*expect BLAST column format*)

Use this option to parse BLAST output formatted with the -m 9 option.

**--line-mode**=abc (*write ID1 ID2 SCORE format*)

Use this to stream output directly into mcl. Here abc
is a literal; **--line-mode** may acquire other line output
modes.

**--xi-dat**=<suf> (*strip <suf> from <file-name>*)

If set, the suffix <suf> is stripped from file-name in order
to obtain the base name for output. If <file-name> does not
end with suffix, it is assumed to be the base name, and <suf> is
added in order to obtain the (real) file-name.

**--xo-dat**=<suf> (*add <suf> to base name*)

Attaches the suffix to the base name. Use this to distinguish
between different parses. One might for example
use **--score**=*b* **--bcut**=*5* **--xo-dat**=*b*.

**--bcut**=<val> (*bit score cutoff*)

Scores below the cutoff value are ignored.

**--ecut**=<val> (*E-value cutoff*)

Scores below the cutoff value are ignored.

## AUTHOR

Stijn van Dongen.
Jason Stajich implemented the **--m9** option.
Abel Ureta-Vidal and Dinakarpandian Deendayal contributed helpful comments
and fixes.

## SEE ALSO

mclblastline, mcxassemble, mcl,
clmformat.
