# Supplementary material for: MCL-CAw: a refinement of MCL for detecting yeast complexes from weighted PPI networks by incorporating core-attachment structure
Source: BMC Bioinformatics. 2010 Oct 12;11:504. doi: 10.1186/1471-2105-11-504 (PMC2965181; doi:10.1186/1471-2105-11-504)
Supplement: Additional files 2 — The MCL-CAw software package: The source code and installation details for the MCL-CAw software. [file 1471-2105-11-504-S2.ZIP › Additional_files_2/mcl-09-149.tar.gz!/mcl-09-149/doc/mcl.html]

The mcl manual


29 May 2009   
**mcl**
1.008, 09-149

1.

NAME

2.

GETTING STARTED

3.

SYNOPSIS

4.

DESCRIPTION

5.

OPTIONS

6.

PRUNING OPTIONS

7.

PIPELINES

8.

EXAMPLES

9.

APPLICABILITY

10.

FILES

11.

ENVIRONMENT

12.

DIAGNOSTICS

13.

BUGS

14.

AUTHOR

15.

HISTORY/CREDITS

16.

SEE ALSO

17.

REFERENCES

18.

NOTES

## NAME

mcl — The Markov Cluster Algorithm, aka the MCL algorithm.

**mcl** is a cluster algorithm for graphs. A single option controls the
granularity of the output clustering. This is the **-I** *inflation*
option, described further below.

## GETTING STARTED

There are two main modes of invocation. The most accessible is
*label mode*
that assumes label input. The input is then a file or stream in which each
line encodes an edge in terms of two labels and a numerical value, separated
by white space. The most basic example of usage is this:

```
   mcl <-|fname> --abc -o fname-out
```

The output is then a file where each line is a cluster of tab-separated
labels. MCL works natively with a numerical encoding of its input. This
*matrix input* is the second mode of operation. Label mode can save its
input for later use in matrix input. Here is how to do it.

```
   mcl <-|fname> --abc -o fname-out\
      -write-tab map-name -write-graph graph-name
```

Native mode (matrix input) is entered simply by *not* specifying
**--abc**. The input file should then have been created by an earlier
invocation of **-write-graph** *fname* or by other means,
e.g. mcxload. It is
possible to obtain label output in native mode by specifying a label
dictionary, possibly one that was saved earlier using the
**-write-tab** *fname* option. An example is this:

```
   mcl <-|fname> --yield-abc -o fname-out\
      -strict-tab fname-map
```

Here **-strict-tab** tells mcl that the input should not contain
labels other than found in the file **fname-map**. It is possible to
relax this by using **-restrict-tab** *fname-map* or
**-extend-tab** *fname-map*.

Label mode is very convenient for easy and fast exploration. A decided
advantage of full native mode (where both graph input and cluster output are
in matrix format) is that the data can easily be analyzed and subjected to
further processing. Second, input that is stored in native binary format
loads much faster than label data when the input data size grows large. If
neither of these is of concern then label mode may be entirely sufficient.
For more information on label mode and native mode refer to examples in this
manual and mcxio.

**Granularity**  
If you want to explore cluster structure in graphs with MCL, do use
the **-I** *inflation* option with varying parameters
to obtain clusterings at different levels of granularity.

**Clustering from blast files**  
Refer to the group of options discussed with
**--abc**. By way of a small example, consider these.

```
mcxdeblast --line-mode=abc --out=- hsfsp.blast | mcl - --abc -o -
mcxdeblast --m9 --line-mode=abc --out=- hsfsp.blast | mcl - --abc -o -
```

The single hyphens in this example (other than word-separators)
indicate that output is written to STDOUT or read from STDIN.
The blast files are respectively in default and column (-m8 or -m9) format.
They are parsed, the output is sent to mcl, and mcl sends a list of
tab-separated labels to STDOUT. All the abc strings serve to indicate
that the mode of communication between these programs is *label format*.
It is possible to cache the input graph in native mcl matrix format:

```
mcxdeblast --line-mode=-abc --out=- hsfsp.blast | \
   mcxload -abc - --stream-mirror -o hsfsp.mcx -write-tab hsfsp.tab --write-binary
mcl hsfsp.mcx -use-tab hsfsp.tab -o  hsfsp.my-nice-clustering
```

The matrix is written in binary format to speed up
subsequent reads. The speed-up factor is approximately ten-fold.
For large graphs it is recommended to use binary format.
Keep in mind though that it is not portable across machines
and is not garantueed to be portable across different versions
of mcl or differently compiled versions of mcl.
**mcl** also accepts the **--write-binary** option, ensuring that
graphs written with **-write-graph** are output in binary
format.
The mcxload **--mirror** option ensures that
the resulting graph is undirected. When mcl itself is in charge of processing
the label input (rather than mcxload as in the example under discussion)
it is not necessary to instruct mcl to do so.

## SYNOPSIS

The example invocation below assumes matrix input, as described in the
mcxio section. Switching to label mode requires the input file to be in
label format and the addition of the **--abc** option.

**mcl** <-|fname>
**[-I** f (*inflation*)**]**
**[-o** str (*fname*)**]**
**[-scheme** k (*resource scheme*)**]**

These options are sufficient in 95 percent of the cases or more. The first
argument must be the name of a file containing a graph/matrix in the mcl
input format, or a hyphen to read from STDIN. With respect to clustering,
the **-I** option and **-scheme**
option are foremost relevant.

A mechanism for pipelines is supported (as of the first 1.003 release).
Refer to the PIPELINES section for more information.
A prepackaged pipeline for BLAST data is present in the form of
mclblastline. As of release 1.006 a lightweight BLAST clustering
mechanism is also supported. GETTING STARTED is a small introduction,
with some examples of using BLAST results.

The full listing of **mcl** options is shown below, separated
into parts corresponding with functional aspects such
as clustering, threading, verbosity, pruning and resource management,
automatic output naming, and dumping.
The **-scheme** parameter provides a single access point to the
pruning options, and should be sufficient in most cases.
**mcl** allows comprehensive tuning and access to its internals
for those who are interested, so it has many options.

**Baseline clustering options**  
**[-I** <num> (*inflation*)**]**
**[-o** str (*fname*)**]**
**[-od** str (*directory*)**]**
**[-scheme** k (*resource scheme*)**]**
**[--shadow-vl** (*shadow distant nodes*)**]**
**[-c** <num> (*reweight loops*)**]**

**Stream options**  
**[--abc** fname (*expect/write labels*)**]**
**[--expect-abc** (*expect labels*)**]**
**[--yield-abc** fname (*write labels*)**]**
**[-use-tab** fname (*use mapping to write*)**]**
**[-strict-tab** fname (*use mapping to enforce*)**]**
**[-restrict-tab** fname (*use mapping to restrict*)**]**
**[-extend-tab** fname (*use mapping and extend*)**]**
**[-write-tab** fname (*write mapping*)**]**

**Transform options**  
**[-tf** <tf-spec> (*transform input matrix values*)**]**
**[-abc-tf** <tf-spec> (*transform input stream values*)**]**
**[--abc-log** (*take logarithm of stream values*)**]**
**[--abc-neg-log** (*take negative logarithm of stream values*)**]**

**Cache options**  
**[-write-graph** fname (*write graph*)**]**
**[-write-graphx** fname (*write transformed graph*)**]**
**[-write-expanded** fname (*write expanded graph*)**]**

**Additional clustering options**  
**[-l** n (*initial iteration number*)**]**
**[-L** n (*main iteration number*)**]**
**[-i** f (*initial inflation*)**]**
**[-lint-k** <num> (*assimilate small clusters entirely*)**]**
**[-lint-l** <num> (*try to pry nodes from small clusters*)**]**
**[--adapt-local** (*localize inflation (experimental)*)**]**
**[--shadow** mode (*modulation type*)**]**
**[-shadow-s** f (*boost factor*)**]**

**Input manipulation options**  
**[-pi** f (*pre-inflation*)**]**
**[-ph** f (*pre-inflation, max-bound*)**]**
**[-if** f (*start-inflation*)**]**
**[-ceil-nb** n (*reduce neighbourhods*)**]**
**[--discard-loops=**y/n (*discard y/n loops in input*)**]**

**Clustering result options**  
**[-sort** str (*sort mode*)**]**
**[-overlap** str (*overlap mode*)**]**
**[--output-limit=**y/n (*write limit matrix*)**]**
**[--force-connected=**y/n (*analyze components*)**]**
**[--check-connected=**y/n (*analyze components*)**]**
**[--analyze=**y/n (*performance criteria*)**]**
**[--show-log=**y/n (*show log*)**]**
**[--append-log=**y/n (*append log*)**]**

**Verbosity options**  
**[-q** spec (*log levels*)**]**
**[-v** str (*verbosity type on*)**]**
**[-V** str (*verbosity type off*)**]**
**[--show** (*print (small) matrices to screen*)**]**

**Thread options**  
**[-te** k (*#expansion threads*)**]**

**Output file name and annotation options**  
**[-o** str (*fname*)**]**
**[-ap** str (*use str as file name prefix*)**]**
**[-aa** str (*append str to suffix*)**]**
**[-az** (*show output file name and exit*)**]**
**[-ax** (*show output suffix and exit*)**]**
**[-annot** str (*dummy annotation option*)**]**

**Dump options**  
**[-dump-interval** i:j (*dump interval*)**]**
**[-dump-modulo** k (*dump modulo*)**]**
**[-dump-stem** stem (*dump file stem*)**]**
**[-dump** str (*type*)**]**
**[-dump-subi** spec (*index list for submatrix dump*)**]**
**[-dump-subd** spec (*domain list for submatrix dump*)**]**
**[-dump-dom** fname (*domain matrix file*)**]**
**[-digits** n (*printing precision*)**]**
**[--write-binary** (*write matrices in binary format*)**]**

**Info options**  
**[--jury-charter** (*explains jury*)**]**
**[--version** (*show version*)**]**
**[-how-much-ram** k (*RAM upper bound*)**]**
**[-h** (*most important options*)**]**
**[--apropos** (*one-line description for all options*)**]**
**[-z** (*show current settings*)**]**
**[-az** (*show output file name and exit*)**]**
**[-ax** (*show output suffix and exit*)**]**
**[--show-schemes** (*show resource schemes*)**]**

**Pruning options**  
The following options all pertain to the various pruning strategies that can
be employed by **mcl**. They are described in the PRUNING OPTIONS
section, accompanied by a description of the mcl pruning strategy.
If your graphs are huge
and you have an appetite for tuning, have a look at the following:

**[-p** f (*cutoff*)**]**
**[-P** n (*1/cutoff*)**]**
**[-S** n (*selection number*)**]**
**[-R** n (*recovery number*)**]**
**[-pct** f (*recover percentage*)**]**
**[-my-scheme** n (*tag custom scheme*)**]**
**[-warn-pct** n (*prune warn percentage*)**]**
**[-warn-factor** n (*prune warn factor*)**]**
**[--adapt** (*pruning*)**]**

The first argument of **mcl** must be a file name, but some options are allowed
to appear as the first argument instead. These are the options that cause
mcl to print out information of some kind, after which it will gracefully
exit. The full list of these options is

**-z**,
**-h**,
**--apropos**,
**--version**,
**--show-settings**,
**--show-schemes**,
**--jury-charter**,
**-how-much-ram** *k*.

## DESCRIPTION

**mcl** implements the **MCL algorithm**, short for the **Markov cluster
algorithm**, a cluster algorithm for graphs developed by Stijn van Dongen at
the Centre for Mathematics and Computer Science in Amsterdam, the
Netherlands. The algorithm simulates flow using two simple algebraic
operations on matrices.
The inception of this flow process and the theory behind it are
described elsewhere (see REFERENCES). Frequently asked questions
are answered in the mclfaq section.
The program described here is a fast threaded implementation written by the
algorithm's creator with contributions by several others. Anton Enright
co-implemented threading; see the HISTORY/CREDITS section for a complete
account.
See the APPLICABILITY section for a description of the type of
graph mcl likes best, and for a qualitative assessment of its speed.
**mcl** is accompanied by several other utilities for analyzing clusterings and
performing matrix and graph operations; see the SEE ALSO section.

The first argument is the input file name,
or a single hyphen to read from stdin. The rationale for
making the name of the input file a fixed parameter is that you typically do
several runs with different parameters. In command line mode it is
pleasant if you do not have to skip over an immutable parameter all the
time.

The **-I** *f* option is the main control,
affecting cluster granularity. Using **mcl** is as simple as
typing (assuming a file *proteins* contains a matrix/graph
in native matrix format)

```
mcl proteins -I 2.0
```

The above will result in a clustering written to the file
named *out.proteins.I20s2*. It is - of course -
possible to explicitly specify the name of the output file
using the **-o** *fname* option. Refer to the
**-ap** option for a description of mcl's procedure
in automatically constructing file names from it parameters.

In native mode clusterings are stored as matrices
- this is discussed in the mcxio section.
You presumably want to convert the output to something that
is easier to interpret. The native mcl matrix format is perhaps
unpleasant to parse in the quick and dirty way. You can use

```
mcl proteins -I 2.0 -use-tab proteins.tab --yield-abc
```

to get a line/tab based output format,
each line containing a cluster in the form of tab-separated labels.
Here **proteins.tab** should be a tab file previously
created by mcxdeblast or mcl.
Refer to mcxio for more information on tab files,
and the entries grouped under **--abc** for an extensive
discussion of the various ways in which mcl can combine
label format and matrix format in input and output.

In finding good **mcl** parameter settings for a particular domain,
or in finding cluster structure at different levels of granularity,
one typically runs mcl multiple times for varying values of f (refer
to the **-I** *inflation* option for further information).

**NOTE**  
mcl interprets the matrix
entries or graph edge weights as **similarities**, and it likes
**undirected input graphs** best. It can handle directed graphs, but any
node pair (i,j) for which w(i,j) is much smaller than w(j,i) or vice versa
will presumably have a slightly negative effect on the clusterings output by
mcl. Many such node pairs will have a distinctly negative effect, so try to
make your input graphs undirected. How your edge weights are computed may
affect mcl's performance. In protein clustering, one way to go is to
choose the negated logarithm of the BLAST probabilities (see
REFERENCES).

**mcl**'s default parameters should make it quite fast under almost all
circumstances. Taking default parameters, mcl has been used to generate
good protein clusters on 133k proteins, taking 10 minutes running time on a
Compaq ES40 system with four alpha EV6.7 processors. It has been applied
(with good results) to graphs with 800k nodes, and if you have the memory
(and preferably CPUs as well) nothing should stop you from going further.

For large graphs, there are several groups of parameters available for
tuning the mcl computing process, should it be necessary. The easiest thing
to do is just vary the **-scheme** option. This
triggers different settings for the group of pruning parameters
**-p/-P**, **-R**, **-S**, and
**-pct**. The default setting corresponds with
**-scheme** **6**.
When doing multiple mcl runs for the same graphs with different
**-I** settings (for obtaining clusterings at different levels
of granularity), it can be useful to factor out the first bit
of computation that is common to all runs, by using
the **-write-expanded** option one time
and then using **-if** *inflation* for each run in the set.
Whether mcl considers a graph large depends mainly on the graph
connectivity; a highly connected graph on 50,000 nodes is large to
mcl (so that you might want to tune resources) whereas a sparsely
connected graph on 500,000 nodes may be business as usual.

**mcl** is a memory munger. Its precise appetite depends on the resource
settings. You can get a rough (and usually much too pessimistic) upper
bound for the amount of RAM that is needed by using the
**-how-much-ram** option. The corresponding
entry in this manual page contains the simple formula via which the upper
bound is computed.

Other options of interest are the option to specify threads
**-te**, and the verbosity-related options
**-v** and **-V**.
The actual settings are shown with **-z**, and for graphs with
at most 12 nodes or so you can view the MCL matrix iterands on screen
by supplying **--show** (this may give some
more feeling).

MCL iterands allow a generic interpretation as clusterings as well. The
clusterings associated with early iterands may contain a fair amount of
overlap. Refer to the **-dump** option, the mclfaq
manual, and the **clm imac** utility (Interpret Matrices As Clusterings).
Use **clm imac** only if you have a special reason; the normal usage of **mcl** is
to do multiple runs for varying **-I** parameters and use the
clusterings output by mcl itself.

Under very rare circumstances, **mcl** might get stuck in a seemingly infinite
loop. If the number of iterations exceeds a hundred and the *chaos*
indicator remains nearly constant (presumably around value 0.37), you can
force mcl to stop by sending it the ALRM signal (usually done
by **kill -s ALRM** *pid*). It will finish the current
iteration, and interpret the last iterand as a clustering. Alternatively, you
can wait and mcl might converge by itself or it will certainly stop after
10,000 iterations (the default value for the **-L** option). The
most probable explanation for such an infinite loop is that the input graph
contains the flip-flop graph of node size three as a subgraph.

The creator of this page feels that manual pages are a valuable resource,
that online html documentation is also a good thing to have, and
that info pages are way *way* ahead of their time. The
NOTES section explains how this page was created.

In the OPTIONS section options are listed in order of
importance, with related options grouped together.

## OPTIONS

**-I** f (*inflation*)

Sets the main inflation value to f. This value is the main handle
for affecting cluster granularity. It is usually chosen somewhere
in the range [1.2-5.0]. **-I** **5.0** will tend to result
in fine-grained clusterings, and **-I** **1.2** will tend to
result in very coarse grained clusterings. Your mileage will vary
depending on the characteristics of your data. That is why it is
a good idea to test the quality and coherency of your clusterings
using **clm dist** and **clm info**. This will most likely reveal that
certain values of **-I** are simply not right for your data. The
**clm dist** section contains a discussion of how to use the cluster
validation tools shipped with **mcl** (see the SEE ALSO section).

With low values for **-I**, like **-I** **1.2**, you should be
prepared to use more resources in order to maintain quality of
clusterings, i.e. increase the argument to the
**-scheme** option.

**-o** str (*fname*)

Output the clustering to file named fname. It is possible to send the
clustering to stdout by supplying **-o** **-**. If either one of
**--abc**, **--yield-abc**, or
**-use-tab** *tab-file* is used the output will be in label format —
provided a tab file is specified or the input is in label format. Otherwise
the clustering is output in the mcl matrix format; see the mcxio
section for more information on this. Refer also to the group of options
discussed at **--abc**.

Look at the **-ap** *prefix* option and its siblings for the
automatic naming constructions employed by **mcl** if the **-o** option is
not used.

**-od** <dir> (*directory*)

Specify the directory where to output clusterings. This is useful
when you let mcl automatically construct the output file name,
but want the clustering in a directory different from the
one where the input graph resides.

**-shadow** mode (*apply shadowing*)

**-shadow-s** <num> (*boost factor (experimental)*)

**--shadow-vl** (*shadow distant nodes*)

**EXPERIMENTAL**  
*These options are intended and useful for hierarchical
clustering with* mclcm.
**--shadow-vl** is the same as **-shadow** **vl**
and is at present the best mode to choose.

Use these to weaken the connectivity of certain
classes of nodes. This means that the effect of the edges
in which they participate on the final clustering result is diminished.
This is done such that the extent to which a node exhibits certain
characteristics is correlated with the extent to which its connectivity is
weakened. The different types of characteristics that can be regulated
are

**vl**

value low - nodes for which the average value of its edges
is low compared with the same number across its neighbours.

**vh**

value high - nodes for which the average value of its edges
is high compared with the same number across its neighbours.

**el**

edge low - nodes for which the number of neighbours
is low compared with the same number across its neighbours.

**eh**

edge high - nodes for which the number of neighbours
is high compared with the same number across its neighbours.

The characteristics above, e.g. **vl**, correspond to the modes
that can be passed to the **-shadow** argument. The first mode **vl**
is the one most likely to be useful. Combining different modes is possible
but may lead to unbalanced clusterings. Using the mode **el** in **mclcm**
may similarly lead to unbalanced hierachies.

The boost factor can be set e.g. to 2.0 to increase the effect of shadowing.
The default setting is 1.0.

**-c** <num> (*reweight loops*)

As the final step of loop computation (i.e. after initialization
and shadowing), all loop weights are multiplied by **<num>**,
if supplied.

**--discard-loops**=y/n (*discard loops in input*)

By default **mcl** will remove any loops that are present in the input. Use
**--discard-loops**=**n** to turn this off. Bear in mind that loops will
still be modified in all cases where the loop weight is not maximal among
the list of edge weights for a given node.

**--abc** fname (*expect/write labels*)

**--expect-abc** (*expect labels*)

**--yield-abc** fname (*write labels*)

**-use-tab** fname (*use mapping to write*)

**-strict-tab** fname (*use mapping to enforce*)

**-restrict-tab** fname (*use mapping to restrict*)

**-extend-tab** fname (*use mapping and extend*)

**-write-tab** fname (*write mapping*)

These items all relate to label input and/or label output.
**--abc** tells mcl to expect label input and output clusters
in terms of those labels.
It is equivalent to the combination of **--expect-abc**
and **--yield-abc**.

**-restrict-tab** and **-strict-tab**
can be used both with label input and native format.
When label input is used, they restrict, respectively require
labels to be present in the tab file.
When native input is used they restrict, respectively require
indices to be present in the tab domain.
**-strict-tab** fails in the face of exceptions, **-restrict-tab**
will simply ignore them.
**-strict-tab** and **-restrict-tab** do *not*
automatically yield label output. You need **--abc** or
**--yield-abc**.

**-extend-tab** is only useful when label input is used.
It will create a new label/index mapping when a label is not found
in the tab file. Presumably you want to use the **-write-tab**
as well then.

**-use-tab** is only useful when matrix input is used.
It will use the tab file to convert the output to labels; it does
not hitch on indices missing from the tab file. Take a pick
from **-restrict-tab** or **-strict-tab** *and*
**--abc** or **--yield-abc** if that is what you want.

**-write-tab** can be used to preserve the tab file
that was constructed from label input, either from scratch
or by extension from a previous tab file in case **-extend-tab**
was used.

**NOTE**  
in all its dealings with tab files, mcl will only accept those
for which the associated domain is *canonical*, that is,
domains for which the indices range from zero to some number N
without omissions. It is possible to hook up any tab file to mcl,
but it requires mcxdump to act as an intermediary -
mcxdump has no such limitations. This need in
general not be of concern to you. If a tab file is created by
mcl or mcxdeblast it will always be canonical.

**-tf** <tf-spec> (*transform input matrix values*)

**-abc-tf** <tf-spec> (*transform input stream values*)

**--abc-log** (*take logarithm of stream values*)

**--abc-neg-log** (*take negative logarithm of stream values*)

**-tf** transforms the values of the input matrix according
to **<tf-spec>**. **-abc-tf** transforms the stream values
(when **--abc** or **--expect-abc** is used)
according to **<tf-spec>**.
**--abc-neg-log** and **--abc-log**
respectively imply that the stream input values are
first replaced by their (negative) logarithm.
The reason for their existence is documented in mcxio.
For a description of the transform language excpected/accepted
in **<tf-spec>** refer to the same.

**-write-graph** fname (*write graph*)

**-write-graphx** fname (*write transformed graph*)

**-write-expanded** fname (*write expanded graph*)

If you work with label input, **-write-graph** can be
used to save the matrix mcl constructs from the input stream.
**-write-graphx** is similar except that it applies to the graph after
transformations have been applied to it (such as specified by the
**-tf** option). In a following mcl invocation, you can use this
graph rather than the label input. This should greatly speed up matters.
Presumably the first time around you have use the
**-write-tab** *my.tab* option. You can put that my.tab file to
good use by passing it in as **-use-tab** *my.tab* the second time
around. The session below puts everything together.

```
mcl xyz.data --abc -I 2.0 -o xyz.cls-I20 --write-binary\
            -write-graph xyz.mci -write-tab xyz.tab
mcl xyz.mci -I 2.4 -use-tab xyz.tab -o xyz.cls-I24
```

The **--write-binary** option is useful for large graphs as it
will drastically speed up subsequent load times. Otherwise it
is not necessary, and bear in mind that binary format is not
portable across machines and it is not garantueed to be portable
across different version of mcl.
For very large graphs it could be a bit wasteful to load a large tab
structure into memory. The second line can then be replaced by

```
mcl xyz.mci -I 2.4 -o -|\
mcxdump -imx - --no-values --dump-rlines\
            -tabr xyz.tab -o xyz.cls-I24
```

Admittedly this is beginning to look like black magic, but truthfully
it is not. mcxdump
simply needs to be told how it should format its output. It needs to know
it should dump the matrix columns (clusters in this case) on a per-line
basis, that it should not dump the index that identifies the cluster
(an arbitrary rank in our case), and that there is no need to output values.
**mcxdump** furthermore does not know how the tab file relates to
its input matrix, whereas mcl previously had the advantage of knowing.
Hence **-tabr** is telling mcxdump that the *row domain*
in the clustering matrix identify the nodes.

The first step in almost any mcl run is that the matrix constructed from the
input is *squared* or *expanded*. This is a rather costly operation
if the input size is large. When you are doing multiple runs it can thus be
useful to cache the expanded matrix at the cost of a little more hassle.
Use **-write-expanded** *fname* to write this matrix to *fname*.
In subsequent runs supply *fname* as the input argument to mcl and
use **-if** *num* to indicate that the first thing to apply should be
inflation with parameter *num*. Combining our previous two
examples then yields

```
mcl xyz.data --abc -I 2.0 -o xyz.cls-I20 --write-binary\
            -write-expanded xyz.mxp -write-tab xyz.tab
mcl xyz.mxp -if 2.4 -I 2.4 -o -|\
mcxdump -imx - --no-values --dump-rlines\
            -tabr xyz.tab -o xyz.cls-I24
```

Behold, this is a very time and space efficient setup.

**-scheme** k (*use a preset resource scheme*)

There are currently seven different resource schemes, indexed 1..7.
High schemes result in more expensive computations that may possibly be
more accurate. The default scheme is 4. When **mcl** is done, it will give a
grade (the so called *jury synopsis*) to the appropriateness of the
scheme used. *A low grade does not necessarily imply that the
resulting clustering is bad* - but anyway, a low grade should be reason
to try for a higher scheme. The grades are listed in the
PRUNING OPTIONS section under the
**-nj** option.

The PRUNING OPTIONS section contains an elaborate description
of the way **mcl** manages resources, should you be interested.
In case you are worried about the validation of the resulting
clusterings, the mclfaq section
has several entries discussing this issue. The bottom line is
that you have to compare the clusterings resulting from different
schemes (and otherwise identical parameters) using utilities
such as **clm dist**, **clm info** on the one hand, and your
own sound judgment on the other hand.

If your input graph is extremely dense, with an average node degree
(i.e. the number of neighbours per node) that is somewhere above
500, you may need to filter the input graph by removing edges,
for example by using the
**-ceil-nb** option.

**-lint-k** <numk> (*cluster size assimilation threshold*)

**-lint-l** <numl> (*cluster size pry threshold*)

These options will result in postprocessing on the clustering, reallocating
nodes that seems to have siphoned the wrong way. The input matrix will be
reread for this.

When applied to networks with inhomogenously distributed edge density
characteristics the mcl process will sometimes cause smaller
clusters/sparse areas to suck in border nodes which 1) have only few
edges to that cluster/area and 2) seem to have been sucked out of a much
denser cluster into which they would fit beautifully. This is fully in line
with the flow characteristics of mcl but a largely unwanted phenomenon. The
postprocessing step was added as a remedy for this problem should it
manifest itself. It is based on the efficiency criterion described in
[1] and [5] (there called *coverage*).

With **-lint-l** a node is tentatively reallocated if this will improve
its effiency, and if it is initially in a cluster of size at most *numl*.
After all nodes that are candidate for reallocation have been
identified the proposed new clustering is accepted if it increases the total
edge weight captured. This procedure is iterated until no further
reallocations are possible. The efficiency measure is a conservative
criterion in that it does not unduly favour larger clusters over smaller
clusters. For example, if a node has 4 neighbours in a cluster of size 8 and
it has a further 8 neighbours in a cluster of size 100 the efficiency for
the first cluster (relative to that node) will generally be higher than the
efficiency for the second cluster.

With **-lint-k** clusters up to the given size *numk*
are assimilated by a larger cluster if a suitable candidate is found.
This is based on the same criteria as described above, except
that a cluster is absorbed in its entirety.

**--adapt-local** (*localize inflation (experimental)*)

**Experimental**  
Inflation is adapted depending on the amount of local dispersion,
where dispersion is a measure for how quickly stochastic flow
disperses throughout the graph. Regions with a low/high clustering
coefficient will have comparatively high/low dispersion.
For regions with high dispersion (low clustering coefficient)
inflation is adapted towards a higher value, for regions with
low dispersion (high clustering coefficient) inflation is adapted
towards a lower value.

**--show-schemes** (*show preset resource schemes*)

Shows the explicit settings to which the different preset schemes
correspond.

The characteristics are written in the same format (more or less) as
the output triggered by **-v** **pruning**.

**-V** str (*verbosity type off*)

See the **-v** option below.

**-v** str (*verbosity type on*)

These are the different verbosity modes:

**pruning**  
**explain**  
**cls**  
**all**

**-q** spec (*log levels*)

To make mcl as quiet as can be, add
**-q** **x** **-V** **all** to the command line.

The **-q** option governs a general logging mechanism.
The format accepted is described in the tingea.log manual page.

The other options govern verbosity levels specific to mcl. **-v** **all**
turns them all on, **-V** **all** turns them all off. **-v** *str* and
**-V** *str* turn on/off the single mode *str* (for *str*
equal to one of **pruning**, **cls**, or **explain**). Each verbosity
mode is given its own entry below.

**-v** **explain**

This mode causes the output of explanatory headers illuminating the
output generated with the **pruning** verbosity mode.

**-v** **pruning**

This mode causes output of resource-related quantities. It has
a separate entry in the PRUNING OPTIONS section.

**-v** **cls**

This mode (on by default) prints a terse list of characteristics of the
clusterings associated with intermediate iterands. The characteristics are
**E/V**, **cls**, **olap**, and **dd**. They respectively stand for the
number of outgoing arcs per node (as an average), the number of clusters in
the overlapping clustering associated with the iterand, the number of nodes
in overlap, and the *dag depth* associated with the DAG (directed acyclic
graph) associated with the iterand. For more information on this DAG refer
to the **-dump** option description in this manual and also
mclfaq.

**-aa** str (*append str to suffix*)

See the **-ap** option below.

**-ap** str (*use str as file name prefix*)

If the **-o** *fname* option is not used,
**mcl** will create a file name (for writing output to) that
should uniquely characterize the important parameters used in the
current invocation of mcl. The default format is **out.fname.suf**,
where **out** is simply the literal string out, **fname** is the
first argument containing the name of the file (with the graph) to be
clustered, and where **suf** is the suffix encoding a set of parameters
(described further below).

The **-ap** *str* option specifies a prefix to use
rather than **out.fname** as sketched above.
However, **mcl** will interpret the character '=', if present
in *str*, as a placeholder for the input file name.

If the **-aa** *str* option is used, **mcl** will append
**str** to the suffix **suf** created by itself.
You can use this if you need to encode some extra information in the
file name suffix.

The suffix is constructed as follows. The **-I** *f*
and **-scheme** parameter are always encoded.
The **-pi** *f*, **-l** *k*, and **-i** *f*
options are only encoded if they are used. Any real argument *f*
is encoded using *exactly one* trailing digit behind the decimal
separator (which itself is not written). The setting **-I** **3.14**
is thus encoded as I31. The **-scheme** option is encoded using the
letter 's', all other options mentioned here are encoded as themselves
(stripped of the hyphen). For example

```
mcl small.mci -I 3 -c 2.5 -pi 0.8 -scheme 5
```

results in the file name out.small.mci.I30s5c25pi08.
If you want to know beforehand what file name will be produced,
use the **-az** option.

**-az** (*show output file name and exit*)

**-ax** (*show output suffix and exit*)

If **mcl** automatically constructs a file name, it can be helpful to known
beforehand what that file name will be. Use **-az** and mcl will
write the file name to STDOUT and exit. This can be used if mcl is
integrated into other software for which the automatic creation of
unique file names is convenient.

By default mcl incorporates the input file name into the output file
name and appends a short suffix describing the most important
option settings. Use **-ax** to find out what that suffix is.
This can be useful in wrapper pipeline scripts such as clxcoarse.

**-annot** str (*dummy annotation option*)

**mcl** writes the command line with which it was invoked to the output
clustering file. Use this option to include any additional
information. MCL does nothing with this option except copying
it as just described.

**-te** k (*#expansion threads*)

Threading is useful if you have a multi-processor system. **mcl** will
spawn *k* threads of computation. If these are computed
in parallel (this depends on the number of CPUs available to the
mcl process) it will speed up the process accordingly.

When threading, it is best not to turn on pruning verbosity
mode if you are letting mcl run unattended, unless you want to
scrutinize its output later. This is because it makes **mcl** run
somewhat slower, although the difference is not dramatic.

**-l** n (*initial iteration number*) (small letter ell)

The number of times **mcl** will use a different inflation value
before it switches to the (main) inflation given by the **-I**
(capital eye) option. The different value is called *initial
inflation* and is tunable using the **-i** *f*
option (default value f=2.0). The default value (to **-l**)
is zero. This option supplies new ways of affecting cluster
granularity, e.g. by supplying

```
mcl proteins -i 1.4 -l 2 -I 4.0
```

one lets expansion prevail during the first two iterations,
followed by inflation catching up (in a figurative way of writing).
This may be useful in certain cases, but this type of experiment
is *certainly secondary* to simply varying **-I** (capital eye).

**-L** n (*main iteration number*)

Normally, **mcl** computes the MCL process until it has converged
fully to a doubly idempotent matrix. The number of iterations
required is typically somewhere in the range 10-100.
The first few iterations generally take the longest time.
The **-L** option can be used to specify the number of
iterations mcl may do at most. When this number is reached,
mcl will output the clustering associated with the iterand
last computed.

**-i** f (*initial inflation*)

The inflation value used during the first n iterations,
where n is specified by the **-l** (ell) option.
By default, n=0 and f=2.0.

**-pi** f (*pre-inflation*)

**-ph** f (*pre-inflation, max-bound*)

If used, **mcl** will apply inflation one time to the input graph
before entering the main process. This can be useful for
making the edge weights in a graph either more homogeneous (which
may result in less granular clusterings) or more heterogeneous
(which may result in more granular clusterings).
Homogeneity is achieved for values *f* less than one,
heterogeneity for values larger than one.
Values to try are normally in the range [2.0,10.0].

The **-ph** option is special in that it does not rescale
columns to be stochastic. Instead, it rescales columns so that
the maximum value found in the column stays the same after
inflation was applied. This is for use in conjunction with
**--shadow**=*vl*, as the latter option is affected by
absolute differences in edge weights.

**-if** f (*start-inflation*)

If used, **mcl** will apply inflation one time to the input graph
before entering the main process. The difference with
**-pi** is that with the latter option mcl may apply
certain transformations after reading in the matrix such
as adding or modifying loops. The purpose of
the **-if** (mnemonic for *inflation-first*)
option is to use it on graphs saved
with the **--write-expanded** option and convey
to mcl that it should not apply those transformations.

**-di** i:j (*dump interval*)

**-di** *all*

**-dump-interval** *i:j*

Dump during iterations i..j-1. Use *all* to dump in all
iterations. See the **-dump** *str* option below.

**-dm** k (*dump i+0..i+k..*)

**-dump-modulo** *k*

Sampling rate: select only these iterations in the dump interval.
See the **-dump** *str* option below.

**-ds** stem (*file stem*)

**-dump-stem** *stem*

Set the the stem for file names of dumped
objects (default *mcl*). See the **-dump** *str* option below.

**-dump-subi** spec (*index list for submatrix dump*)

**-dump-subd** spec (*domain list for submatrix dump*)

**-dump-dom** fname (*domain matrix file*)

**-dump-subi** specifies a range of indices which will be
used to select the extended principal submatrix.
Argument *spec* can be a comma-separated
list of single integers and integer ranges. Ranges are denoted by
two integers separated by a hyphen.

If **-dump-dom** is used and specifies a matrix file,
the indices specified in the **-dump-subd** option should index
columns in that matrix. These columns are merged and added to the
list of entries used in selecting the extended principal submatrix.

**-dump** str (*type*)

*str* is checked for substring occurrences of the following entries.
Repeated use of **-dump** is also allowed.

**ite**  
**dag**  
**cls**  
**chr**  
**lines**  
**cat**

**lines** and **cat** change the mode of dumping. The first
changes the dump format to a line based pairwise format rather
than the default mcl matrix format. The second causes all
dumped items to be dumped to the default stream used for the
output clustering, which is appended at the end.

The **ite** option writes **mcl** iterands to file. The **cls**
option writes clusterings associated with mcl iterands to file.
These clusters are obtained from a particular directed acyclic graph
(abbreviated as DAG) associated with each iterand. The **dag** option
writes that DAG to file. The DAG can optionally be further
pruned and then again be interpreted as a
clustering using **clm imac**, and **clm imac** can also
work with the matrices written using the **ite** option.
It should be noted that clusterings associated with intermediate
iterands may contain overlap, which is interesting in
many applications. For more information
refer to mclfaq and the REFERENCES section below.

The **result** option dumps the usual MCL clustering.

The **chr** option says, for each iterand I, to output a matrix C with
characteristics of I. C has the same number of columns as I. For each
column k in C, row entry 0 is the diagonal or 'loop' value of column k in
I *after* expansion and pruning, and *before* inflation and
rescaling. Entry 1 is the loop value *after* inflation and rescaling.
Entry 2 is the center of column k (the sum of its entries squared)
computed *after* expansion and *before* pruning, entry 3 is the
maximum value found in that column at the same time. Entry 4 is the
amount of mass kept for that column *after pruning*.

The **-ds** option sets the stem for file names of dumped
objects (default *mcl*). The **-di** and **-dm**
options allow a selection of iterands to be made.

**-digits** n (*printing precision*)

This has two completely different uses. It sets
the number of decimals used for pretty-printing **mcl** iterands
when using the **--show** option (see below),
and it sets the number of decimals used for writing
the expanded matrix when using the **-write-expanded** option.

**--show** (*print matrices to screen*)

Print matrices to screen. The number of significant digits to be
printed can be tuned with **-digits** *n*. An 80-column screen
allows graphs (matrices) of size up to 12(x12) to be printed with
three digits precision (behind the comma), and of size up to 14(x14)
with two digits. This can give you an idea of how **mcl** operates,
and what the effect of pruning is.
Use e.g. **-S** **6** for such
a small graph and view the MCL matrix iterands with **--show**.

**--write-binary** (*output format*)

Write matrix dump output in binary mcl format rather
than interchange mcl format (the default). Note that mcxconvert
can be used to convert each one into the other.
See mcxio and mcxconvert for more information.

**-sort** str (*sort mode*)

*str* can be one of **lex**, **size**, **revsize**,
or **none**. The default is 'revsize', in which the largest
clusters come first. If the mode is 'size', smallest clusters
come first, if the mode is 'lex', clusters are ordered
lexicographically, and if the mode is 'none', the order
is the same as produced by the procedure used by mcl to
map matrices onto clusterings.

**-overlap** str (*overlap mode*)

Mode *keep* causes mcl to retain overlap should this improbable event
occur. In theory, **mcl** may generate a clustering that contains overlap,
although this almost never happens in practice, as it requires some
particular type of symmetry to be present in the input graph (not just any
symmetry will do). Mathematically speaking, this is a conjecture and not a
theorem, but the present author wil eat his shoe if it fails to be true (for
marzipan values of shoe). It is easy though to construct an input graph for
which certain mcl settings result in overlap - for example a line graph on
an odd number of nodes. The default is to excise overlapping parts and
introduce them as clusters in their own right. It is possible to allocate
nodes in overlap to the first cluster in which they occur (i.e. rather
arbitrarily), corresponding with mode *cut*.

In mode *split* mcl will put all nodes in overlap into
separate clusters. These clusters are chosen such that
two nodes are put in the same new cluster if and only if
they always occur paired in the clusters of the
overlapping clustering.

This option has more than theoretical use because **mcl** is able
to generate clusterings associated with intermediate iterands.
For these clusterings, overlap is more than a theoretical
possibility, and will often occur. If you specify
the **-L** *k* option, mcl will output the
clustering associated with the last iterand computed, and
it may well contain overlap.

This option has no effect on the clusterings that are
output when using **-dump** *cls* -
the default for those is that overlap is not touched,
and this default can not yet be overridden.

**--force-connected**=y/n (*analyze components*)

**--check-connected**=y/n (*analyze components*)

If the input graph has strong bipartite characteristics,
mcl may yield clusters that do not correspond to connected
components in the input graph. Turn one of these modes on to
analyze the resultant clustering.

If loose clusters are found
they will be split into subclusters corresponding to
connected components.
With **--force-connected**=*y* mcl will write the
corrected clustering to the normal output file, and the old clustering
to the same file with suffix orig.
With **--check-connected**=*y* mcl will write the
loose clustering to the normal output file, and the corrected clustering
to the same file with suffix coco.

These options are not on by default, as the analysis
is currently (overly) time-consuming
and mcl's behaviour actually makes some sense
(when taking bipartite characteristics into account).

**--output-limit**=y/n (*write limit matrix*)

This will write the limit matrix to a file named
**base**-limit.

**--analyze**=y/n (*performance criteria*)

With this mode turned on, **mcl** will reread the input matrix
and compute a few performance criteria and attach them to
the output file. Off by default.

**--append-log**=y/n (*append log*)

Appends a log with the process characteristics to the output file.
Off by default.

**--show-log**=y/n (*show log*)

Shows the log with process characteristics on STDOUT.
By default, this mode is off.

**-ceil-nb** N (*preprune count*)

The nodes of the input graph are considered in descending order of the
number of neighbours they posses. The first nodes has it neighbours of
lowest edge weight removed until no more than **N** remain. The
corresponding reciprocal edges from nodes further down the list are removed
as well. Subsequent nodes are treated in the same way until all nodes in
the graph have at most **N** neighbours. This may be considered a poor
man's hub removal.

**--jury-charter** (*explains jury*)

Explains how the jury synopsis is computed from the jury marks.

**--version** (*show version*)

Show version.

**-how-much-ram** n (*RAM upper bound*)

**n** is interpreted as the number of nodes of an input graph.
mcl will print the maximum amount of RAM it needs for its computations.
The formula for this number in bytes is:

```
   2 * c * k * n

   2  :  two matrices are concurrently held in memory.
   c  :  mcl cell size (as shown by -z).
   n  :  graph cardinality (number of nodes).
   k  :  MAX(s, r).
   s  :  select number (-S, -scheme options).
   r  :  recover number (-R, -scheme options).
```

This estimate will usually be too pessimistic. It does assume though that
the average node degree of the input graph does not exceed k. The
**-how-much-ram** option takes other command-line arguments into
account (such as **-S** and **-R**), and it expresses the
amount of RAM in megabyte units.

**-h** (*show help*)

Shows a selection of the most important **mcl** options.

**--apropos** (*show help*)

Gives a one-line description for all options.

**-z** (*show settings*)

Show current settings for tunable parameters.
**--show-settings** is a synonym.

## PRUNING OPTIONS

**-p** f (*cutoff*)

**-P** n (*1/cutoff*)

**-S** s (*selection number*)

**-R** r (*recover number*)

**-pct** pct (*recover percentage*)

**-my-scheme** n (*tag custom scheme*)

After computing a new (column stochastic) matrix vector during expansion
(which is matrix multiplication c.q. squaring), the vector is
successively exposed to different pruning strategies. The intent of
pruning is that many small entries are removed while retaining much of
the stochastic mass of the original vector. After pruning, vectors are
rescaled to be stochastic again. MCL iterands are theoretically known to
be sparse in a weighted sense, and this manoever effectively perturbs the
MCL process a little in order to obtain matrices that are genuinely
sparse, thus keeping the computation tractable. An example of monitoring
pruning can be found in the discussion of
**-v** **pruning**
at the end of this section.

**mcl** proceeds as follows. First, entries that are smaller than
*cutoff* are removed, resulting in a vector with at most
*1/cutoff* entries. The cutoff can be supplied either by
**-p**, or as the inverse value by **-P**. The latter is more
intuitive, if your intuition is like mine (and the P stands for precision
or pruning by the way).
The cutoff just described is rigid; it is the same for all vectors. The
**--adapt** option causes the computation of a
cutoff that depends on a vector's homogeneity properties, and this option
may or may not speed up mcl.

Second, if the remaining stochastic mass (i.e. the sum of all remaining
entries) is less than *pct*/100 and the number of remaining
entries is less than *r* (as specified by the **-R** flag),
**mcl** will try to regain ground by recovering the largest discarded
entries. The total number of entries is not allowed to grow larger than
*r*.
If recovery was not necessary, mcl tries to prune the vector further
down to at most *s* entries (if applicable), as specified by the
**-S** flag. If this results in a vector that satisfies the recovery
condition then recovery is attempted, exactly as described above. The
latter will not occur of course if *r* <= *s*.

The default setting is something like **-P** **4000** **-S** **500**
**-R** **600**. Check the **-z** flag to be sure. There is a set
of precomposed settings, which can be triggered with the
**-scheme** *k* option. *k*=4 is the default
scheme; higher values for *k* result in costlier and more accurate
computations (vice versa for lower, cheaper, and less accurate).
The schemes are listed using the **--show-schemes** option. It is
advisable to use the **-scheme** option only in interactive mode,
and to use the explicit expressions when doing batch processing. The
reason is that there is *no guarantee whatsoever* that the schemes
will not change between different releases. This is because the scheme
options should reflect good general purpose settings, and it may become
appararent that other schemes are better.

Note that 'less accurate' or 'more accurate' computations may still
generate the same output clusterings. Use **clm dist** to compare output
clusterings for different resource parameters. Refer to clm dist
for a discussion of this issue.

The **-my-scheme** **n** option sets a tag that is used in constructing
the default output naming file. If not used, **mcl** will create
a relatively long string describing the settings of the
**-P**, **-pct**, **-R**, and **-S** parameters,
e.g. **P600Q85R1000S1200** (where **Q** tags the *pct* setting).
If used, mcl will simply use the tag **s***n*.

**-warn-pct** k (*prune warn percentage*)

**-warn-factor** k (*prune warn factor*)

The two options **-warn-pct** and **-warn-factor** relate to
warnings that may be triggered once the *initial* pruning of a vector
is completed. The idea is to issue warnings if initial pruning almost
completely destroys a computed vector, as this may be a sign that the
pruning parameters should be changed. It depends on the mass remaining
after initial pruning whether a warning will be issued. If that mass is
less than *warn-pct* or if the number of remaining entries is smaller
by a factor *warn-factor* than both the number of entries originally
computed *and* the recovery number, in that case, **mcl** will issue a
warning.

**-warn-pct** takes an integer between 0 and 100 as parameter,
**-warn-factor** takes a real positive number. They default to
something like 30 and 50.0. If you want to see less warnings, decrease
*warn-pct* and increase *warn-factor*. Set *warn-factor* to zero
if you want no warnings.

**-v** **pruning**

Pruning verbosity mode causes **mcl** to emit several statistics related to
the pruning process, each of which is described below. Use
**-v** **explain** to get explanatory headers in the output as well
(or simply use **-v** **all**).

**Selection and recovery**  
The number of selections and recoveries **mcl** had to perform during each
iteration is shown. It also shows the number of vectors for which the
mass after final pruning was below the fraction defined by the
**-pct** option as a percentage (default probably 90
or 95).

**Initial and pruned vector footprint distributions**  
The distribution of the vector footprints (i.e. the number of nonzero
entries) before and after pruning is shown. This is assembled in a terse
(horrid if you will) format, looking as follows (with some context
stripped, noting that the data for three expansion steps is shown):

```
----------------------------------------------------
 mass percentages  | distr of vec footprints       |
         |         |____ expand ___.____ prune ____|
  prune  | final   |e4   e3   e2   |e4  e3   e2    |
all ny nx|all ny nx|8532c8532c8532c|8532c8532c8532c|
---------.---------.---------------.---------.-----.
 98 88 86  98 91 86 _________022456 ___________0234
 98 89 86  98 94 91 _______00245678 ___________0234
 98 90 89  99 95 94 _______00235568 ___________0234
 ...
```

This particular output was generated (and truncated after three rounds of
expansion and inflation) from clustering a protein graph on 9058 nodes
with settings **-I** **1.4**, **-P** **2000**, **-S** **500**,
**-R** **600**, and **-pct** **95**.

The header entries 8532c85.. indicate thresholds going from 80000, 50000,
20000, 12500, 8000, all the way down to 300, 200, and 125. The character 'c'
signifies the base 1.25 (for no apparent reason). The second entry '2'
(after '0') on the first line signifies that roughly 20 percent of all the
vectors had a footprint (#nonzero entries) in excess of 800 entries.
Likewise, 40 percent had a footprint in excess of 300 entries. The '0'
entries signify a fraction somewhere below 5 percent, and the '@' entries
signify a fraction somewhere above 95 percent.

Two columns are listed, one for the expansion vector footprints
(i.e. after squaring), and the other for the vector
footprints *right after initial pruning took place* (i.e. before
selection and recovery, after either adaptive or rigid pruning).
This may give an idea of the soundness of the initial pruning
process (overly severe, or overly mild), and the extent
to which you want to apply selection and/or recovery.

**Initial and final mass windows**  
The mass averages of the pruned vectors after the first selection
stage are shown, and the mass averages of the vectors as *finally
computed*, i.e. after selection and recovery. Note that the latter
corresponds to a different stage than what is shown for the vector
footprints, if either selection or recovery is turned on.
For both cases, three averages are shown: the average over all vectors,
the average over the worst x cases, and the average over the worst y
cases. The mass averages are shown as percentages: '98' on the first
line under the 'prune/all' column means that overall 98 percent of the
stochastic mass of the matrix was kept after pruning.

This example demonstrates that many entries could be
removed while retaining much of the stochastic mass. The effect of the
recovery (**-R**) parameter is also clear: the final averages are
higher than the initial averages, as a result of **mcl** undoing some
overenthousiastic pruning.

An average over the worst k cases is called a window of width k;
internally, **mcl** tracks many more such windows. The result of this can
be seen when using the **--append-log**=**y** option
(which appends a log to the cluster output) or the
**--show-log**=**y** option
(which sends the log to STDOUT).
From a fixed set of windows those that are applicable are tracked, that
is, all those windows for which the width does not exceed the graph
cardinality. The windows in the fixed set have respective sizes 1, 2, 5,
10, 20, 50, and so on up until 5000000 (which makes 15 windows in all).

## PIPELINES

As of the 1.006 release, label data can be directly streamed into
MCL as described in GETTING STARTED and EXAMPLES.
For BLAST input this is achieved
by hooking up mcxdeblast **--abc-out**=*-* with
**mcl** - **--abc**. Refer to GETTING STARTED for examples.

The classic mode of operation is more heavyweight, and the remainder
of this section describes the underlying design.
Much of the code now used for streaming directly into mcl was
derived from this earlier framework.

In general, clustering requires several stages; creating the matrix,
running mcl, and displaying the result. The display stage is supported
by **clm format**. The matrix creation stage often needs only be
done once for a given data collection, followed by repeated runs of
the other two stages for varying inflation values and scheme settings.

The matrix creation stage can often be split up in two more stages,
namely parsing a data file in some given format, and assembling a
matrix from the data bits and pieces, such as node indices and edge
weights or even edge weight contributions.
The assembly step can be done by mcxassemble,
which allows a very general input format and customizable behaviour
in how the bits and pieces should be transformed to the input graph.
This leaves the parse stage to be filled in.

The mclpipeline script implements a generic and
customizable pipeline encapsulating the four stages distinguished
here (parsing, assembly, clustering, display). It is possible to
let only part of the pipeline be active, and many other features are
supported. The IO mechanism is entirely file based, and files are
associated with parametrizations via file name extensions (by all
means a simple mechanism).

mclpipeline requires a single parse script to be specified.
It will be plugged into the pipeline and you should be set to run.
The parse script must satisfy the interface requirements described
in the mclpipeline manual page.

For BLAST input, the mclblastline script
provides a dedicated mclpipeline interface. It uses the
mcxdeblast script that comes prepackaged with mcl.

## EXAMPLES

The following is an example of label input

```
---8<------8<------8<------8<------8<---
cat hat  0.2
hat bat  0.16
bat cat  1.0
bat bit  0.125
bit fit  0.25
fit hit  0.5
hit bit  0.16
--->8------>8------>8------>8------>8---
```

It can be clustered like this:

```
mcl cathat --abc -o out.cathat
```

The file out.cathat should now like like this

```
---8<------8<------8<------8<------8<---
cat hat bat
bit fit hit
--->8------>8------>8------>8------>8---
```

A few things to note. First, MCL will symmetrize any
arrow it finds. If it sees bat cat 1.0 it will act as if it also
saw cat bat 1.0. You can explicitly specify cat bat 1.0,
mcl will in the first parse stage simply end up with duplicate
entries. Second, MCL deduplicates repeated edges by taking the
one with the maximum value. So,

```
---8<------8<------8<------8<------8<---
cat hat  0.2
hat cat  0.16
hat cat  0.8
--->8------>8------>8------>8------>8---
```

Will result in two arrows cat-hat and hat-cat both
with value 0.8.

## APPLICABILITY

**mcl** will work very well for graphs in which the diameter of the natural
clusters is not too large. The presence of many edges between different
clusters is not problematic; as long as there is cluster structure, mcl
will find it. It is less likely to work well for graphs with clusters
(inducing subgraphs) of large diameter, e.g. grid-like graphs derived from
Euclidean data. So mcl in its canonical form is certainly not fit for
boundary detection or image segmentation. I experimented with a modified
mcl and boundary detection in the thesis pointed to below (see
REFERENCES). This was fun and not entirely unsuccesful, but not
something to be pursued further.

**mcl** likes *undirected input graphs best*, and it really dislikes graphs
with node pairs (i,j) for which an arc going from i to j is present and the
counter-arc from j to i is absent. Try to make your input graph undirected.
Furthermore, mcl interprets edge weights in graphs as similarities. If you
are used to working with dissimilarities, you will have to convert those to
similarities using some conversion formula. The most important thing is
that you feel confident that the similarities are reasonable, i.e. if X is
similar to Y with weight 2, and X is similar to Z with weight 200, then this
should mean that the similarity of Y (to X) is neglectible compared with the
similarity of Z (to X).

**mcl** is probably not suited for clustering *tree graphs*. This is because
mcl works best if there are multiple paths between different nodes in the
natural clusters, but in tree graphs there is only one path between any pair
of nodes. Trees are too sparse a structure for mcl to work on.

**mcl** may well be suited for clustering *lattices*. It will depend
on the density characteristics of the lattice, and the conditions for
success are the same as those for clustering graphs in general: The
diameter of the natural clusters should not be too large.
**NOTE** when clustering a lattice, you *have* to cluster
the underlying undirected graph, and not the directed graph that represents
the lattice itself. The reason is that one has to allow mcl (or any other
cluster algorithm) to 'look back in time', so to speak. Clustering and
directionality bite each other (long discussion omitted).

**mcl** has a worst-case time complexity O(N\*k^2), where N is the number of
nodes in the graph, and k is the maximum number of neighbours tracked during
computations. k depends on the **-P** and **-S** options. If the
**-S** option is used (which is the default setting) then k equals the
value corresponding with this option. Typical values for k are in the range
500..1000. The average case is much better than the worst case though, as
cluster structure itself has the effect of helping mcl's pruning schemes,
certainly if the diameter of natural clusters is not large.

## FILES

There are currently no resource nor configuration files.
The mcl matrix format is described in the mcxio section.

## ENVIRONMENT

MCLXIODIGITS

When writing matrices in interchange format, mcl will use this variable (if
present) as the precision (number of digits) for printing the fractional
part of values.

MCLXIOVERBOSITY

MCL and its sibling applications will usually report about matrix
input/output from/to disk. The verbosity level can be regulated
via MCLXIOVERBOSITY. These are the levels it can currently be set to.

1

Silent but applications may alter this.

2

Silent and applications can not alter this.

4

Verbose but applications may alter this.

8

Verbose and applications can not alter this (default).

MCLXIOFORMAT

MCL and its sibling applications will by default output matrices
in interchange format rather than binary format (cf. mcxio).
The desired format can be controlled via the variable
MCLXIOFORMAT. These are the levels it can currently be set to.

1

Interchange format but applications may alter this.

2

Interchange format and applications can not alter this (default).

4

Binary format but applications may alter this.

8

Binary format and applications can not alter this.

MCLXICFLAGS

If matrices are output in interchange format, by default empty vectors
will not be listed. Equivalently (during input time),
vectors for which no listing is present are understood to be empty -
note that the *presence* of a vector is established using
the domain information found in the header part.
It is possible to enforce listing of empty vectors by
setting bit '1' in the variable MCLXICFLAGS.

MCLXIOUNCHECKED

MCL and its sibling applications will always check a matrix for consistency
while it is being read. If this variable is set, the consistency check is
omitted. For large graphs the speed up can be considerable. However, if the
input graph is not conforming it will likely crash the application that
is using it.

## DIAGNOSTICS

If **mcl** issues a diagnostic error, it will most likely be
because the input matrix could not be parsed succesfully.
**mcl** tries to be helpful in describing the kind of parse error.
The mcl matrix format is described in the mcxio section.

## BUGS

No known bugs at this time.

## AUTHOR

Stijn van Dongen.

## HISTORY/CREDITS

The MCL algorithm was conceived in spring 1996 by the present author.
The first implementation of the MCL algorithm followed that spring
and summer. It was written in Perl and proved the viability of
the algorithm. The implementation described here began its life in
autumn 1997. The first versions of the vital matrix library
were designed jointly by Stijn van Dongen and Annius Groenink in
the period Oktober 1997 - May 1999. The efficient matrix-vector
multiplication routine was written by Annius. This routine is
without significant changes still one of the cornerstones of this
MCL implementation.

Since May 1999 all MCL libraries have seen much development and
redesign by the present author. Matrix-matrix multiplication has been
rewritten several times to take full advantage of the sparseness
properties of the stochastic matrices brought forth by the MCL
algorithm. This mostly concerns the issue of pruning — removal of
small elements in a stochastic column in order to keep matrices
sparse.

Very instructive was that around April 2001 Rob Koopman pointed out
that selecting the k largest elements out of a collection of n is
best done using a min-heap. This was the key to the second major
rewrite (now counting three) of the MCL pruning schemes, resulting in
much faster code, generally producing a more accurate computation of
the MCL process.

In May 2001 Anton Enright initiated the parallellization of the
**mcl** code and threaded inflation. From this example, Stijn threaded
expansion. This was great, as the MCL data structures and operands
(normal matrix multiplication and Hadamard multiplication) just beg
for parallellization.

Onwards.
The January 2003 03-010 release introduced support for sparsely
enumerated (i.e. indices need not be sequential) graphs and matrices, the
result of a major overhaul of the matrix library and most higher layers.
Conceptually, the library now sees matrices as infinite quadrants
of which only finite subsections happen to have nonzero entries.

The June 2003 03-154 release introduced unix-type pipelines for clustering,
including the BLAST parser mcxdeblast and the mclblastline script.
The April 2004 04-105 release revived binary format, which has been a first
class citizen every since.

With the March 2005 05-090 release mcxsubs finally acquired a sane
specification syntax. The November 2005 05-314 release brought the ability
to stream label input directly into mcl. The subsequent release introduced a
transformation language shared by various mcl siblings that allows arbitrary
progressions of transformations to be applied to either stream values or
matrix values.

Joost van Baal set up the mcl CVS tree and packaged mcl for Debian
GNU/Linux. He completely autotooled the sources, so much so that at first I
found it hard to find them back amidst bootstrap, aclocal.m4, depcomp, and
other beauties.

Jan van der Steen shared his elegant mempool code. Philip Lijnzaad gave
useful comments. Philip, Shawn Hoon, Abel Ureta-Vidal,
and Martin Mokrejs sent helpful bug reports.

Abel Ureta-Vidal and Dinakarpandian Deendayal commented on
and contributed to mcxdeblast and mcxassemble.

Tim Hughes contributed several good bug reports for mcxassemble,
mcxdeblast and zoem (a workhorse for **clm format**).

## SEE ALSO

mclfaq - Frequently Asked Questions.

mcxio - a description of the mcl matrix format.

There are many more utilities. Consult
mclfamily for an overview of and links to all the documentation
and the utilities in the mcl family.

**minimcl** is a 200-line perl implementation of mcl. It is shipped
in the mcl distribution and can be found online at
http://micans.org/mcl.

mcl's home at http://micans.org/mcl/.

## REFERENCES

[1]
Stijn van Dongen, *Graph Clustering by Flow Simulation*.
PhD thesis, University of Utrecht, May 2000.  
http://www.library.uu.nl/digiarchief/dip/diss/1895620/inhoud.htm

[2]
Stijn van Dongen, *Graph Clustering Via a Discrete Uncoupling Process*,
SIAM Journal on Matrix Analysis and Applications, 30(1):121-141, 2008.
http://link.aip.org/link/?SJMAEL/30/121/1

[3]
Stijn van Dongen. *A cluster algorithm for graphs*.
Technical Report INS-R0010, National Research Institute for Mathematics and
Computer Science in the Netherlands, Amsterdam, May 2000.  
http://www.cwi.nl/ftp/CWIreports/INS/INS-R0010.ps.Z

[4]
Stijn van Dongen. *A stochastic uncoupling process for graphs*.
Technical Report INS-R0011, National Research Institute for Mathematics and
Computer Science in the Netherlands, Amsterdam, May 2000.  
http://www.cwi.nl/ftp/CWIreports/INS/INS-R0011.ps.Z

[5]
Stijn van Dongen. *Performance criteria for graph clustering and Markov
cluster experiments*. Technical Report INS-R0012, National Research
Institute for Mathematics and Computer Science in the Netherlands,
Amsterdam, May 2000.  
http://www.cwi.nl/ftp/CWIreports/INS/INS-R0012.ps.Z

[6]
Enright A.J., Van Dongen S., Ouzounis C.A.
*An efficient algorithm for large-scale detection of protein families*,
Nucleic Acids Research 30(7):1575-1584 (2002).

## NOTES

This page was generated from **ZOEM** manual macros,
http://micans.org/zoem. Both html and roff pages can be created
from the same source without having to bother with all the usual conversion
problems, while keeping some level of sophistication in the typesetting.
This is the PostScript derived from the zoem troff
output.
