# Supplementary material for: MCL-CAw: a refinement of MCL for detecting yeast complexes from weighted PPI networks by incorporating core-attachment structure
Source: BMC Bioinformatics. 2010 Oct 12;11:504. doi: 10.1186/1471-2105-11-504 (PMC2965181; doi:10.1186/1471-2105-11-504)
Supplement: Additional files 2 — The MCL-CAw software package: The source code and installation details for the MCL-CAw software. [file 1471-2105-11-504-S2.ZIP › Additional_files_2/mcl-09-149.tar.gz!/mcl-09-149/doc/mclcm.html]

The mclcm manual


29 May 2009   
**mclcm**
1.008, 09-149

1.

NAME

2.

SYNOPSIS

3.

DESCRIPTION

4.

OPTIONS

5.

AUTHOR

6.

SEE ALSO

## NAME

mclcm — hierarchical clustering of graphs with mcl

## SYNOPSIS

**mclcm** <-|fname> [mclcm-options] [-- "mcl options"\*]

```
mclcm <-|fname> -a "-I 4 --shadow=vl"
mclcm <-|fname> -a "-I 3" -- "-I 5"
mclcm <-|fname> -a "-I 3" -b1 "" -- "-ph 3 --shadow=vl -I 5"
```

**mclcm** <-fname>
**[--contract** (*contraction mode*)**]**
**[--dispatch** (*dispatch mode*)**]**
**[--integrate** (*integrate mode*)**]**
**[--subcluster** (*subcluster mode*)**]**
**[-a** <opts> (*shared mcl options*)**]**
**[-b1** <opts> (*dedicated base 1 mcl options*)**]**
**[-b2** <opts> (*dedicated base 2 mcl options*)**]**
**[-c** <fname> (*input clustering*)**]**
**[-n** <num> (*iteration limit*)**]**
**[--root** (*ensure universe root clustering*)**]**
**[-cone** <fname> (*nested cluster stack file*)**]**
**[-stack** <fname> (*expanded cluster stack file*)**]**
**[-coarse** <fname> (*coarsened graphs file*)**]**
**[-write** (*stack,cone,coarse,steps*)**]**
**[-write-base** <fname> (*write base matrix*)**]**
**[-stem** <str> (*prefix for all outputs*)**]**
**[--mplex=**y/n (*write clusterings separately*)**]**
**[-annot** str (*dummy annotation option*)**]**
**[-h** (*print synopsis, exit*)**]**
**[--apropos** (*print synopsis, exit*)**]**
**[--version** (*print version, exit*)**]**
**[-q** spec (*log levels*)**]**
**[-z** (*show default shared options*)**]**
[-- "mcl options"\*]

## DESCRIPTION

The mclcm options may be followed by a number of trailing arguments.
The trailing arguments should be separated from the mclcm options
by the separator --.
Normally each trailing argument should consist of a set of zero, one, or more mcl arguments
enclosed in quotes or double quotes to group them together.
These arguments are passed to the successive stages of hierarchical
clustering. They are combined with the default options. If an option
is specified both in the default options list and in a trailing
options list the latter specification overrides the former.
When the **--integrate** option is specified the trailing arguments
must be names of files containing mcl clusterings; see further below.
**mclcm** has four major modes of operation, namely *contraction* (default),
*integration*, *dispatch*, and *subcluster*. Each mode is
described a little below. Note though that *dispatch* mode is not
the best mode to use for hierarchical clustering. It is mostly useful
to generate multiple mcl clusterings in a single run.

In all modes **mclcm** will generate a file, by default called *mcl.cone*.
This is a representation of a hierarchical clustering that is particular
to mcl. It can be converted to *newick* format like this:

```
         mcxdump -imx-tree mcl.cone --newick -o NEWICKFILE
   OR    mcxdump -imx-tree mcl.cone --newick -o NEWICKFILE -tab TABFILE
```

In the last example, TABFILE should be a file containing a mapping
from mcl labels to application labels. Refer to mcxio for
more information about tab files and mcl input/ouput formats.

## OPTIONS

**--contract** (*repeated contraction mode*)

This is the default mode of operation.
At each successive step of constructing the hierarchy on top of the first
level mcl clustering, mclcm uses a matrix derived from the input matrix and
the last computed clustering to compute a contracted graph.
The contracted graph is a graph where the nodes represent the clusters of
the last clustering. The matrix derived from the input graph that is used to
construct the contracted graph is called the *base matrix*. The base
matrix can be either the *start matrix* or the *expansion matrix*.
The *start matrix* is the input matrix after transformations have been
applied to it (if any).
The *expansion matrix* is the first expanded matrix of some
mcl process applied to the input graph.

By default the base matrix is constructed from either the start matrix
or the expansion matrix obtained from the first mcl process.
It is possible to use a start matrix derived from special purpose
mcl transformation parameters (such as **-ph** and **-tf**)
or an expansion matrix derived from a special purpose mcl process.
The **-b1** and **-b2** parameters provide the interfaces
to this functionality.

You are advised to start with a high inflation value for the input
graph and to use shadowing, e.g. include --shadow=vl in the
**-a** argument.
This generally leads to hierarchies that are better balanced.
Shadowing is a transformation where nodes are added to the
graph, preventing relatively distant nodes from unwanted chaining.
For more information refer to the **mcl** manual.
The invocations in SYNOPSIS are a good starting point.

**--dispatch** (*different mcl processes*)

In this mode each trailing argument is specified as a set of options to pass
to an mcl process. For each trailing argument an mcl process is thus
computed. The set of resulting clusterings is integrated into a hierarchy.

**--integrate** (*existing clusterings*)

This mode is similar to *dispatch* mode. The difference is that with
this option mclcm simply integrates a set of already existing clusterings.
Each trailing argument must be the name of a file containing a clustering.
The set of clusterings thus specified is integrated into a hierarchy.

**--subcluster** (*repeated sub-clustering*)

In this mode each trailing argument specifies a set of options to pass to an
mcl process. The second clustering process is applied to the graph of
components induced by the first clustering, resulting in a further
subdivision of the first clustering. This approach is repeated with each
further trailing argument. With this approach, the first clustering will be
the most coarse clustering. Hence, subsequent trailing arguments will
typically specify increasingly higher inflation values, pre-inflation
values, and optionally more stringent transformation parameters
in order to achieve further subdivsions.

**-a** <opts> (*shared mcl options*)

Use this to change and/or set the default mcl options for
all iterations. Use quotes if necessary.
Example of usage: -a "-I 5".

**-b1** <opts> (*dedicated base 1 mcl options*)

This will apply the mcl options *opts* to the input matrix. The
resulting start matrix is used as the base matrix for constucting contracted
graphs.

**-b2** <opts> (*dedicated base 2 mcl options*)

This will apply the mcl options *opts* to the input matrix and
compute the first iterand of the corresponding mcl process. The first
iterand, aka the expansion matrix, is used as the base matrix for
constucting contracted graphs.

**-c** <fname> (*input clustering*)

The hierarchical clustering process will be kicked off by
the clustering found in *<fname>*.

**-n** <num> (*iteration limit*)

This puts an upper bound to the number of contractions that
will be performed.

**--root** (*ensure universe root clustering*)

In case the graph consists of different connected components,
the last clustering computed by the mclcm process will
correspond with those connected components. This option
simply adds an artificial clustering where all nodes
have been joined into a single cluster.

**-cone** <fname> (*nested cluster stack file*)

File to write the nested cluster stack to.
The nested cluster stack contains a sequence of clusterings,
each written as an MCL matrix.
The first (bottom) clustering is a clustering of the nodes
in the input graph. Each subsequent clustering is a clustering
where the nodes are the clusters of the previous clustering.
**mcxdump** can dump this format if the file name is given as
the **-imx-stack** option. The explanation
for the cone/stack discrepancy is simple. To mcxdump
the contents are simply a stack of matrices. It does not
care whether the stack is cone shaped, cylindrical, or
yet another shape.

**-stack** <fname> (*expanded cluster stack file*)

File to write the expanded cluster stack to. The expanded cluster stack is
similar to the nested cluster stack except that each cluster lists all the
nodes in the input graph it contains.
**mcxdump** can dump this format if the file name is given as
the **-imx-stack** option.

**-coarse** <fname> (*coarsened graphs file*)

File to write the sequence of coarsened graphs to. Each clustering induces a
coarsened graph where the nodes represent the clusters and an edge between
two nodes represents the connectivity between the corresponding two
clusters. The computation of this connectivity takes into account all edges
between the two clusters in in the original graph.

**-write** <tag> (*select output modes*)

Use this option to explictly specify all of the output types you want
written in a comma-separated string. *<tag>* may contain
any of the strings *stack*, *cone*, *coarse*, *steps*.
The current default is to write all of these except *coarse*.
The latter dumps the intermediate coarsened (aka contracted) graphs
to a single file.

**-write-base** <fname> (*write base matrix*)

Write the base matrix to file. This can be useful for debugging
expectations.

**-stem** <str> (*prefix for all outputs*)

All output files share the same prefix. The default is mcl
and can be changed with this option.

**--mplex**=y/n (*write clusterings separately*)

If turned on each clustering is written in a separate file. The first
clustering is written to the file *<stem>*.3 where *<stem>* is
determined by the **-stem** option. For each subsequent clustering
the index is incremented by two, so clusterings are written to
files for which the name ends with an odd index.

**-annot** str (*dummy annotation option*)

**mclcm** writes the command line with which it was invoked to the output file
(either of the *cone* or *stack* files). Use this option to include
any additional information. mclcm does nothing with this option except copying
it as just described.

**-q** spec (*log levels*)

Set the quiet level. Read tingea.log for syntax and semantics.

**-z** (*show default shared options*)

Show the default mcl options. These are used for each mcl invocation as
successively applied to the input graph and succeeding contracted graphs.

## AUTHOR

Stijn van Dongen.

## SEE ALSO

mclfamily for an overview of all the documentation
and the utilities in the mcl family.
