# Supplementary material for: MCL-CAw: a refinement of MCL for detecting yeast complexes from weighted PPI networks by incorporating core-attachment structure
Source: BMC Bioinformatics. 2010 Oct 12;11:504. doi: 10.1186/1471-2105-11-504 (PMC2965181; doi:10.1186/1471-2105-11-504)
Supplement: Additional files 2 — The MCL-CAw software package: The source code and installation details for the MCL-CAw software. [file 1471-2105-11-504-S2.ZIP › Additional_files_2/mcl-09-149.tar.gz!/mcl-09-149/doc/mclfamily.html]

The MCL family


29 May 2009   
**mcl**
1.008, 09-149

1.

NAME

2.

DESCRIPTION

## NAME

mclfamily — a description of the mcl family of cluster applications.

**mcl** is an implementation of the Markov Cluster Algorithm, aka MCL algoritm.
It is described in the mcl manual.
Several other utilities are part of the MCL distribution. This manual pages
gives an overview.

mcl

the cluster algorithm

mclfaq

MCL Frequently Asked Questions

mcxio

the graph/matrix input/output format

mcx

general graph/matrix tasks

mcx convert

convert between interchange/binary storage types

mcx diameter

compute diameter of a symmetric graph

mcx clcf

compute clustering coefficient

mcx ctty

compute betweenness centrality

mcx erdos

compute shortest paths in graphs

mcx q

compute simple graph statistics

mcxassemble

create matrices from raw data

mcxdump

dump a matrix optionally with label substitions

mcxload

load label data into matrix and tab files

mcxarray

transform array data to MCL matrices

mcxmap

relabel indices in a graph/matrix

mcxrand

randomly remove, add, and perturb edge weights

mcxsubs

extracting submatrices in various ways

mcxi

general matrix operations

clm

general graph-clustering related tasks

clm dist

compute split/join distance between clusterings

clm vol

derive node volatility scores from a set of clusterings

clm info

compute performance measure for clusterings

clm meet

compute intersection of clusterings

clm mate

find best matching clusters between clusterings

clm close

fetch connected components from graphs or subgraphs

clm imac

interpret MCL iterand/matrix as clustering

clm residue

extend subgraph clustering

clm order

reorder indices to represent blocks from different clusterings

clmformat

display clusters as html or txt files

mclpipeline

parsing/assembly/clustering/display

mclblastline \*

BLAST pipeline

mcxdeblast \*

parse BLAST files

Entries marked \* are not available if only a default
install is done.

## DESCRIPTION

mcl - the clustering program. Since the 05-314 release
it has the ability to read in and cluster label input.

mclfamily - Frequently Asked Questions.

mcxio - a description of the mcl matrix format.

mcxassemble - assemble a matrix/graph from partial edge
weight scores. Useful intermediate format to be used when transforming
application specific data into an mcl input matrix.
However, **mcl** has now acquired the ability to read graphs directly
from label input, removing the need for **mcxassemble** in most cases.

mcxdump - dump matrices in a line-based format, optionally map
indices to labels. Either a node pair (matrix entry) or a node list
(matrix row) is output per line.

mcxload - load matrices and tab files
from a line-based ID1 ID2 format.
It can load bipartite structures in which the two columns
contain labels from different domains. It has many options
to further symmetrize and transform the input.

mcxarray - transform array data to MCL matrices. The data
may be of rectangular M x N type. Either an M x M
or an N x N dimensional matrix can be made, by computing
correlation scores between the vectors in one of the to domains.
The Pearson correlation coefficient and the cosine are supported,
and further tearing and pruning options can be applied.

mcxmap - relabel indices in a graph.

mcxrand - randomly remove, add, and perturb edge weights.

mcx convert - convert matrices from interchange mcl format to
binary mcl format or vice versa.

mcx diameter - compute the diameter of a graph.

mcx clcf - compute the clustering coefficient of a graph.

mcxi - an interpreter for a stack language that enables
interaction with the **mcl** matrix libraries. It can be used both from the
command line and interactively, and supports a rich set of operations such
as transposition, scaling, column scaling, multiplication, Hadamard powers
and products, et cetera. The general aim is to provide handles for simple
number and matrix arithmetic,
and for graph, set, and clustering operations. The following is
a very simple example of implementing and using **mcl** in this language.

```
 2.0 .i def                    # define inflation value.
 /small lm                     # load matrix in file 'small'.
 dim id add                    # add identity matrix.
 st .x def                     # make stochastic, bind to x.
 { xpn .i infl vm } .mcl def   # define one mcl iteration.
 20 .x .mcl repeat             # iterate  20 times
 imac                          # interpret matrix as clustering.
 vm                            # view matrix (clustering).
```

One of the more interesting things that can be done is doing mcl runs
with more complicated inflation profiles than the two-constant approach used
in **mcl** itself.

mcxsubs - compute a submatrix of a given matrix, where row
and column index sets can be specified as lists of indices combined
with list of clusters in a given clustering. Useful for inspecting
local cluster structure.

clm dist - compute the split/join distance between two
partitions. The split/join distance is better suited for measuring partition
similarity than the long-known equivalence mismatch coefficient. The former
measures the number of node moves required to transform one partition into
the other, the latter measures differences between volumes of edges of
unions of complete graphs associated with partitions.

clm info - compute a performance measure saying how well
a clustering captures the edge weights of the input graph. Useful
for comparing different clusterings on the same graph, best used in
conjunction with clm dist - because comparing clusterings at
different levels of granularity should somewhat change the performance
interpretation. The latter issue is discussed in the clm dist
entry.

clm meet - compute the intersection of a set of clusterings,
i.e. the largest clustering that is a subclustering of all. Useful
for measuring the consistency of a set of different clusterings
at supposedly different levels of granularity (in conjunction with clm dist).

clm mate - find best matching clusters between two different
clusterings.

clm close - fetch connected components from graphs or subgraphs.

clm imac - interpret MCL iterands as clusterings. The clusterings
associated with early iterands may contain overlap, should you be
interested therein.

clm residue - extend a clustering of a subgraph onto a clustering
of the larger graph.

clmformat - display clusters suitable for scrutinizing.

clm order - reorder indices to represent blocks from
different clusterings.

mclpipeline - set up a pipeline from data parsing stage
unto clustering format/display stage.

mclblastline - BLAST specific pipeline.

mcxdeblast - BLAST parser. Can be used to directly stream
a graph into **mcl**. Can also prepare input for mcxassemble
or can be plugged into the heavy-weight mclblastline.
