# Supplementary material for: MCL-CAw: a refinement of MCL for detecting yeast complexes from weighted PPI networks by incorporating core-attachment structure
Source: BMC Bioinformatics. 2010 Oct 12;11:504. doi: 10.1186/1471-2105-11-504 (PMC2965181; doi:10.1186/1471-2105-11-504)
Supplement: Additional files 2 — The MCL-CAw software package: The source code and installation details for the MCL-CAw software. [file 1471-2105-11-504-S2.ZIP › Additional_files_2/mcl-09-149.tar.gz!/mcl-09-149/doc/mcx.html]

The mcx manual


29 May 2009   
**mcx**
1.008, 09-149

1.

NAME

2.

SYNOPSIS

3.

DESCRIPTION

4.

OPTIONS

5.

AUTHOR

6.

SEE ALSO

## NAME

mcx — perform various computations on graphs and matrices

## SYNOPSIS

```
   mcx <mode> [mode-options] [mode-files]
```

## DESCRIPTION

**mcx** implements a variety of computations on graphs and matrices. The
first argument to **mcx** should be a *mode*, which is a string establishing
the type of computation to invoke. Each mode *mode* is described in the
mcx*mode* manual page. The currently available modes are
**convert**, **diameter**, **clcf**, **q** and **ctty**.
The **convert** mode is thus described in the
mcxconvert manual page.

Invoking **mcx** without arguments causes it to print out a list
of available modes with a short description of the type of
command line expected by that mode.

Invoking **mcx** with just a mode will print out a longer listing
of options available for that particular mode.
Modes that are able to function normally without arguments
can be invoked by adding the *--nop* argument.

Several options are shared between all **mcx** modes. Note that
these options are specified after the **mode**. See OPTIONS.

The **--version** causes **mcx** to print out version
and license information.

## OPTIONS

These are options that pertain to all modes. They should be specified
*after* the mode argument.

**-h** (*synopsis*)

**--apropos** (*synopsis*)

List available options.

**--nop** (*no-op*)

Not an option. This option has no effect then to increment
the argument count. This can be useful for **mcx** modes which are able to
function without any options. Such a mode typically reads from STDIN, writes
to STDOUT, and uses default settings. However, simply specifying a mode
without options leads **mcx** to output a list of available options for that
mode. This can be prevented by using the **--nop** option.

**-set** (*key=val*)

Sets the key **key** to value **val** in the environment.
Some modes allow adjustment of settings in this manner.

**-progress** <num> (*progress interval size*)

Defines the interval that defines the progress frequency.

**--debug** (*turn on debugging*)

Turn debugging on.

**--version** (*show version*)

Show version.

## AUTHOR

Stijn van Dongen.

## SEE ALSO

mclfamily for an overview of all the documentation
and the utilities in the mcl family.
