# Supplementary material for: MCL-CAw: a refinement of MCL for detecting yeast complexes from weighted PPI networks by incorporating core-attachment structure
Source: BMC Bioinformatics. 2010 Oct 12;11:504. doi: 10.1186/1471-2105-11-504 (PMC2965181; doi:10.1186/1471-2105-11-504)
Supplement: Additional files 2 — The MCL-CAw software package: The source code and installation details for the MCL-CAw software. [file 1471-2105-11-504-S2.ZIP › Additional_files_2/mcl-09-149.tar.gz!/mcl-09-149/doc/mcxconvert.html]

The mcx convert manual


29 May 2009   
**mcx convert**
1.008, 09-149

1.

NAME

2.

SYNOPSIS

3.

DESCRIPTION

4.

OPTIONS

5.

AUTHOR

6.

SEE ALSO

## NAME

mcx convert — convert between mcx storage types

## SYNOPSIS

**mcx convert** <matrix-file-in> <matrix-file-out>  
**mcx convert** [--write-binary] --cone-to-stack <cat-file-in> <cat-file-out>  
**mcx convert** [--write-binary] --stack-to-cone <cat-file-in> <cat-file-out>

mcxconvert is not in actual fact a program. This manual
page documents the behaviour and options of the mcx program when
invoked in mode *convert*. The options **-h**, **--apropos**,
**--version**, **-set**, **--nop**, **-progress** *<num>*
are accessible
in all **mcx** modes. They are described
in the mcx manual page.

**mcx convert**
**[--cone-to-stack** (*transform cone file to stack file*)**]**
**[--stack-to-cone** (*transform stack file to cone file*)**]**
**[--write-binary** (*output native binary format*)**]**
**[--cat** (*read and write cat format*)**]**
**[-cat-max** <num> (*limit the stack conversion to <num> matrices*)**]**

In the two-argument invocation without additional arguments, **mcx convert**
converts from the format found in the first file to the other format, i.e.
from native interchange to native binary format or the other way around. When
querying with the **-q** option, mcx{convert} will output a one-line
synopsis describing the matrix in the argument. The
**--cone-to-stack** and **--stack-to-cone** options convert
between the two types of concatenated output provided by **mclcm**.

## DESCRIPTION

The **mcl** libraries make extensive use of matrices. Matrices
are used to encode graphs, matrices and clusterings.
They can be stored either in interchange or in binary format. The latter is
somewhat more efficient in storage and much faster in both reading and
writing, but the default is interchange format.

The **mcl** input routines recognize the type of storage they are dealing
with. If you want to convert a matrix to the other storage type,
simply specify the file name of the matrix you want to convert.
**mcx convert** will recognize its type, and write the other type
to the file specified as the second argument.

## OPTIONS

**--cone-to-stack** (*transform cone file to stack file*)

This option requires two trailing options, the names of respectively the
source cone file and the target stack file.

**--stack-to-cone** (*transform stack file to cone file*)

This option requires two trailing options, the names of respectively the
source stack file and the target cone file.

**--cat** (*read and write cat format*)

**-cat-max** <num> (*limit the stack conversion to <num> matrices*)

**--write-binary** (*output native binary format*)

This option is only useful with either of the options **--cone-to-stack**,
**--stack-to-cone**, or **--cat**.

## AUTHOR

Stijn van Dongen.

## SEE ALSO

mcxio,
and mclfamily for an overview of all the documentation
and the utilities in the mcl family.
