# Supplementary material for: MCL-CAw: a refinement of MCL for detecting yeast complexes from weighted PPI networks by incorporating core-attachment structure
Source: BMC Bioinformatics. 2010 Oct 12;11:504. doi: 10.1186/1471-2105-11-504 (PMC2965181; doi:10.1186/1471-2105-11-504)
Supplement: Additional files 2 — The MCL-CAw software package: The source code and installation details for the MCL-CAw software. [file 1471-2105-11-504-S2.ZIP › Additional_files_2/mcl-09-149.tar.gz!/mcl-09-149/doc/mcxarray.html]

The mcxarray manual


29 May 2009   
**mcxarray**
1.008, 09-149

1.

NAME

2.

SYNOPSIS

3.

DESCRIPTION

4.

OPTIONS

5.

AUTHOR

6.

SEE ALSO

## NAME

mcxarray — Transform array data to MCL matrices

## SYNOPSIS

**mcxarray** [options]

**mcxarray**
**[-data** fname (*input data file*)**]**
**[-skipr** <num> (*skip <num> data rows*)**]**
**[-skipc** <num> (*skip <num> data columns*)**]**
**[-write-tab** <fname> (*write row labels to file*)**]**
**[-l** <num> (*take labels from column <num>*)**]**
**[-imx** fname (*input matrix file*)**]**
**[-n** mode (*normalize input*)**]**
**[-o** fname (*output file fname*)**]**
**[-digits** <num> (*output precision*)**]**
**[-co** num (*cutoff for output values*)**]**
**[-cutoff** num (*cutoff for output values*)**]**
**[-gq** num (*ignore input smaller than*)**]**
**[-lq** num (*ignore input larger than*)**]**
**[--transpose** (*work with the transpose*)**]**
**[--cosine** (*use cosine*)**]**
**[--pearson** (*use Pearson correlation (default)*)**]**
**[-tear** num (*inflate input columns*)**]**
**[-teartp** num (*inflate tranposed columns*)**]**
**[-pi** num (*inflate the result*)**]**

**[--apropos** (*print this help*)**]**
**[-h** (*print this help*)**]**
**[--version** (*print version information*)**]**


## DESCRIPTION

**mcxarray** can either read a flat file containing array data (**-data**)
or a matrix file satisfying the mcl input format (**-imx**). In the
former case it will by default work with the rows as the data vectors. In
the latter case it will by default work with the columns as the data
vectors (note that mcl matrices are presented as a listing of columns).
This can be changed for both using the
**--transpose** option.

## OPTIONS

**-data** fname (*input data file*)

Specify the data file containing the expression values.
It should be tab-separated.

**-imx** fname (*input matrix file*)

The expression values are read from a file in mcl matrix format.

**-skipr** <num> (*skip <num> data rows*)

Skip the first *<num>* data rows.

**-skipc** <num> (*skip <num> data columns*)

Ignore the first *<num>* data columns.

**-l** <num> (*take labels from column <num>*)

Specifies to construct a tab of labels from this data column.
The tab can be written to file using **-write-tab** *fname*.

**-write-tab** <fname> (*write row labels to file*)

Write a tab file. In the simple case where the labels are in the first
data column it is sufficient to issue **-skipc** **1**.
If more data columns need to be skipped one must explicitly specify
the data column to take labels from with **-l** *l*.

**-n** mode (*normalization mode*)

If *mode* is set to **z** the data will be normalized
based on z-score. No other modes are currently supported.

**-o** fname (*output file fname*)

Output file name.

**-digits** <num> (*output precision*)

Specify the precision to use in native interchange format.

**-co** num (*cutoff for output values*)

**-cutoff** num (*alias for **-co***)

Output values smaller than *num* are removed (set to zero).

**-gq** num (*ignore input smaller than*)

Input values smaller than *num* are ignored.

**-lq** num (*ignore input larger than*)

Input values larger than *num* are ignored.

**--transpose** (*work with the transpose*)

Work with the transpose of the input data matrix.

**--cosine** (*use cosine*)

Use the cosine as correlation measure.

**--pearson** (*use Pearson correlation (default)*)

Compute the Pearson correleation coefficient as the similarity between the
data columns.

**-tear** num (*inflate input columns*)

Apply (column-wise) inflation with parameter *num* to the input
values before further processing.

**-teartp** num (*inflate tranposed columns*)

Apply (column-wise) inflation with parameter *num* to the input
values in the transposed matrix before further processing.

**-pi** num (*inflate the result*)

Apply inflation with parameter *num* to the output values.

**--apropos** (*print help*)

**-h** (*print help*)

**--version** (*print version information*)

## AUTHOR

Stijn van Dongen.

## SEE ALSO

mcl,
mclfaq,
and mclfamily for an overview of all the documentation
and the utilities in the mcl family.
