# Supplementary material for: MCL-CAw: a refinement of MCL for detecting yeast complexes from weighted PPI networks by incorporating core-attachment structure
Source: BMC Bioinformatics. 2010 Oct 12;11:504. doi: 10.1186/1471-2105-11-504 (PMC2965181; doi:10.1186/1471-2105-11-504)
Supplement: Additional files 2 — The MCL-CAw software package: The source code and installation details for the MCL-CAw software. [file 1471-2105-11-504-S2.ZIP › Additional_files_2/mcl-09-149.tar.gz!/mcl-09-149/doc/mcxdump.html]

The mcxdump manual


29 May 2009   
**mcxdump**
1.008, 09-149

1.

NAME

2.

SYNOPSIS

3.

DESCRIPTION

4.

OPTIONS

5.

AUTHOR

6.

SEE ALSO

## NAME

mcxdump — dump matrices, optionally map indices to labels

## SYNOPSIS

**mcxdump**
**[-imx** <fname> (*matrix file*)**]**
**[-imx-cat** <fname> (*concatenation matrix file*)**]**
**[-imx-tree** <fname> (*concatenation cone file*)**]**
**[--skeleton** (*read empty matrix, honour domains*)**]**
**[-o** <fname> (*output file name ('-' for stdout)*)**]**
**[-digits** <num> (*output precision*)**]**
**[-tab** <fname> (*row/column tab (label) file*)**]**
**[-tabc** <fname> (*column tab file*)**]**
**[-tabr** <fname> (*row tab file*)**]**
**[--lazy-tab** (*allow tab/domain mismatch*)**]**
**[--transpose** (*work with the transpose*)**]**
**[--no-values** (*omit values*)**]**
**[--no-loops** (*omit loops*)**]**
**[--force-loops** (*force loops*)**]**
**[--dump-pairs** (*emit pairs per line*)**]**
**[--dump-lines** (*emit rows per line*)**]**
**[--dump-rlines** (*omit leading identifier*)**]**
**[--dump-lead-off** (*omit leading identifier*)**]**
**[--dump-lower** (*dump lower part excluding diagonal*)**]**
**[--dump-loweri** (*dump lower part including diagonal*)**]**
**[--dump-upper** (*dump upper part excluding diagonal*)**]**
**[--dump-upperi** (*dump upper part including diagonal*)**]**
**[--write-tabc** (*dump tab file on column domain*)**]**
**[--write-tabr** (*dump tab file on row domain*)**]**
**[--dump-domc** (*dump column domain*)**]**
**[--dump-domr** (*dump row domain*)**]**
**[--dump-table** (*dump table format*)**]**
**[--dump-lead-off** (*do not dump leading identifiers*)**]**
**[-table-nfields** <num> (*output first <num> fields*)**]**
**[-table-nlines** <num> (*output first <num> lines*)**]**
**[--table-keys** (*dump field/cell identifiers*)**]**
**[--newick** (*output newick format*)**]**
**[-newick** [NBI]+ (*exclude Number|Branch-length|Indent*)**]**
**[--write-matrix** (*(deconcatenate) write matrices*)**]**
**[-split-stem** <str> (*(deconcatenate) matrices file name stem*)**]**
**[-cat-max** <num> (*(deconcatenate) write first <num> matrices*)**]**
**[-dump** <fname> (*alias for -o*)**]**
**[-sep-value** <str> (*node/value separator*)**]**
**[-sep-field** <str> (*field separator*)**]**
**[-sep-lead** <str> (*lead separator*)**]**
**[-sep-cat** <str> (*concatenation separator*)**]**
**[-sort** size-{ascending,descending} (*vector sort mode*)**]**
**[-h** (*print synopsis, exit*)**]**
**[--apropos** (*print synopsis, exit*)**]**
**[--version** (*print version, exit*)**]**

## DESCRIPTION

**mcxdump** reads a data file satisfying the mcl input format
(refer to mcxio). It outputs a line-based format. The
**--dump-pairs** option yields a single matrix entry per line,
identified by the respective column and row identifiers (either index or
label) separated by the field separator.
The **--dump-lines** and **--dump-rlines** result in the
joining of all row entries on a single line, separated by the field
separator. For both formats, the matrix value corresponding with
a particular entry is by default output as well.

**mcxdump** can also act on files that contain concatenated
matrices. Refer to the group of options headed by
**-imx-cat** *fname*.

## OPTIONS

**-imx** <fname> (*matrix file*)

Input matrix.

**--transpose** (*work with the transpose*)

Work with the tranpsose of the input matrix.

**--skeleton** (*read empty matrix, honour domains*)

No entries are read, only domains.

**-o** <fname> (*output file name*)

Output stream. Use - for STDOUT.

**-digits** <num> (*output precision*)

Specify the precision to use in native interchange format.

**-tab** <fname> (*row/column tab (label) file*)

Substitute column indices and row indices by labels from the tab file.
Since the same tab file is used for both, this implies that the matrix
domains are identical.

**-tabc** <fname> (*column tab file*)

Substitute column indices by labels from the tab file.

**-tabr** <fname> (*row tab file*)

Substitute row indices by labels from the tab file.

**--lazy-tab** (*allow tab/domain mismatch*)

If used, the tab file domain(s) do not necessarily need to match
the corresponding domain in the input matrix. Entries missing in
the tab files will be replaced by a question mark.

**--no-values** (*omit values*)

Do not emit values.

**--no-loops** (*omit loops*)

Do not output entries for which the row index equals the column index,
if present.
Applies only to matrices for which column and row domains are equal.

**--force-loops** (*force loops*)

For each column, force output of a row entry that matches the
column index.
Applies only to matrices for which column and row domains are equal.

**--dump-pairs** (*emit pairs per line*)

**--dump-lines** (*emit rows per line*)

**--dump-rlines** (*omit leading column node*)

**--dump-lead-off** (*do not dump leading identifiers*)

**--dump-lower** (*dump lower part excluding diagonal*)

**--dump-loweri** (*dump lower part including diagonal*)

**--dump-upper** (*dump upper part excluding diagonal*)

**--dump-upperi** (*dump upper part including diagonal*)

**--dump-pairs** is the default mode of output. Each matrix entry
is output as a single pair of column-identifier and row-identifier per line,
optionally followed by the value of the corresponding matrix entry.
All fields are separated by the field separator.

With **--dump-lines**, each matrix column is output on a
single line, with row identifiers separated by the field separator
and values attached to the row identifier by the node/value separator.
In this format, the column identifier is output as the leading field.

**--dump-rlines** is as **--dump-lines**,
except that the column identifier is not output.
Use **--dump-lead-off** to preclude the output of the leading
identifiers (for line-based outputs).

The options pertaining to *lower* and *upper* dumps currently
only work with **--dump-pairs**. They act to only output
the specified part of the matrix.

**--dump-table** (*dump table format*)

**-table-nfields** (*field limit*)

**-table-nlines** (*line/row limit*)

**--table-keys** (*do dump field/cell identifiers*)

Output table format. In table format no indices are printed by default
and all values
are printed including zeroes. The options **-table-nfields** and **-table-nlines**
can be used to limit
the number of fields and lines to be printed. Note that fields correspond
to MCL matrix rows and that lines correspond to MCL matrix columns, as MCL
calls its primary indices column indices.
Use **--table-keys** to include identifiers (tab-derived if a tab file is specified).
Use **--dump-lead-off** to preclude the output of the leading
identifiers (for line-based outputs).

**--newick** (*output newick format*)

**-newick** [NBI]+ (*newick, exclude Number|Branch-length|Indent*)

Boo.

**--write-tabc** (*dump tab file on column domain*)

**--write-tabr** (*dump tab file on row domain*)

**--dump-domc** (*dump column domain*)

**--dump-domr** (*dump row domain*)

These options work in conjunction with the **-ixm** *fname* option.
Only the domains from the input matrix are read as if **--skeleton**
was specified.
**--write-tabc** assumes the input tab file envelopes the matrix column
domain, and it outputs a new tab file restricted to that domain.
**--write-tabr** acts analogously for the row domain.
**--dump-domc** and **--dump-domr** respectively dump the column
and domain as a regular dump, outputting labels in case a tab file is
specified.

These options are implemented as ensembles of other options.
For example, **--dump-domr** **-imx** *fname* corresponds with
**--dump-lines** **--transpose** **--skeleton**.

**--write-tabr** (*dump tab file on row domain*)

This will only infer the domains from the input matrix. It assumes
the input tab file envelopes the matrix row domain, and it outputs
a new tab file restricted to that domain.

**-imx-cat** <fname> (*concatenation matrix file*)

**-imx-tree** <fname> (*concatenation cone file*)

**--write-matrix** (*(deconcatenate) write matrices*)

**-split-stem** <str> (*(deconcatenate) matrices file name stem*)

**-cat-max** <num> (*(deconcatenate) write first <num> matrices*)

**-imx-cat** is like **-imx** except that the input is assumed to
contain multiple concatenated matrices.
The matrices are dumped separated by the
*cat separator* (cf. **-sep-cat**).
Alternatively, the matrices can be written to different files
using the **-split-stem** option.
In this case it is possible to output each matrix in native format
rather than as a dump by specifying **--write-matrix**.
This makes mcxdump effectively act as a deconcatenator.
In all cases (respectively dumping and writing matrices
to either the same stream or multiple files) the number of
matrices to be dumped can be limited with **-cat-max**.

**-imx-tree** is like **-imx-cat** except that the input
is assumed to be in cone format (the format output by **mclcm**).
This format encodes a tree as a concatenation of matrices with
nested domains. **mcxdump** will project all levels of this tree
so that all row domains are the same as the bottom row domain.
This implies that a set of nested clusterings (on different node
sets, as the set of clusters of a given level is the node set
of the next level) is transformed
into a set of flattened clusterings, all on the same node set.
If you do not want this to happen, simply use **-imx-cat**.

**-sep-value** <str> (*node/value separator*)

Set the node/value separator for line based row ensemble output.

**-sep-field** <str> (*field separator*)

Set the field separator for different row indices in a given column.

**-sep-lead** <str> (*lead separator*)

Set the lead separator. In the **--dump-lines** format it
separates the leading column index from the following ensembl of
row indices. It can be useful to make this different from the
field separator. One can for example grep for columns that have
more than one entry in a matrix mapping nodes to clusters. This
will find nodes in overlap.

**-sep-cat** <str> (*concatenation separator*)

Set the separator that is used between matrix dumps when a concatenation of
matrices is dumped.

**-sort** size-{ascending,descending} (*concatenation separator*)

Reorder the matrix columns prior to dumping, based on the number of
nonzero entries in each column.
Do not use this in conjunction with a tab file for the column domain.

## AUTHOR

Stijn van Dongen.

## SEE ALSO

mcxload,
mcl,
mclfaq,
and mclfamily for an overview of all the documentation
and the utilities in the mcl family.
