# Supplementary material for: MCL-CAw: a refinement of MCL for detecting yeast complexes from weighted PPI networks by incorporating core-attachment structure
Source: BMC Bioinformatics. 2010 Oct 12;11:504. doi: 10.1186/1471-2105-11-504 (PMC2965181; doi:10.1186/1471-2105-11-504)
Supplement: Additional files 2 — The MCL-CAw software package: The source code and installation details for the MCL-CAw software. [file 1471-2105-11-504-S2.ZIP › Additional_files_2/mcl-09-149.tar.gz!/mcl-09-149/doc/mcxdiameter.html]

The mcx diameter manual


29 May 2009   
**mcx diameter**
1.008, 09-149

1.

NAME

2.

SYNOPSIS

3.

DESCRIPTION

4.

OPTIONS

5.

SEE ALSO

## NAME

mcx diameter — compute the diameter of a graph

## SYNOPSIS

**mcx diameter** [options]

mcxdiameter is not in actual fact a program. This manual
page documents the behaviour and options of the mcx program when
invoked in mode *diameter*. The options **-h**, **--apropos**,
**--version**, **-set**, **--nop**, **-progress** *<num>*
are accessible
in all **mcx** modes. They are described
in the mcx manual page.

**mcx diameter**
**[-abc** <fname> (*specify label input*)**]**
**[-imx** <fname> (*specify matrix input*)**]**
**[-tab** <fname> (*use tab file*)**]**
**[--list** (*list eccentricity for all nodes*)**]**
**[-h** (*print synopsis, exit*)**]**
**[--apropos** (*print synopsis, exit*)**]**
**[--version** (*print version, exit*)**]**

## DESCRIPTION

**mcx diameter** computes the diameter of a graph. The input graph should be
symmetric. Results will be unpredictable for directed graphs.
For label input this is irrelevant as **mcx diameter** will create
a symmetric graph from the input.

The input graph/matrix, if specified with the **-imx** option, has to
be in mcl matrix/graph format. You can use label input instead by using the
**-abc** option.
Refer to mcxio for a description of these two input formats.
By default **mcx diameter** reads from STDIN *and expects matrix format*.
To specify label input from STDIN use **-abc** **-**.

## OPTIONS

**-abc** <fname> (*label input*)

The file name for input that is in label format.

**-imx** <fname> (*input matrix*)

The file name for input that is in mcl native matrix format.

**-tab** <fname> (*use tab file*)

This option causes the output to be printed with the labels
found in the tab file.
With **-abc** this option will, additionally, construct
a graph only on the labels found in the tab file.
If this option is used in conjunction with **-imx** the
tab domain and the matrix domain are required to be identical.

**--list** (*list eccentricity for all nodes*)

Print eccentricity for all nodes. The eccentricity of
a node is the distance to any node that is the furthest
away from it. The diameter of a graph is the maximum
of the eccentricity taken over all nodes in a graph.
In this mode **mcx diameter** will not output the diameter
itself.

## SEE ALSO

mcxio,
and mclfamily for an overview of all the documentation
and the utilities in the mcl family.
