# Supplementary material for: MCL-CAw: a refinement of MCL for detecting yeast complexes from weighted PPI networks by incorporating core-attachment structure
Source: BMC Bioinformatics. 2010 Oct 12;11:504. doi: 10.1186/1471-2105-11-504 (PMC2965181; doi:10.1186/1471-2105-11-504)
Supplement: Additional files 2 — The MCL-CAw software package: The source code and installation details for the MCL-CAw software. [file 1471-2105-11-504-S2.ZIP › Additional_files_2/mcl-09-149.tar.gz!/mcl-09-149/doc/mcxctty.html]

The mcx ctty manual


29 May 2009   
**mcx ctty**
1.008, 09-149

1.

NAME

2.

SYNOPSIS

3.

DESCRIPTION

4.

OPTIONS

5.

REFERENCES

6.

SEE ALSO

## NAME

mcx ctty — compute the clustering coefficient of a graph

## SYNOPSIS

**mcx ctty** [options] [matrix-file]

mcxctty is not in actual fact a program. This manual
page documents the behaviour and options of the mcx program when
invoked in mode *ctty*. The options **-h**, **--apropos**,
**--version**, **-set**, **--nop**, **-progress** *<num>*
are accessible
in all **mcx** modes. They are described
in the mcx manual page.

**mcx ctty**
**[-abc** <fname> (*specify label input*)**]**
**[-imx** <fname> (*specify matrix input*)**]**
**[-tab** <fname> (*use tab file*)**]**
**[--list** (*list clustering coefficient for all nodes*)**]**
**[-h** (*print synopsis, exit*)**]**
**[--apropos** (*print synopsis, exit*)**]**
**[--version** (*print version, exit*)**]**

## DESCRIPTION

**mcx ctty** computes centrality betweenness for all nodes in a graph, using
the between centrality update algorithm from [1].

The input graph/matrix, if specified with the **-imx** option, has to
be in mcl matrix/graph format. You can use label input instead by using the
**-abc** option.
Refer to mcxio for a description of these two input formats.
By default **mcx diameter** reads from STDIN *and expects matrix format*.
To specify label input from STDIN use **-abc** **-**.

## OPTIONS

**-abc** <fname> (*label input*)

The file name for input that is in label format.

**-imx** <fname> (*input matrix*)

The file name for input. STDIN is assumed if not specified.

**-tab** <fname> (*use tab file*)

This option causes the output to be printed with the labels
found in the tab file.
With **-abc** this option will, additionally, construct
a graph only on the labels found in the tab file.
If this option is used in conjunction with **-imx** the
tab domain and the matrix domain are required to be identical.

**--list** (*print result for each node*)

Print clustering coefficient for all nodes.

## REFERENCES

[1]
Ulrik Brandes, *A Faster Algorithm for Betweenness Centrality*.
Journal of Mathematical Sociology 25(2): 163-177, (2001).
http://citeseerx.ist.psu.edu/viewdoc/summary?doi=10.1.1.11.2024

## SEE ALSO

mcxio,
and mclfamily for an overview of all the documentation
and the utilities in the mcl family.
