# Supplementary material for: MCL-CAw: a refinement of MCL for detecting yeast complexes from weighted PPI networks by incorporating core-attachment structure
Source: BMC Bioinformatics. 2010 Oct 12;11:504. doi: 10.1186/1471-2105-11-504 (PMC2965181; doi:10.1186/1471-2105-11-504)
Supplement: Additional files 2 — The MCL-CAw software package: The source code and installation details for the MCL-CAw software. [file 1471-2105-11-504-S2.ZIP › Additional_files_2/mcl-09-149.tar.gz!/mcl-09-149/doc/mcxrand.html]

The mcxrand manual


29 May 2009   
**mcxrand**
1.008, 09-149

1.

NAME

2.

SYNOPSIS

3.

DESCRIPTION

4.

OPTIONS

5.

SEE ALSO

## NAME

mcxrand — random shuffling, removal, addition, and perturbation of edges of graphs

## SYNOPSIS

**mcxrand** [options]

```
mcxrand -gen K -add N -new-g-mean f    # random graph on K nodes, N edges
mcxrand -imx <name> -remove N -add N   # remove then add edges
mcxrand -imx <name> -shuffle N         # shuffle N edge pairs
mcxrand -imx <name> -noise-radius f    # add noise to add weights
```

**mcxrand**
**[-imx** <fname> (*input matrix*)**]**
**[-o** <fname> (*output matrix to <fname>*)**]**
**[--write-binary** (*write output in binary format*)**]**
**[-gen** <num> (*generate new graph*)**]**
**[-remove** <num> (*remove <num> edges*)**]**
**[-add** <num> (*add <num> edges not yet present*)**]**
**[-shuffle** <num> (*shuffle edge pair <num> times*)**]**
**[-noise-radius** <num> (*maximum spread to add noise with*)**]**
**[-noise-sdev** <num> (*noise standard deviation*)**]**
**[-noise-range** <num> (*noise range factor*)**]**
**[-edge-min** <num> (*absolute edge weight minimum*)**]**
**[-edge-max** <num> (*absolute edge weight maximum*)**]**
**[-new-g-mean** <num> (*mean for new edges (Gaussian)*)**]**
**[-new-g-sdev** <num> (*standard deviation for new edges (Gaussian)*)**]**
**[-new-g-radius** <num> (*maximum spread for new edges (Gaussian)*)**]**
**[-new-g-min** <num> (*lower bound selection (Gaussian)*)**]**
**[-new-g-max** <num> (*upper bound selection (Gaussian)*)**]**
**[-skew** <num> (*skew towards min or max*)**]**
**[-h** (*print synopsis, exit*)**]**
**[--apropos** (*print synopsis, exit*)**]**
**[--version** (*print version, exit*)**]**

## DESCRIPTION

This utility is a recent addition to the mcl suite and the schemes
explained below will likely be evolved, simplified, and extended
with future releases.

The input graph/matrix, if specified with the **-imx** option,
has to be in mcl matrix/graph format.
You can use label input instead by preprocessing the label input
with mcxload, i.e.

```
   mcxload -abc <label-file> | mcxrand [options]
```

Refer to mcxio for a description of these two input formats.
By default **mcxrand** reads from STDIN. Change this with the **-imx**
option.

**mcxrand** can randomly remove and add edges to a graph, or add gaussian noise
to the edge weights of a graph. It can also shuffle edge pairs while
preserving the degree sequence of the graph.
In *removal mode* (**-remove** *<num>*) and in
*addition mode* (**-add** *<num>*)
**mcxrand** enforces arc symmetry by only working with edges
*w(i,j)* where *i < j* and symmetrifying the result and adding any
loops that were present in the input graph just before the output stage.

In *perturbation mode* (**-noise-radius**, with no other mode specified)
the input can be any graph.

*Shuffle mode* (**-shuffle** *<num>*) requires an undirected graph
but will only fail when it picks an arc for which the arc in the reverse
direction is not present. This means it may or may not fail on directed
input depending on the random choices. It does not check equality of the two
arc weights and randomly picks one to represent the edge weight.

Edge removal, edge creation, and edge perturbation are applied in this order
if both are specified. Edge shuffling presently cannot be combined with one
of the previous modes.

A random graph can be generated with **-gen** *k*, which
specifies the number of nodes the graph should have. It is equivalent
with pasing (the file name of) an empty graph of the same dimensions as the argument
to **-imx**.

When adding (i.e. creating) edges, the default is to use the uniform
distribution for new edge weights ranging in some interval.
The default interval is [0,1] and can be modified using the
**-edge-min** *min* and **-edge-max** *max* options.
A Gaussian edge weight distribution can be obtained by specifying
**-new-g-mean** *num*. The standard deviation is by default
1.0 and can be altered with **-new-g-sdev** *num*.
Currently the edge weigths are generated within the interval
[*mean-radius*, *mean+radius*] where *radius* is specified
with **-new-g-radius**. They are further selected to
lie within the interval *[L,R]* if and only if
**-new-g-min** *L* and **-new-g-max** *R* have
been specified.

For both uniform and Gaussian edge creation the edge weights
can be skewed towards either side of the distribution with
**-skew** *c*. Skewing is applied by mapping
the edge weights to the interval [0,1], applying
the function *x^c*, and remapping the resulting number.
For values *c<1* this skews the edge weights towards
the right bound and for values *c>1* this skews the edge
weights towards the left bound.
This is a rather crude approach that will likely be
changed in the future.

Edge weights can be perturbed by specifying **-noise-radius** *radius*.
This sets the maximum perturbation allowed. Noise is generated with
a standard deviation that is by default set to 0.5 and can be altered
using **-noise-sdev** *num*.
Values are generated in the interval *[-fac\*sdev, fac\*sdev]*
where *fac* is set with **-noise-range** *fac*.
This interval is mapped to the interval *[-radius, radius]* before
the resulting value is added to the actual edge weight.
This becomes the new value. If an interval *[L,R]* is explicitly
specified using **-edge-min** *L* and **-edge-max** *R*
then the new value will be accepted only if it lies within the interval,
otherwise the edge will not be perturbed.

## OPTIONS

**-imx** <fname> (*input matrix*)

The file name for input. STDIN is assumed if neither
**-imx** nor **-gen** *num* is specified.

**-o** <fname> (*output matrix to <fname>*)

The file to write the transformed matrix to.

**--write-binary** (*write output in binary format*)

Write the output matrix in native binary format.

**-shuffle** <num> (*shuffle edge pair <num> times*)

Shuffle edge pair <num> times. An edge shuffle
acts on two randomly chosen edges
edges *w(a,b)* and *w(c,d)* where all the nodes must
be different. If either none of the edges in *w(a,c)*, *w(b,d)*
or none of the edges in *w(a,d)*, *w(b,c)* exists
the original two edges are removed and is replaced
by an edge pair for which both edges did not exist before.

**-remove** <num> (*remove <num> edges*)

Remove this many edges from the input graph.

**-add** <num> (*add <num> edges not yet present*)

Create this many new edges.

**-gen** <num> (*node number*)

Use in conjunction with **-add** to generate
a random graph on *<num>* nodes.

**-noise-radius** <num> (*maximum spread to add noise with*)

**-noise-sdev** <num> (*standard deviation*)

**-noise-range** <fac> (*number of standard deviations allowed*)

See the discussion in DESCRIPTION.

**-edge-min** <num> (*global edge weight minimum*)

**-edge-max** <num> (*global edge weight maximum*)

See the discussion in DESCRIPTION.

**-skew** <num> (*skew towards min or max*)

See the discussion in DESCRIPTION.

**-new-g-mean** <num> (*mean to generate new edges with*)

**-new-g-sdev** <num> (*standard deviation to generate new edges with*)

**-new-g-radius** <num> (*maximum spread to generate new edges with*)

**-new-g-min** <num> (*lower bound selection (Gaussian)*)

**-new-g-max** <num> (*upper bound selection (Gaussian)*)

See the discussion in DESCRIPTION.

## SEE ALSO

mcxio,
and mclfamily for an overview of all the documentation
and the utilities in the mcl family.
