# Supplementary material for: MCL-CAw: a refinement of MCL for detecting yeast complexes from weighted PPI networks by incorporating core-attachment structure
Source: BMC Bioinformatics. 2010 Oct 12;11:504. doi: 10.1186/1471-2105-11-504 (PMC2965181; doi:10.1186/1471-2105-11-504)
Supplement: Additional files 2 — The MCL-CAw software package: The source code and installation details for the MCL-CAw software. [file 1471-2105-11-504-S2.ZIP › Additional_files_2/mcl-09-149.tar.gz!/mcl-09-149/doc/mcxload.html]

The mcxload manual


29 May 2009   
**mcxload**
1.008, 09-149

1.

NAME

2.

SYNOPSIS

3.

DESCRIPTION

4.

OPTIONS

5.

AUTHOR

6.

SEE ALSO

## NAME

mcxload — load matrices and tab files from label format

## SYNOPSIS

**mcxload** **-abc** <fname> (*label file*)
**-o** <fname> (*output file*)

**[-abc** <fname> (*label file*)**]**
**[-123** <fname> (*identifier file*)**]**
**[-o** <fname> (*output file*)**]**
**[--stream-mirror** (*symmetrify, same domain*)**]**
**[--graph** (*assume same domain*)**]**
**[-re** <mode> (*edge deduplication mode*)**]**
**[-ri** <mode> (*image symmetrification mode*)**]**
**[-etc** <fname> (*leader 'etc' label file*)**]**
**[-etc-ai** <fname> (*leaderless 'etc' label file*)**]**
**[-235** <fname> (*leader '235' label file*)**]**
**[-235-ai** <fname> (*leaderless '235' label file*)**]**
**[-write-tab** <fname> (*save domain tab*)**]**
**[-write-tabc** <fname> (*save column tab*)**]**
**[-write-tabr** <fname> (*save row tab*)**]**
**[-strict-tab** <fname> (*tab universe*)**]**
**[-strict-tabc** <fname> (*tabc universe*)**]**
**[-strict-tabr** <fname> (*tabr universe*)**]**
**[-restrict-tab** <fname> (*tab world*)**]**
**[-restrict-tabc** <fname> (*tabc world*)**]**
**[-restrict-tabr** <fname> (*tabr world*)**]**
**[-extend-tab** <fname> (*tab launch*)**]**
**[-extend-tabc** <fname> (*tabc launch*)**]**
**[-extend-tabr** <fname> (*tabr launch*)**]**
**[--stream-log** (*log transform stream values*)**]**
**[--stream-neg-log** (*minus log transform stream values*)**]**
**[-stream-tf** (*transform stream values*)**]**
**[-tf** <tf-spec> (*transform (not so) final matrix*)**]**
**[--transpose** (*transpose*)**]**
**[--write-binary** (*output binary format*)**]**
**[--debug** (*debug*)**]**
**[-h** (*print synopsis, exit*)**]**
**[--apropos** (*print synopsis, exit*)**]**
**[--version** (*print version, exit*)**]**

## DESCRIPTION

**mcxload** reads label input from a file. The format of the file should be
line-based, each line containing two white-space separated strings (labels)
and optionally a number separated from the second label by whitespace. In
the absence of a value, mcxload will use the default value 1.0. If a tab is
present on an input line, mcxload will assume that the tab character is the
separator for that line. Lines for which the first non-whitespace character
is an octothorpe ('#') are skipped.

**mcxload** will transform the labels into mcl numerical identifiers and the
pairs of labels into graph edges or equivalently matrix entries. The weight
of an edge is the value associated with the associated labels. mcxload
constructs dictionaries (sometimes just one) that map labels onto mcl
identifiers as it goes along. It can optionally write these to file. In MCL
(family) parlance, such a dictionary written to file is called a *tab
file*.

It is possible to specify numerical identifiers directly with
the **-123** option. In this case **mcxload** assumes a canonical
domain (cf mcxio) and will create the minimal canonical
domain that supports the data. Also bear in mind the caveat further
below.

It is possible to effectively predeclare labels and thus enforce
an a-priori known mapping of labels onto numerical identifiers.
Labels receive an identifier in the order in which they occur
in the input. Predeclaring labels can be achieved by
having them appear in the desired order and setting the edge
weight to zero.

A major mcxload modality is whether the input refers to a single
domain or to two separate domains. An example of the first is where
labels are names of people and the value is the extent to which they
like one another. This encodes a *likability* graph where all
the nodes represent people. The reasonable thing to do in this
case is to create a single dictionary with all names wherever
they occur. All **tab** options (as opposed to **tabc** and **tabr**)
pertain to this scenario and likewise for the options **--graph**
and **--stream-mirror**.

An example of the second mode is where the first label is again the name of
a person, the second label is the name of an animal species, and the value
is the extent to which that person appreciates the species. In this case,
the reasonable thing to do is to create two dictionaries, one for persons
and one for species. All **tabc** and **tabr** options pertain to
this scenario. The **tabc** options *always refer to the first label*
and the **tabr** options *always refer to the second label*.
The letters **c** and **r** refer to *column* and *row* respectively.
The latter are the names of the matrix domains corresponding
to the input domains. Refer to mcxio.

A further mcxload modality is whether it constructs dictionaries
on the fly, or whether it proceeds from a tab file already
available.
By default mcxload will construct dictionaries on the fly. You
need to save them with the appropriate **-write** option(s).
All the **strict** options read a tab file
and require any labels in the **-abc** *label input*
to be present in the corresponding tab file. mcxload will then fail in
the face of absent labels.
All the **restrict** options simply ignore labels that are
not found in the corresponding tab file.
The **extend** options extend the existing tab file with
labels that are not found.
It presumably only makes sense to do so if the corresponding
**-write** options are used as well.

The input stream is deduplicated on a per-node neighbourhood basis
using the **-re** option.

mcxload has a few options to transform or select based on
the values in the input stream and the values in the
constructed matrix. These are
**--stream-log**,
**--stream-neg-log**,
**-stream-tf** and
**-tf**.
Refer to mcxio for a description of the syntax accepted
by the latter two options — it is a syntax accepted
by a few more mcl siblings.
Finally it is possible to transpose the final result
using the **--transpose** option. Keep in mind that
mcxload does not accordingly change its idea of row and
column domains.

The final matrix can be symmetrified using the **-ri** option.

The **-etc** and **-235** options may be useful for
the odd job. These options assume a format where all entries for a given
column (or equivalently all neighbours for a given node) are joined onto a
single line. This can be useful e.g. to read in externally generated
clusterings. The **-etc** options expects label input, whereas the
**-235** options expects numbers in the input that are mapped
directly onto mcl numerical identifiers.

**CAVEAT**  
Please note that by feeding the line '1000000000 1' to **mcxload** with either
of the **-235** or **-123** options it will try to allocate a
matrix with one billion columns. This is most likely not what is wanted.
Assuming that the input contains fewer than one billion unique labels, one
should use the label options as described above and below.

**STAGES**  
Conceptually, input matrix creation consists of the following stages

i

Read the input stream, apply **-stream-tf** transformation
specification, and optionally push reverse elements
(**--stream-mirror**).

ii

Deduplicate edges in the context of all edges/arcs originating from
a given node according to the **-re** option.

iii

Apply transpose symmetrification according to the
**-ri** option, if used.

iv

Apply **-tf** transformation specification.

## OPTIONS

**-abc** <fname> (*label file*)

The file to read label data from. Labels are separated by white-space. The
labels may optionally be followed by a value (again separated by
white-space), which is taken as the edge weight between the nodes
corresponding with the labels. If a tab is present on an input line it is
presumed to be the separator for that line, including the value if present.
Lines for which the first non-blank character is the octothorpe ('#')
are skipped.

**-123** <fname> (*identifier file*)

The file to read numerical data from. The format is the same as
for label data, but the identifiers are directly mapped onto mcl identifiers
as described earlier.

**-o** <fname> (*output file*)

The output file where the constructed matrix is written.

**--stream-mirror** (*symmetrify, same domain*)

Whenever *label1* *label2* *value*
is encountered in the input, mcxload inserts
*label2* *label1* *value* in the input
stream as well. This option implies that both labels
belong to the same domain.

**--graph** (*assume same domain*)

This tells mcxload that both labels belong to the same domain.

**-re** <max|add|mul|first|last> (*deduplication mode*)

This specifies how mcxload should collapse repeated entries, that is edges
for which a value is specified multiple times. This is done relative to a
single node at a time, taking into account all neighbours assembled from the
input stream. Note that **--stream-mirror** will result in
duplicated entries if the input contains edge specifications in both ways.
Also note that **first** and **last** might not result in
symmetric input if only **--stream-mirror** is used.

**-write-tab** <fname> (*save domain tab*)

Write the domain to file. It applies to both label types.

**-write-tabc** <fname> (*save column tab*)

Write the column domain to file. It applies to the first label found
on each input line.

**-write-tabr** <fname> (*save row tab*)

Write the column domain to file. It applies to the second label found
on each input line.

**-strict-tab** <fname> (*tab universe*)

Read a dictionary from file and require each label to be present in the
dictionary. mcxload will exit on absentees.

**-strict-tabc** <fname> (*tabc universe*)

Read a dictionary from file and require the first label on each line
to be present in the dictionary. mcxload will exit on absentees.

**-strict-tabr** <fname> (*tabr universe*)

Read a dictionary from file and require the second label on each line
to be present in the dictionary. mcxload will exit on absentees.

**-restrict-tab** <fname> (*tab world*)

Read a dictionary from file and only accept input lines (edges)
for which both labels are present in the dictionary.
mcxload will ignore absentees.

**-restrict-tabc** <fname> (*tabc world*)

Read a dictionary from file and ignore input lines
for which the first label is absent from the dictionary.

**-restrict-tabr** <fname> (*tabr world*)

Read a dictionary from file and ignore input lines
for which the second label is absent from the dictionary.

**-extend-tab** <fname> (*tab launch*)

Read a dictionary from file and extend it with any
label from the input not yet present in the dictionary.

**-extend-tabc** <fname> (*tabc launch*)

Read a dictionary from file and extend it with all
first labels from the input not yet present in the dictionary.

**-extend-tabr** <fname> (*tabr launch*)

Read a dictionary from file and extend it with all
second labels from the input not yet present in the dictionary.

**--stream-log** (*log transform stream values*)

Replace each entry by its natural logarithm.

**--stream-neg-log** (*minus log transform stream values*)

Replace each entry by the negative of its natural logarithm.
This is most likely useful to convert scores that denote probabilities
or p-values such as BLAST scores.

**-stream-tf** (*transform stream values*)

Transform the stream values as they are read in according
to the syntax described in mcxio.

**-tf** <tf-spec> (*transform (not so) final matrix*)

Transform the matrix values after deduplication and symmetrification
according to the syntax described in mcxio.

**-ri** (*<max|add|mul>*)

After the initial matrix has been assembled, it can be symmetrified by
either of these options. They indicate the operation used to combine the
entries of the transposed matrix and the original matrix. **mul**
is special in that it treats missing entries (which are normally considered
zero in mcl matrix operations) as one.

**--transpose** (*transpose*)

Write the transposed matrix to file. This is obviously not useful
when a symmetric matrix has been generated.

**-etc** <fname> (*leader 'etc' label file*)

**-etc-ai** <fname> (*leaderless 'etc' label file*)

**-235** <fname> (*leader '235' label file*)

**-235-ai** <fname> (*leaderless '235' label file*)

The input is read in lines; each line is split on whitespace into labels.
For **-etc** the first label is interpreted as the source node. All
other labels are interpreted as destination nodes. Currently no values are
recognized, but this functionality may be introduced in the future.
For **-etc-ai** (*auto-increment*) all labels are interpreted as
destination nodes and mcxload automatically creates a source node for each
line it reads. This option can be useful to read in files encoding a
clustering, where each line represents a cluster of white-space separated
labels.

The **-235** options are similar except that the input is not
interpreted as labels but must consist of numbers that explicitly
specify the matrix to be built.

**--write-binary** (*output binary format*)

The output matrix is written in native binary format — refer to
mcxio.

**--debug** (*debug*)

Among other things, this turns on warnings when **restrict** tab
files are used and labels are found to be missing.

## AUTHOR

Stijn van Dongen.

## SEE ALSO

mcxio,
mcxdump,
mcl,
mclfaq,
and mclfamily for an overview of all the documentation
and the utilities in the mcl family.
