# Supplementary material for: MCL-CAw: a refinement of MCL for detecting yeast complexes from weighted PPI networks by incorporating core-attachment structure
Source: BMC Bioinformatics. 2010 Oct 12;11:504. doi: 10.1186/1471-2105-11-504 (PMC2965181; doi:10.1186/1471-2105-11-504)
Supplement: Additional files 2 — The MCL-CAw software package: The source code and installation details for the MCL-CAw software. [file 1471-2105-11-504-S2.ZIP › Additional_files_2/mcl-09-149.tar.gz!/mcl-09-149/doc/mcxsubs.html]

The mcxsubs manual


29 May 2009   
**mcxsubs**
1.008, 09-149

1.

NAME

2.

SYNOPSIS

3.

DESCRIPTION

4.

OPTIONS

5.

SUBMATRIX SPECIFICATIONS

6.

AUTHOR

7.

SEE ALSO

## NAME

mcxsubs — extract submatrices (subgraphs) of a matrix (graph).

**mcxsubs** can be used to
inspect local cluster structure in a graph, for example by looking at the
subgraph induced by a single cluster or a couple of clusterings, or the
edge set where all tail nodes are from a set of domains and all head
nodes are from the complement of this set, and many other variants and
refinements.

Additionally, mcxsubs enables

•

selecting matrix entries based on value.

•

making the result characteristic (set all nonzero values to 1.0).

•

pruning empty columns and empty rows.

•

transposing the result.

•

remapping the indices of the result to consecutive indices.

•

and other miscellaneous operations.

## SYNOPSIS

**mcxsubs** **-imx** <fname> [options] <specs>+

**mcxsubs**
**-imx** <fname> (*input matrix*)
**[-dom** <fname> (*domain matrix*)**]**
**[-tf** spec (*apply tf-spec to input matrix*)**]**
**[--block** (*use block matrix*)**]**
**[--blockc** (*use complement of block matrix*)**]**
**[--skin-read** (*read domain structure without entries*)**]**
**[--extend** (*read extended submatrices*)**]**
**[-tab** (*read tab file*)**]**
**[--from-disk** (*space/speed optimizer*)**]**
**[-out** <fname> (*special purpose output file name*)**]**
**[-efac** <num> (*random edge selection*)**]**
**[-dfac** <num> (*random domain selection*)**]**
**[-rfac** <num> (*random column selection*)**]**
**[-cfac** <num> (*random row selection*)**]**
**[--rand-discard** (*remove random selections*)**]**
**[--rand-merge** (*merge random selections*)**]**
**[--rand-intersect** (*intersect random selections*)**]**
**[--rand-exclusive** (*only random selections*)**]**
**[-tag-digits** k (*set precision*)**]**
**[--tag** (*tag nodes*)**]**
<specs>+

If you are a frequent **mcxsubs** user with very large graphs, consider
converting the input matrix into binary format using
mcxconvert and then using the mcxsubs
**--from-disk** option. This should give you a 400-fold speed gain.

## DESCRIPTION

**mcxsubs** lets you extract submatrices/subgraphs corresponding with index
sets and (possibly) sets of domains from a given domain matrix (e.g.
a matrix representing a clustering). Columns and
rows of the target submatrix can be specified both independently and
simultaneously, and can be specified as unions of simple index and domain
ranges and complements of these.

Because a submatrix or subgraph specification is composed of type,
location, column, row, index, and set specifications,
the word specification is
abbreviated as spec. Multiple submatrices can be specified
simultaneously. A submatrix is created for each submatrix spec. Everything
encountered after the **mcxsubs** options are exhausted should be a spec.
The syntax of specs is described in the SUBMATRIX SPECIFICATIONS section.
By default, the domains of the submatrix will be set to the domains
as described in the specification. This can be changed using the
*uni* directives from the **fin** part.

## OPTIONS

**-imx** <fname> (*input matrix*)

Submatrices will all be selected from the matrix in file <fname>.
This option is obligatory, and throughout this manual its argument
is called the *source matrix*.

**-dom** <fname> (*domain matrix*)

Submatrices are specified in terms of sets (or domains) of
indices. These sets are specified by (the label of) the vectors
from the matrix given by this options.

**--block** (*use block matrix*)

This replaces the input matrix by the block diagonal matrix
induced by the domain matrix specified by the **-dom** option.
It works by including a block for each domain in the domain matrix,
and will work if there are overlapping domains. That is, it
will not include overlapping parts more than once.
The output file name can either be specified in the submatrix
specification language using the **out**(*fname*) directive,
or with the **-out** option.

**--blockc** (*use complement of block matrix*)

This replaces the input matrix by the complement of the block diagonal
matrix described above, and selection of the output name
is exactly the same.

**--skin-read** (*read domain structure without entries*)

This reads a skeleton matrix by only considering its domain structure
and not reading any entries, pertaining to the matrix
specified by the **-imx** option.

**-tf** spec (*apply tf-spec to input matrix*)

Transform the input matrix values according
to the syntax described in mcxio.

**-tab** (*read tab file*)

Read a tab file. Its domain can be used by the t and T
indicators.

**--extend** (*read extended submatrices*)

This causes the selection of submatrices where either a row index
is in the selected row domain or a columns index is in the selected
column domain or both. Equivalently it only excludes matrix entries
for which neither the column nor row index is in the selected domains.
The resulting matrix has both domains equal to the source matrix.

**-out** <fname> (*special purpose output file name*)

This specifies an output name that can be used for special purposes.
Normally, output file names are specified in the submatrix specification
using the **out**(*fname*) directive.

**-efac** <num> (*random edge selection*)

*num* should be inbetween zero and one. It denotes the probability
with which each edge is selected.

**-dfac** <num> (*random domain selection*)

*num* should be inbetween zero and one. It denotes the probability
with which entries in the domains will make it into the randomized
selection used for subsequent processing. By default the randomized
selection is intersected with whatever the other selection criteria (if any)
yield, i.e. the behaviour under the **--rand-intersect** option.

If the column and row domain are identical, they are submitted to
the same selection process and will end up identical. If you don't want
this, use **-rfac** and **-cfac** separately.

If you just want a randomized selection, doing

```
mcxsubs -imx foo -dfac 0.5 'out(foo.rnd)'
```

will not yield the expected result. Randomized selections only work
if a domain has explicitly been specified. The minimal way to achieve
this is the following:

```
mcxsubs -imx foo -dfac 0.5 'dom(cr), out(foo.rnd)'
```

**-rfac** <num> (*random column selection*)

As **-dfac**, limited to the column domain.

**-cfac** <num> (*random row selection*)

As **-dfac**, limited to the row domain.

**--rand-merge** (*merge random selections*)

The random selection(s) of domains identify parts of the
matrix that will be merged with the result of the main
selection process (default).

**--rand-discard** (*remove random selections*)

The random selection(s) of domains identify parts of the
matrix that will be removed from the result of the main
selection process.

**--rand-intersect** (*intersect random selections*)

The random selection(s) of domains identify parts of the
matrix that will be intersected with the result of the main
selection process. This is the default.

**--rand-exclusive** (*only random selections*)

The random selection(s) of domains identify parts of the
matrix from which the result of the main
selection process will be removed.

**-tag-digits** k (*set precision*)

Sets the precision for tagged output.
Setting it to **-tag-digits** **-1**
disables the output of values altogether — only the node indices
and the cluster indices are written.

**--tag** (*tag nodes*)

Each node in the column (tail node) listing of the matrix (graph)
spec is tagged with the domain it is in. This requires
the use of the **-dom** option.
This output mode, called *tagged matrix*, is currently not
recognized by any of the **mcl**/**mcx** input routines. It is present
to facilitate easier visual inspection of clustering results.

**--from-disk** (*space/speed optimizer*)

Use this if the input graph is in binary format, or if the
input graph is very large and the subgraph(s) to extract
are small in comparison, or if the available memory
does not comfortably exceed the size of the graph.

The effect of this option is that the subgraph will be read
directly from disk, without reading in the entire graph
in advance. This will be done repeatedly for all subgraphs that are
specified.

This option reduces memory consumption to the size of the subgraph(s)
to be extracted.

For graphs in interchange format, the speed gain is not
dramatic. If more than one subgraph is specified, there will most
likely be a loss in speed.

With input graphs in binary format, **mcxsubs** will
be \*very\* much faster, to the extent of 400-fold speed gains.
It does not matter whether more than one subgraph is specified.

## SUBMATRIX SPECIFICATIONS

A submatrix or subgraph spec may contain a number of spec parts. Each part
is specified in a function-style notation. Different parts are separated by
commas. Parts may occur multiple times, but for most parts only the last one
specified will be effective. The spec parts are the following: **dom**,
**ext**, **val**, **size**, **fin**, and **out**. These are described
below in the sections DOMAINS, EXTENSION, VALUES, SIZE, FINALIZE and OUTPUT.

**DOMAINS**  
The domain part is specified as

**dom**(*X* <, *Y*(*ispec*) >+)

Here *X* is the row/column indicator. Rows are indicated with
either r or R, columns are indicated with either
c or C. *X* may contain one or two indicators, with
a single indicator per column domain and row domain allowed.
Uppercase indicators indicate that the complement is being specified
relative to the corresponding domain in the target matrix.

*Y* is the type indicator, it is exactly one of
i, I, d, D, c, r, t, or T.
The i/I indicators specify
that *ispec* contains a simple index specifation.
The d/D specify that *ispec* contains domain indices.
*ispec* must contain a comma-separated list of integers or
integer ranges (e.g. 2, 5, 4-8).
c and r are restricted indicators that refer
to the domains in the *domain matrix*.
t and T are restricted indicators that refer
to the domain encoded in the tab file as specified by the
**-tab** option. Their usage is described further below.

For domain specifications (d/D) the columns indexed by
these integers in the matrix specified in the **-dom**
option will be fetched and merged. If **-dom** was not used
the target matrix itself (as specified by the **-imx** option) will be
used. For simple specifications
(i/I) the result is simply the list of integers itself. Uppercase
indicators indicate that the complement is being specified.

Examples:

dom(cr, i(0-6,10,11-14))

Principal submatrix on indices 0-6, 10, and 11-14 — all column and row
indices are from this set. Equivalently, this encodes the subgraph on
nodes 0-6, 10, 11-14. The 'c' stands for column, the 'r' for row, and the
'i' for index. It is also possible to specify a 'd' part
(standing for domain), this is shown further below.

dom(c, i(0-6,10,11-14)), dom(r, i(1-6,10,11-14))

Equivalent (but less clear) spec of the above.

dom(cR, i(0-6,10,11-14))

Matrix with column indices in 0-6, 10, 11-14, and row indices
in the complement of this set. Corresponds with all edges going
*out* from the set 0-6, 10, 11-14. Complements are triggered
by the use of a capital; see the next examples.

dom(c, i(0-6,10,11-14)), dom(R, i(1-6,10,11-14))

dom(c, i(0-6,10,11-14)), dom(r, I(1-6,10,11-14))

Both these examples are equivalent to the previous one.
In the last example, the capital 'I' indicates that the complement
should be taken. In this example, 'r' combined with 'I' has the
same effect as 'R' combined with 'i'.

dom(c, d(3,5-9)), dom(r, d(8-14), i(10-30))

Column indices are taken from the domains 3, 5-9 (from the
domain matrix specified by **-dom**), row indices are taken
from domains 8-14 plus the indices 10-30.

dom(cR, d(0-2))

Column indices are all indices from domains 0-2, row indices
are all other indices. This gives all edges going *out*
from domains 0-2.

The use of 'D' is analogous to that of 'C', 'R', and 'I'.
Thus, D(0-3,8,21-30) specifies all indices which are in the
complement of the set formed by taking the union of
domains 0-3,8,21-30.

The c and r indicators must be followed by a
pair of matching parentheses. They specify to take respectively
the column domain and the row domain of the domain matrix (cf. **-dom**).

The t and T indicators must be followed by a
pair of matching parentheses. They specify to take the domain
found in the tab file or its complement.

As seen above, indices (either representing themselves or domains) are
entered as comma-separated lists of single indices, ranges of indices
(which may overlap), or staircases of indices (lists of indices with
a fixed increment inbetween successive indices).
The union of the corresponding elements is taken and
passed along. Before anything else, the result set is replaced by its
complement if 'I' is specified (for simple indices) or 'D' is specified
(for domains). If there is both an index and a set spec string, the
union of the results of both is taken and passed along. If the latter
result is passed to either 'C' or 'R', it is replaced by its complement.

A range is specified e.g. as 10-14 and it is inclusive, denoting
in this case the indices {10,11,12,13,14}.

**EXTENSION**  
The extension part is specified as

**ext**( < **disc**(*k*) | **cdisc**(*k*) | **rdisc**(*k*) >)

This option requires the input matrix to be held in memory. This implies it
will not work with the **--from-disk** option.

This *assumes that the input matrix encodes a graph*, so the column
and row domains must be equal.
It will take the currently selected domain (column domain for
**disc** and **cdisc**, row domain for **rdisc**), and add
all nodes to it that are reachable in *k* steps.
The **disc** variant replaces both column and row domains by
the extended domain, the other variants just change a single domain.

Setting *k* to -1 results in adding *all* nodes that
can be reached from the start domain.

**VALUES**  
The value part is specified as

**val**(<tf-spec>)

It transforms or removes values according to *<tf-spec>*.
Refer to mcxio for a description of the transformation
specification syntax and the available transformation primitives.

**SIZE**  
The size part is specified as

**size**( < **lt**(*x*) | **ceil**(*x*) | **gq**(*x*) | **rmgq**(*x*) > + )

Where *x* is a nonnegative integer, and multiple specifications
are separated by commas.
The strings 'lt', 'lq', 'gq', 'gt', respectively denote
*less than*,
*less than or equal to*,
*greater than or equal to*,
and
*greater than*.

This prunes or removes column vectors based on their size. If **lt**
is used, column vectors are removed if the number of entries exceeds
the specified bound. If **gq** is used, vectors are discarded
if the number of entries is smaller than the specified bound. If a column
vector has an excess of entries over the bound specified by **ceil**, the
smallest entries are removed. Ties are not arbitrarily broken, implying that
the resulting vector may still have more entries than the specified bound.

**FINALIZE**  
The finalize part is specified as
**fin**( < *key* > )
where *key* is a string and multiple keys are separated by commas. The
corresponding actions are generally applied to the matrix that was extracted
according to the domain and value specifications. Exceptions are indicated
below. Currently, there is a fixed order in which actions are considered,
corresponding with the order in which they are listed below.

skel

This creates an empty submatrix on the specified domains, and does
*not* fill it with the corresponding entries from the
source matrix.
Options from the **fin** part that affect the column and
row domains of a matrix will still be in effect.

uni

unir

unic

After the submatrix is selected from the source matrix, its domains
are changed to mirror one or both of the domains of the source matrix.

tp

The resulting submatrix is replaced by its transpose.

cc

The resulting submatrix is made characteristic.

scrubc

scrubr

scrubg

scrub

Domains are shrunk if there are no corresponding entries in the matrix.
This is done for both domains if scrub is specified, for the
column domain if scrubc is specified, and for the row domain
if scrubr is specified. Thus, with scrubc columns are removed
from the domain and the matrix if they are empty.
With scrubg the union of the resulting domains is taken.

mapc

mapr

map

The appropriate domains are mapped onto consecutive indices starting at zero.

**OUTPUT**  
The output part is specified as
**out**( *fname* <, *key* >\* )
that is, the *fname* option is obligatory if the **out** part is specified.
Currently, key can be a single directive, namely **wb** specifying that
the resulting matrix should be output in binary format.

## AUTHOR

Stijn van Dongen.

## SEE ALSO

mcx,
and mclfamily for an overview of all the documentation
and the utilities in the mcl family.
