# Supplementary material for: MCL-CAw: a refinement of MCL for detecting yeast complexes from weighted PPI networks by incorporating core-attachment structure
Source: BMC Bioinformatics. 2010 Oct 12;11:504. doi: 10.1186/1471-2105-11-504 (PMC2965181; doi:10.1186/1471-2105-11-504)
Supplement: Additional files 2 — The MCL-CAw software package: The source code and installation details for the MCL-CAw software. [file 1471-2105-11-504-S2.ZIP › Additional_files_2/mcl-09-149.tar.gz!/mcl-09-149/doc/mcxmap.html]

The mcxmap manual


29 May 2009   
**mcxmap**
1.008, 09-149

1.

NAME

2.

SYNOPSIS

3.

DESCRIPTION

4.

OPTIONS

5.

EXAMPLES

6.

AUTHOR

7.

SEE ALSO

## NAME

mcxmap — permute or remap the indices of graphs and matrices.

## SYNOPSIS

**mcxmap**
**-imx** fname (*input*)

**[-o** fname (*output*)**]**
**[-cann** (*output map file name*)**]**
**[-cannc** (*output map file name*)**]**
**[-cannr** (*output map file name*)**]**
**[-cmul** a (*coefficient*)**]**
**[-cshift** b (*translate*)**]**
**[-rmul** c (*coefficient*)**]**
**[-rshift** d (*translate*)**]**
**[-mul** e (*coefficient*)**]**
**[-shift** f (*translate*)**]**
**[-map** fname (*row/col map file*)**]**
**[-rmap** fname (*row map file*)**]**
**[-cmap** fname (*column map file*)**]**
**[-mapi** fname (*row/col map file (use inverse)*)**]**
**[-rmapi** fname (*row map file (use inverse)*)**]**
**[-cmapi** fname (*column map file (use inverse)*)**]**
**[-tab** fname (*read (and map) tab file*)**]**


## DESCRIPTION

This utility relabels graphs or matrices. Its main use is in applying a
*map file* to a given matrix or graph. A map file contains a so called
*map matrix* in mcl format that has some special properties (given
further below). The functionality of mcxmap can also be provided by **mcx**, as
a mapped matrix (i.e. the result of applying a map matrix to another matrix)
is simply the usual matrix product of a matrix and a map matrix. However,
**mcx** will construct a new matrix and leave the original matrix to be mapped
alone. When dealing with huge matrices, considerable gains in efficiency
memory-wise and time-wise can be achieved by doing the mapping in-place.
This is what mcxmap does. In the future, its functionality may be
embedded in **mcx** with new mcx operators.

The special properties of a map matrix are

•

The column domain and row domain are
of the same cardinality.

•

Each column has exactly one entry.

•

Each row domain index occurs in exactly one column.

These properties imply that the matrix can be used
as a map from the column domain onto the row domain.
An example map matrix is found in the EXAMPLES Section.

## OPTIONS

**-o** fname (*output file*)

Output file.

**-imx** fname (*input file*)

Input file.

**-map** fname (*row/col map file)*)

**-rmap** fname (*row map file*)

**-cmap** fname (*column map file*)

**-mapi** fname (*row/col map file (use inverse)*)

**-rmapi** fname (*row map fil (use inverse)*)

**-cmapi** fname (*column map fil (use inverse)*)

Different ways to specify map files.

**-cann** (*output map file name*)

**-cannc** (*output map file name*)

**-cannr** (*output map file name*)

Generate a map that maps the specified domain onto
the appropriate canonical domain and write the map
matrix to file.

**-cmul** a (*coefficient*)

**-cshift** b (*translate*)

These options have affect if neither a column map file nor column
canonification is specified. If any of the first two options is used,
column indices i are mapped to a\*i+b.

**-rmul** c (*coefficient*)

**-rshift** d (*translate*)

These options have affect if neither a row map file nor row
canonification is specified. If any of the first two options is used,
indices i are mapped to c\*i+d.

**-mul** e (*coefficient*)

**-shift** f (*translate*)

If a map file is specified for a given domain, neither a map file nor
canonification is specified. If any of the first two options is used, the
indices i will be mapped to e\*i+f.

**-tab** fname (*read (and map) tab file*)

This option requires the **-map** option. **mcxmap** will output the
mapped tab definition.

## EXAMPLES

The matrix below has two canonical domains which are identical.
It denotes a map of the canonical domain onto itself, in which
node 0 is relabeled to 8, node 1 is relabeled to 5, et cetera.

```
(mclheader
mcltype matrix
dimensions 12x12
)
(mclmatrix
begin
0  8  $
1  5  $
2  3  $
3  2  $
4  4  $
5  6  $
6  7  $
7  9  $
8  1  $
9  10 $
10 11 $
11 0  $
)
```

## AUTHOR

Stijn van Dongen.

## SEE ALSO

mcxio,
mcx,
mcxsubs,
and mclfamily for an overview of all the documentation
and the utilities in the mcl family.
