# Supplementary material for: MCL-CAw: a refinement of MCL for detecting yeast complexes from weighted PPI networks by incorporating core-attachment structure
Source: BMC Bioinformatics. 2010 Oct 12;11:504. doi: 10.1186/1471-2105-11-504 (PMC2965181; doi:10.1186/1471-2105-11-504)
Supplement: Additional files 2 — The MCL-CAw software package: The source code and installation details for the MCL-CAw software. [file 1471-2105-11-504-S2.ZIP › Additional_files_2/mcl-09-149.tar.gz!/mcl-09-149/doc/mcxq.html]

The mcx q manual


29 May 2009   
**mcx q**
1.008, 09-149

1.

NAME

2.

SYNOPSIS

3.

DESCRIPTION

4.

OPTIONS

5.

SEE ALSO

## NAME

mcxq — compute simple graph statistics

## SYNOPSIS

mcxq is not in actual fact a program. This manual
page documents the behaviour and options of the mcx program when
invoked in mode *q*. The options **-h**, **--apropos**,
**--version**, **-set**, **--nop**, **-progress** *<num>*
are accessible
in all **mcx** modes. They are described
in the mcx manual page.

**mcxq**
**[-imx** <fname> (*specify matrix input*)**]**
**[-o** <fname> (*output file name*)**]**
**[--edge** (*list simple edge statistics*)**]**
**[--edge-list** (*list edge statistics per node*)**]**
**[--dim** (*only list domain sizes*)**]**
**[-h** (*print synopsis, exit*)**]**
**[--apropos** (*print synopsis, exit*)**]**
**[--version** (*print version, exit*)**]**

## DESCRIPTION

**mcxq** computes a number of simple statistics derived from the input
graph. Among these are the number of arcs, the node neighbour count
average, standard deviation, and median, and the edge weight
average, standard deviation, and median.

When instructed to, it can also compute other quantities related
to the input graph. See below.

## OPTIONS

**-imx** <fname> (*input matrix*)

The file name for input that is in mcl native matrix format.

**-o** <fname> (*output file name*)

Set the name of the file where output should be written to.

**--edge** (*compute simple statistics*)

This is the default setting. If any other option (e.g. **--dim**)
is used, **--edge** has to be specified if this output mode is to
be activated in parallel.

**--edge-list** (*compute simple statistics, node-wisel*)

This outputs for each node its neighbour count and the average
weight of the edges connecting that node with those neighbours.

**--dim** (*only list domain sizes*)

This outputs the format (native interchange or native binary)
and the dimensions of the encoded matrix.

## SEE ALSO

mcxio,
and mclfamily for an overview of all the documentation
and the utilities in the mcl family.
