# Supplementary material for: MCL-CAw: a refinement of MCL for detecting yeast complexes from weighted PPI networks by incorporating core-attachment structure
Source: BMC Bioinformatics. 2010 Oct 12;11:504. doi: 10.1186/1471-2105-11-504 (PMC2965181; doi:10.1186/1471-2105-11-504)
Supplement: Additional files 2 — The MCL-CAw software package: The source code and installation details for the MCL-CAw software. [file 1471-2105-11-504-S2.ZIP › Additional_files_2/mcl-09-149.tar.gz!/mcl-09-149/doc/mcxassemble.html]

The mcxassemble manual


29 May 2009   
**mcxassemble**
1.008, 09-149

1.

NAME

2.

SYNOPSIS

3.

DESCRIPTION

4.

OPTIONS

5.

AUTHOR

6.

SEE ALSO

## NAME

mcxassemble — transform raw cooccurrence data to mcl matrix format.

## SYNOPSIS

**mcxassemble**
**-b** base (*base name*)
**[-o** fname (*write to file fname*)**]**
**[--write-binary** (*write output in binary format*)**]**
**[--map** (*apply base.map*)**]**
**[-raw-tf** (*apply transform spec to input*)**]**
**[-rv** MODE (*repeated vectors*)**]**
**[-re** MODE (*repeated entries*)**]**
**[-ri** MODE (*adding mirror image*)**]**
**[-r** MODE (*repeated entries/vectors/images*)**]**
**[-prm-tf** (*apply transform spec to primary matrix*)**]**
**[-sym-tf** (*apply transform spec to symmetrified matrix*)**]**
**[-q** (*quiet mode*)**]**

The options above embody the default setup when using mcxassemble.
There are many more options which mostly provide subtly different
ways of doing input/output, set warning levels, or regulate
how repeated entries and vectors should be treated.
The full list of options is shown below.
Read DESCRIPTION for learning about mcxassemble input/output
and the functionality it provides.

**NOTE**  
As of release 05-314 mcl is able to cluster label-type input
on the fly. In most cases, this will be sufficient. Alternatively,
mcxload can be used to map label-type input onto mcl
matrices. Consequently, there are likely fewer scenarios nowadays
where **mcxassemble** is the best solution. Consider first whether
**mcl** in label mode or **mcxload** can do the job as well.

**mcxassemble**
**[-b** base (*base name*)**]**
**[-hdr** fname (*read header file*)**]**
**[-raw** fname (*read raw file*)**]**
**[--map** (*apply base.map*)**]**
**[--cmap** (*apply base.cmap*)**]**
**[--rmap** (*apply base.rmap*)**]**
**[-map** fname (*apply fname*)**]**
**[-rmap** fname (*apply fname*)**]**
**[-cmap** fname (*apply fname*)**]**
**[-tag** tag (*apply base.tag*)**]**
**[-rtag** tag (*apply base.tag*)**]**
**[-ctag** tag (*apply base.tag*)**]**
**[-skw** fname (*write skew matrix*)**]**
**[-prm** fname (*write primary result matrix*)**]**
**[--skw** (*write base.skw*)**]**
**[--prm** (*write base.prm*)**]**
**[-xo** suf (*write base.suf*)**]**
**[-o** fname (*write to file fname*)**]**
**[-n** (*do not write default symmetrized result*)**]**
**[-i** (*read from single data file*)**]**
**[-digits** int (*digits width*)**]**
**[-s** (*check for symmetry*)**]**
**[-raw-tf** (*apply transform spec to input*)**]**
**[-rv** <mode> (*action for repeated vectors*)**]**
**[-re** <mode> (*action for repeated entries*)**]**
**[-ri** <mode> (*adding mirror image*)**]**
**[-r** <mode> (*same for entries and vectors*)**]**
**[-prm-tf** (*apply transform spec to primary matrix*)**]**
**[-sym-tf** (*apply transform spec to symmetrified matrix*)**]**
**[--quiet-re** (*quiet for repeated entries*)**]**
**[--quiet-rv** (*quiet for repeated vectors*)**]**
**[-q** (*the two above combined*)**]**
**[-h** (*print synopsis, exit*)**]**
**[--apropos** (*print synopsis, exit*)**]**
**[--version** (*print version, exit*)**]**

## DESCRIPTION

**mcxassemble** enables easy matrix creation from an intermediate raw matrix
format that can easily be constructed from a one-pass-parse of cooccurrence
data. The basic setup is as follows.

•

Parse cooccurrence data from some external format.

•

Transform cooccurrence data to raw mcl data as you parse.

•

When done, write out required header and domain information
to a separate file. The domain information can be built during
the parsing stage.

•

Use mcxassemble to construct a valid matrix from the raw data
and the header information.

•

Nodes can optionally be relabeled by writing a separate map file to be read
by **mcxassemble**, which takes the form of a very thin matrix file.

The easiest thing to do is to group all input/output files under the same
base name, say **base**. A standard way of proceeding, which will lead to
a concise **mcxassemble** command line, is by creating the input files
**base.raw** and **base.hdr**, and optionally the file **base.map**. The
default behaviour of mcxassemble is then to create **base.sym** as the
resulting matrix file, containing the symmetrized matrix constructed from
the raw input.

**Example**  
Suppose blastresult is a file containing blast results.
The following two commands construct an mcl matrix file from the file.

```
   mcxdeblast --score=e --sort=a blastresult
   mcxassemble -b blastresult -r max --map
```

**mcxdeblast** will generate the
files blastresult.hdr, blastresult.raw, and blastresult.map.
The **--sort=a** option will create a map file corresponding
with alphabetic ordering. These files are processed by **mcxassemble**
and it will generate the file blastresult.sym. The **-r**
option tells **mcxassemble** that repeated entries should be maxed;
each time the largest entry seen thus far will be taken.

**Header file**  
This file contains a header as usually found in generic mcl matrix files,
i.e. the required *header* part, and optionally the *domain* part(s)
if not all domains are canonical. Refer to mcxio for more information.
The domain information in the header file will be used to pre-construct a
skeleton matrix and to validate the entries in the raw data file as they
fill the skeleton matrix.

**Raw input format**  
The file from which raw input is read should have the raw format as
described in mcxio. Simply put; no header specification, no domain
specification, and no matrix introduction syntax is used. The file just
contains a listing of vectors. An example fragment is the following:

```
2  4:0.34 1:2.8838 4:2.328 1:4.238 1:12 $
1  2:7.8 $
2  1:0.01 4:20.3 3:2 $
```

The listing of vectors need not be sorted, and neither does
a vector itself need to be sorted - the mcl generic matrix format
is actually not different in this respect.
Furthermore, duplicate entries and duplicate vectors are allowed.
This is in fact again allowed in the generic format, except
that where applications expect generic format warnings will be issued and
duplicate entries will be disregarded. **mcxassemble** allows customizable
behaviour dictating how to merge repeated entries.
Refer to the **-re**, **-rv**, **-r**
options below.

The vectors read by **mcxassemble** do have to match the domains specified in
the header file. The leading index that specifies the column index has to be
present in the column domain; all subsequent indices that specify column
entries have to be present in the row domain.

*If one concatenates the contents of the header file and the data file*,
the result is *almost but not quite* a file containing a matrix in
syntactically correct mcl generic matrix format. The parts missing
are the (mclmatrix introduction token, (followed by) the
begin token, and the closing ) token.

**Map file**  
This file must contain a map matrix, which is a matrix with the
following properties:

•

The column domain and row domain are of the same cardinality.

•

Each column has exactly one entry.

•

Each row domain index occurs in exactly one column.

Such a matrix is used to relabel the nodes as found in the raw data. A
situation that might occur when parsing some external format (and producing
raw matrix format), is that ID's (indices) are handed out on the fly during
the parse. Afterwards, one may want to relabel the IDs such that they
correspond with an alphabetic listing of the quantity that is represented by
the node domain, or by some other sort criterion. A map file is then
typically generated by the parser, as that is the utility in charge of the
IDs. A small example of a map file for a graph containing five nodes is the
following:

```
(mclheader
mcltype matrix
dimensions 5x5
)
(mclmatrix
begin
0  4  $  #  mno 
1  2  $  #  ghi
2  1  $  #  def
3  3  $  #  jkl
4  0  $  #  abc
)
```

This corresponds to a relabeling such that the associated strings
will be ordered alphabetically. Note that comments can be used
to link string identifiers with indices. This map file says e.g. that
the string identifier "mno" is represented by index 0 in the raw data,
and by index 4 in the matrix output by **mcxassemble**.

## OPTIONS

**-b** base (*base name*)

Base name of files to be processed and output. Refer to DESCRIPTION
above and the entries of other options below.

**-hdr** fname (*read header file*)

**-raw** fname (*read raw file*)

Explicitly specify the header file and the data file (rather
than constructing the file names from a base name and suffixes).

**--map** (*apply base.map*)

**--cmap** (*apply base.cmap*)

**--rmap** (*apply base.rmap*)

**-map** fname (*apply fname*)

**-rmap** fname (*apply fname*)

**-cmap** fname (*apply fname*)

**-tag** tag (*apply base.tag*)

**-rtag** tag (*apply base.tag*)

**-ctag** tag (*apply base.tag*)

Map options. **--cmap** combines with the **-b** option,
and says that the map file in **base**.cmap (where **base**
was specified with **-b** **base**) should be applied to the column
domain only. **--rmap** works the same for the
row domain, and **--map** can be used to apply the same map
to both the column and row domains.

**-cmap** and its siblings are used to explicitly specify the
map file to be used, rather than combining a base name with a fixed
suffix.
**-tag** and its siblings work in conjuction with
the **-b** option, and require that a tag be specified from
which to construct the map file (by appending it to the base name).

**-skw** fname (*write skew matrix*)

**-prm** fname (*write primary result matrix*)

**--prm** (*write base.prm*)

**--skw** (*write base.skw*)

**-n** (*do not write default symmetrized result*)

Options for writing matrices other than the default symmetrized result.
The primary result matrix is the matrix constructed from reading in the
raw data and adding entries to the skeleton matrix as specified
with the **-r**, **-re**, and **-rv** options.
This matrix can be written using one of the **prm** options.
Calling the primary matrix A, the skew matrix (as defined here)
is the matrix A - A^T, i.e. A minus its transposed matrix.
It can be written using one of the **skw** options.

If for some reason the symmetrized result is not needed, its output
can be prevented using the **-n** option.

**-xo** suf (*write base.suf*)

**-o** fname (*write to file fname*)

**-i** (*read from single data file*)

**-digits** int (*digits width*)

**--write-binary** (*write output in binary format*)

The **-xo** option is used in conjunction with the **-b** option
in order to change the suffix for the file in which the symmetrized
result matrix is written. Use e.g. **-xo** **mci** to change the suffix
from the default value sym to mci. Use **-o** to explicitly
specify the filename in full. Use **-digits** to set the number of
digits written for matrix entries (c.q. edge weights).

The **-i** option is special. It causes
**mcxassemble** to read both the header information and the raw data
from the same file, where the syntax should be fully conforming
to generic mcl matrix format.

**-s** (*check for symmetry*)

This will check whether the primary result matrix was symmetric.
It reports the number of failing (or *skew*) edges.

**-raw-tf** <tf-spec> (*apply transform spec to input*)

**-prm-tf** (*apply transform spec to primary matrix*)

**-sym-tf** (*apply transform spec to symmetrified matrix*)

The first applies its transformation spec to the values
as found in the raw data. The second applies its transformation
spec to the primary matrix. The third applies its transformation
step to the symmetrified matrix.
Refer to mcxio for documentation on the transformation
spec syntax.

**-rv** add|max|min|mul|left|right (*action for repeated vectors*)

**-re** add|max|min|mul|left|right (*action for repeated entries*)

**-ri** add|max|min|mul (*adding mirror image*)

**-r** add|max|min|mul|left|right (*same for entries and vectors*)

Merge options, dictating the behaviour when repeated entries are
found. A distinction is made between entries that are repeated within
the same column listing, and entries that are repeated between
different column listings. An entry can be a repeat of both kinds
simultaneously as well.
Additionally, the final result is by default symmetrized by combining with
the mirror image (in matrix terminology, the *transposed* matrix). This
symmetrization can be done in the same variety of ways.

The **re** option, for repeats within the same column, is carried out
first. It is applied *after* the column has its entries sorted, so the
left and right options are not garantueed to follow the order found
in the raw input. The **rv** option, for repeats over different columns,
is carried out second.

The option **-ri** **min** can assist in implementing
a (top-list) best reciprocal hit criterion.

**Examples**  
The column

```
0 1:30 1:50 2:60 4:70 3:20 1:40 2:40 $
```

is encountered in the input, listing entries for the vector labeled
with index 0. If **-re** **add** or **-r** **add**
is used, it will transform to the vector

```
0 1:120 2:60  3:20 4:70 $
```

If **-re** **max** or **-r** **add**
is used instead, it will transform to the vector

```
0 1:40 2:60 3:20 4:70 $
```

Suppose *add* mode is used, and that later on another
vector specification for the index 0 is found, leading
to this transformed vector:

```
0 1:60 2:80 4:40 $
```

If **-rv** **max** was specified, this new vector is combined with the
previous vector by taking the entry wise maximum:

```
0 1:120 2:60 3:20 4:70 $      # first (transformed) vector
0 1:60 2:80 4:40 $            # second vector

0 1:120 2:80 3:20 4:70 $      # entry wise maximum
```

Finally, suppose that somewhere one or more vector listings
were specified for index 3, which eventually led to an entry 0:50.
The final symmetrization step will take the [0,3]
entry of weight 20 and combine it with the [3,0] entry
of weight 50. The resulting matrix will then have the [0,3]
and the [3,0] entry both equal to either the maximum, the sum,
or the product of the two quantities 50 and 20.

**--quiet-re** (*quiet for repeated entries*)

**--quiet-rv** (*quiet for repeated vectors*)

**-q** (*the two above combined*)

Warning options. Turn these on if you expect the raw data to be free
of repeats.

## AUTHOR

Stijn van Dongen.

## SEE ALSO

mcxio, mcl, mcxload
and mclfamily for an overview of all the documentation
and the utilities in the mcl family.
