# Supplementary material for: MCL-CAw: a refinement of MCL for detecting yeast complexes from weighted PPI networks by incorporating core-attachment structure
Source: BMC Bioinformatics. 2010 Oct 12;11:504. doi: 10.1186/1471-2105-11-504 (PMC2965181; doi:10.1186/1471-2105-11-504)
Supplement: Additional files 2 — The MCL-CAw software package: The source code and installation details for the MCL-CAw software. [file 1471-2105-11-504-S2.ZIP › Additional_files_2/mcl-09-149.tar.gz!/mcl-09-149/doc/mclpipeline.html]

The mclpipeline manual


29 May 2009   
**mclpipeline**
1.008, 09-149

1.

NAME

2.

SYNOPSIS

3.

DESCRIPTION

4.

OPTIONS

5.

AUTHOR

6.

SEE ALSO

## NAME

mclpipeline — a generic pipeline for generating and scrutinizing mcl clusterings.

**NOTE**  
**mcl** has acquired the ability to manipulate label input directly. This
enables a very lightweight mechanism of generating clusterings by teaming up
mcl with a lightweight parser. You might want to use this mechanism.
Example invocations using the **mcxdeblast** BLAST parser are documented in the
mcl manual.

## SYNOPSIS

**mclpipeline** [options] <file-name>  

where <file-name> is either the name of the data input file, or its base
name. In the latter case the **--xi-dat** option is required. In case
mclpipeline is indeed used to control all stages from the data input file
onwards, usage will often be like this:

```
   mclpipeline [prepare options] --prepare-mcl <file-name>
   mclpipeline [cluster options 1] --start-mcl <file-name>
   mclpipeline [cluster options 2] --start-mcl <file-name>
   .. etc
```

**mclpipeline** can also be used to control shorter pipelines, i.e. in
case the input matrix was already created or in case pre-assembled parts
of the input matrix were already created. In this case, usage will
often be like this:

```
   mclpipeline [cluster options 1] --start-mcl=<fname>
   mclpipeline [cluster options 2] --start-mcl=<fname>
or
   mclpipeline [assembly options] --start-assemble=<fname> --prepare-mcl
```

**NOTE**  
It is possible to make mclpipeline output a large arrary
of performance measures related to nodes and clusters
in hyperlinked output by supplying the **--fmt-fancy** option.
This can be useful if one wants to scrutinize a clustering in greater
detail and navigate within the clustering. The output then includes
listings of external nodes that are relevant/close to a given cluster,
and vice versa, listings of external clusters that are relevant/close
to a given node.

Generating this more intricate output requires the presence of the **zoem** macro
processor. Refer to the SEE ALSO section and the
clmformat manual for more information on zoem. By default
zoem is not required, and the return result is a file where each line contains
a clustering consisting of tab-separated labels.

If this program does not work as expected, please file a bug report with the
developer and/or subscribe to mcl-devel as indicated on
http://micans.org/mcl/. The problem will then be fixed.

The full list of pipeline options is given below. Start simple,
and if you need some behaviour, try to see if there is an option
that fits your needs.
If you use a wrapper pipeline such as mclblastline, you
can ignore the **--parser** and **--parser-tag** options
as they are provided by the wrapper.

**mclpipeline**
**--parser=**application (*data parser*)
**--parser-tag=**str (*parse option transporter*)

**[--whatif** (*do not execute*)**]**  
**[--start-assemble** (*skip parse stage*)**]**  
**[--start-mcl** (*skip earlier stages*)**]**  
**[--start-format** (*skip earlier stages*)**]**  
**[--prepare-mcl** (*do preparatory stages*)**]**  
**[--help** (*summary of options*)**]**  
**[--xi=**suf (*strip suf from input file*)**]**  
**[--xo-dat=**suf (*attach suf to parse output*)**]**  
**[--xo-ass=**suf (*attach suf to assembly output*)**]**  
**[--xi-mcl=**suf (*use with --start-mcl*)**]**  
**[--xo-mcl=**suf (*replace mcl output suffix*)**]**  
**[--xa-mcl=**str (*append to mcl output suffix*)**]**  
**[--xe-mcl=**suf (*append to mcl output*)**]**  
**[--xo-fmt=**suf (*attach suf to clmformat output*)**]**  
**[--ass-repeat=**str (*assembly repeat option*)**]**  
**[--ass-nomap** (*ignore map file*)**]**  
**[--ass-opt=**val (*assembly option transporter*)**]**  
**[--mcl-te=**num (*#expansion threads*)**]**  
**[--mcl-I=**float (*mcl inflation value*)**]**  
**[--mcl-i=**float (*mcl initial inflation value*)**]**  
**[--mcl-l=**float (*mcl initial loop length*)**]**  
**[--mcl-c=**float (*mcl center value*)**]**  
**[--mcl-pi=**float (*mcl pre-inflation value*)**]**  
**[--mcl-scheme=**i (*mcl scheme index*)**]**  
**[--mcl-o=**fname (*do not use*)**]**  
**[--mcl-opt=**val (*mcl option transporter*)**]**  
**[--fmt-lump-count=**num (*collect formatted output*)**]**  
**[--fmt-opt** val (*clmformat option transporter*)**]**  
**[--fmt-tab** fname (*use this tab file*)**]**  
**[--fmt-notab** (*ignore tab file*)**]**  
<file-name>

## DESCRIPTION

**mclpipeline** encapsulates a sequence of programs to be run on some
input data in order to obtain clusterings and formatted output
representing the clusterings, while maintaining unique file names
and file name ensembles corresponding with differently parametrized runs.

The script can behave in several ways. By default, the pipeline
consists of the stages of *parsing*, *assembly*,
*clustering*, and *formatting*.
The parsing stage is to be
represented by some parser script obeying the interface
rules described below. The assembly stage is done by
mcxassemble, the clustering stage is done by mcl,
and the formatting stage is done by clmformat.

The script can also be put to simpler uses, e.g. letting the script take
care of unique file names for differently parametrized mcl runs. In this
case there is no need to specify either the parser or the data file, and
subsequent invocations might look like this:

```
   mclpipeline --start-mcl=<fname> --mcl-I=1.6 --mcl-scheme=4
   mclpipeline --start-mcl=<fname> --mcl-I=2.0 --mcl-scheme=4
   mclpipeline --start-mcl=<fname> --mcl-I=2.4 --mcl-scheme=4
   .. etc
```

It is easiest if for each parser a wrapper script is written
encapsulating the parser and **mclpipeline**. A mechanism is provided
through which mclpipeline can recognize options that are meant to be
passed to the parser. An example of such a wrapper script is the BLAST
pipeline **mclblastline** that basically calls mclpipeline with the
parameters **--parser**=**mcxdeblast** **--parser-tag**=**blast**.
In this case the parser is **mcxdeblast**, and mclpipeline will
pass any options of the forms **--blast-foo** and **--blast-bar=zut**
to the parser (respectively as **--foo** and **--bar=zut**).

For a given data set the stages of parsing and assembling
will often not need to be repeated, especially if there
is a well established way of creating a matrix from
the input data. In this case, usage
will look like

```
   mclpipeline [parse/assembly options] --prepare-mcl <file-name>
   mclpipeline [cluster options 1] --start-mcl <file-name>
   mclpipeline [cluster options 2] --start-mcl <file-name>
   mclpipeline [cluster options 3] --start-mcl <file-name>
   ...
```

Note that **mclpipeline** will store the output of those runs
in unique file names derived from the parametrizations.

There are some options that affect the file names of intermediate
results. In the above setup of repeated runs, if used in one run,
they must be used in all runs, as **mclpipeline** uses them to compute the
file names it needs.
For the setup above, these options are
**--xi**=*suf*,
**--xo-dat**=*suf*, and
**--xo-ass**=*suf*.

There are other ways of resuming the pipeline, and one must always take care
that options starting with **--xi-**, **--xo-**, **--xa**, or
**--xe** are repeated among preparatory and subsequent runs.
These tags respectively mnemonize *extension in*, *extension out*,
*extension append*, and *extension extra*.

Should one want to experiment with various ways of creating input
matrices, then **mclpipeline** supplies options to create unique file
names and file name ensembles corresponding with different setups and
parametrizations. These are **--xo-dat**=*suf* for the parsing
stage and **--xo-ass**=*suf* for the assembly stage. mclpipeline
*automatically* generates unique file names for the cluster results,
but it does not do so for the parse and assembly results.

**Parser interface requirements**  
The parser should recognize its last argument as a file name
or as the base name of a file.
It should produce the files base.raw, base.hdr,
and preferably base.tab and base.map, where the base name
base is determined as described below.

**mclpipeline** will pass its last argument <file-name> to the parser.
The parser should recognize the **--xi-dat**=*suf*
and **--xo-dat**=*suf* options. If the first is present,
it should try to strip <file-name> of the suffix specified in
the value and use the result as the initial part of the base name
for the files it constructs. If stripping does not succeed, it
must interpret <file-name> as the base name and append the suffix
in order to construct the name of the file it will try to read.
If the **--xo-dat**=*suf* option is present, it must append the
suffix specified in the value to the base part as described above.
The result is then the full base name to which the raw, hdr,
and other suffixes will be appended.

**Parser interface examples**  

```
<parser> --xi-dat=abc --xo-dat=xyz foo
 *  parser reads foo.abc, writes foo.xyz.raw, foo.xyz.hdr et cetera.
<parser> --xi-dat=abc --xo-dat=xyz foo.abc
 *  idem
<parser> --xo-dat=xyz foo.abc
 *  parser reads foo.abc, writes foo.abc.xyz.raw et cetera.
<parser> --xi-dat=abc foo.abc
 *  parser reads foo.abc, writes foo.raw, foo.hdr et cetera.
<parser> foo.abc
 *  parser reads foo.abc, writes foo.abc.raw, foo.abc.hdr et cetera.
```

**Output file names construction**  
The files of primary interest are the mcl output file and
the formatted output produced by clmformat.
The pipeline constructs a file name for the mcl output
in which several parameters are encoded. The first
part of the file name is either the base name for the assembly
stage, or simply the name of the input file, depending on
whether the option **--xo-ass**=*suf* was used or not.

A suffix encoding key-value pairs is appended. By default
it has the form I..s., e.g. I20s2. The latter examples
denotes primary inflation value 2.0 and scheme 2.
The pipeline will automatically append several other mcl parameters
if they are used. These correspond with the pipeline options
**--mcl-i**=*f*, **--mcl-l**=*i*, **--mcl-c**=*f*,
and **--mcl-pi**=*f*,
which in turn correspond with the mcl options **-i** *f*,
**-l** *i*, **-c** *f*, and **-pi** *f*.
The order of appending is alphabetical with capitals preceding
lowercase, so a full example is I25c30i35l2pi28s3.

## OPTIONS

**--whatif** (*do not execute*)

Shows only what would be done without executing it.
Hugely useful!

**--start-assemble** (*skip parse stage*)

Skip the parse stage, assume the necessary files have been created in a
previous run.

**--prepare-mcl** (*do preparatory stages*)

Do the parsing and assembly stage, then quit. Useful if you
want to do multiple cluster runs for a given graph - use
**--start-mcl**

**--start-mcl** (*skip earlier stages*)

Immediately start the mcl stage.
Assume the necessary files have been created in a previous run.

**NOTE**  
This option can be used as **--start-mcl**=*fname*.
In this case, no final file name argument need be given, and
mcl will use *fname* as the file name for its input.

The difference with **--start-mcl** is that the latter
will assume it is picking up the results of a previous run.
The names of those results might include suffixes corresponding
with the parse and assembly stage (cf. **--xo-dat** and
**--xo-ass**).
If you are not clear on this (and you should not be), exercise
the **--whatif** option to be sure.

**--start-format** (*skip earlier stages*)

Immediately start the format stage.
Assume the necessary files have been created in a previous run.

**--help** (*summary of options*)

Print a terse summary of options.

**--xi** suf (*strip suffix from data file*)

In normal usage, this will strip the specified suffix from the data file
to obtain the base name for further output.
When used with **--start-mcl**=*fname* the same behaviour is applied
to the mcl input file name specified in *fname*.

**--xo-dat** suf (*attach suf to parse output*)

This suffix will be attached to the base name of the parse output.
It can be used to distinguish between different parse parametrizations
if this is applicable.

**--xo-ass** suf (*attach suf to assembly output*)

This suffix will be attached to the base name of the assembly output.
It can be used to distinguish between different assembly parametrizations
if this is applicable.

**--xo-mcl** suf (*replace mcl output suffix*)

This suffix will be used instead of the suffix by default created
by the pipeline.

**--xa-mcl** str (*append to mcl output suffix*)

This string will be appended to the suffix by default created
by the pipeline.

**--xe-mcl** suf (*append to mcl output*)

This string will be appended as a single suffix to the output base
name before mclpipeline appends its own suffix.

**--xo-fmt** suf (*attach suf to clmformat output*)

This suffix will be used instead of the suffix by default used
by the formatting stage.

**--ass-repeat** str (*assembly repeat option*)

Corresponds with the **mcxassemble** **-r** *mode* option.
Refer to the mcxassemble manual.

**--ass-opt** val (*assembly option transporter*)

Transfer **-opt** *val* to **mcxassemble**.

**--ass-nomap** (*ignore map file*)

Either no map file is present or it should be ignored.
For parsers that don't write map files.

**--mcl-I** float (*mcl inflation value*)

The (main) inflation value mcl should use.
*This is the primary mcl option*.

**--mcl-scheme** i (*mcl scheme index*)

The scheme index to use. This options is also important.
Refer to the mcl manual.

**--mcl-te** num (*#expansion threads*)

The number of threads **mcl** should use.

**--mcl-i** float (*mcl initial inflation value*)

The initial inflation value mcl should use.
Only for fine-tuning or testing.

**--mcl-l** float (*mcl initial loop length*)

The length of the loop in which initial inflation
is applied. By default zero.

**--mcl-c** float (*mcl center value*)

The center value. One may attempt to affect granularity
by exercising this option, which controls the loop weights
in the input matrix. Refer to the mcl manual.

**--mcl-pi** float (*mcl pre-inflation value*)

Pre-inflation, another option which may possibly affect granularity by
changing the input matrix. It makes the edge weight
distribution either more or less homogeneous.
Refer to the mcl manual.

**--mcl-o** fname (*do not use*)

Set the mcl output name.

**--mcl-opt** val (*mcl option transporter*)

Transfer **-opt** *val* to **mcl**.

**--fmt-dump-stats** (*add simple measures to dump file*)

This adds some simple performance measures to the dump file. For each
cluster, five columns proceed the label listing. These are the cluster ID,
the number of elements in the cluster, the projection (percentage of
within-cluster edge weight relative to total outgoing edge weight), the
efficiency of the cluster (which is the average of the efficiency of all its
nodes), and the maximum efficiency (average of the max-efficiency of all the
nodes). Look into the clmformat manual for more
information on and references to the efficiency measures.

**--fmt-fancy** (*create detailed output (requires zoem)*)

Creates extensive description of node/cluster and cluster/cluster
relationships.

**--fmt-lump-count** num (*collect formatted output*)

Collect clusters in the same file until the total number
of nodes has exceeded *num* (in the formatted output).
Only meaninful when **--fmt-fancy** is given.

**--fmt-tab** (*use this tab file*)

Explicitly specify the tab file to use.

**--fmt-notab** (*ignore tab file*)

Either no tab file is present or it should be ignored.
For parsers that don't write tab files.

**--fmt-opt** val (*clmformat option transporter*)

Transfer **-opt** *val* to **clm format**.

## AUTHOR

Stijn van Dongen

## SEE ALSO

mcxdeblast, mclblastline,
and mclfamily for an overview of all the documentation
and the utilities in the mcl family.

With default settings, **mclpipeline** depends on the presence
of **zoem**. It can be obtained from
http://micans.org/zoem/ .
