# Supplementary material for: MCL-CAw: a refinement of MCL for detecting yeast complexes from weighted PPI networks by incorporating core-attachment structure
Source: BMC Bioinformatics. 2010 Oct 12;11:504. doi: 10.1186/1471-2105-11-504 (PMC2965181; doi:10.1186/1471-2105-11-504)
Supplement: Additional files 2 — The MCL-CAw software package: The source code and installation details for the MCL-CAw software. [file 1471-2105-11-504-S2.ZIP › Additional_files_2/mcl-09-149.tar.gz!/mcl-09-149/doc/clmimac.html]

The clm imac manual


29 May 2009   
**clm imac**
1.008, 09-149

1.

NAME

2.

SYNOPSIS

3.

DESCRIPTION

4.

OPTIONS

5.

AUTHOR

6.

SEE ALSO

## NAME

clm imac — Interpret Matrices (c.q. MCL iterands output by **mcl**) As Clusterings.

clmimac is not in actual fact a program. This manual
page documents the behaviour and options of the clm program when
invoked in mode *imac*. The options **-h**, **--apropos**,
**--version**, **-set**, **--nop** are accessible
in all **clm** modes. They are described
in the clm manual page.

## SYNOPSIS

**clm imac** -imx <fname> [options]

**clm imac**
**-imx** fname (*input file*)
**[-o** fname (*name/stem for output*)**]**
**[-dag** fname (*output DAG*)**]**
**[-overlap** str (*overlap mode*)**]**
**[-sort** str (*size|revsize|lex|none*)**]**
**[-strict** num (*in 0..1*)**]**
**[-h** (*print synopsis, exit*)**]**
**[--apropos** (*print synopsis, exit*)**]**
**[--version** (*print version, exit*)**]**

## DESCRIPTION

Use **clm imac** to interpret matrices (as clusterings) output by **mcl** using
**mcl**'s **-dump** **ite** option.

Use **clm imac** only if you have a special reason; the normal usage
of **mcl** is to do multiple runs for varying **-I** parameters
and use the clusterings output by **mcl** itself.
One reason is if you are interested in clusterings with overlap;
early MCL iterands generally induce clusterings possessing overlap.
Another reason is to investigate how the cluster structure
associated with the MCL process evolves over time.

## OPTIONS

**-imx** fname (*input file*)

The input file is presumably an MCL iterand resulting
from the mcl option **-dump** **ite**.

**-strict** num (*in 0..1*)

Higher values (up until 1) will thin out the DAG
constructed by **clm imac**. The default value is 0.00001,
yielding the full DAG.

**-o** fname (*file name/stem*)

Write to file named *fname*.

**-dag** fname (*output DAG*)

Write the DAG (directed acyclic graph) constructed from the input to
file. This DAG is constructed according to the structure associated
with diagonally positive semi-definite matrices as described
in the PhD thesis *Graph clustering by flow simulation*.
Consult mclfamily for references.

**-sort** str (*size|revsize|lex|none*)

Sort the clusters either by increasing size, decreasing size,
lexicographically by the indices they contain, or use the clustering
exactly as obtained from the interpretation routine.

**-overlap** mode (*overlap mode*)

With *mode* set to *cut*, remove any overlap by allocating the
nodes in overlap to the first cluster in which they were found. Mode
*keep* leaves overlap unchanged, and mode *split* results in overlapping
parts excised and introduced as clusters in their own right.

## AUTHOR

Stijn van Dongen.

## SEE ALSO

mclfamily for an overview of all the documentation
and the utilities in the mcl family.
