# Supplementary material for: MCL-CAw: a refinement of MCL for detecting yeast complexes from weighted PPI networks by incorporating core-attachment structure
Source: BMC Bioinformatics. 2010 Oct 12;11:504. doi: 10.1186/1471-2105-11-504 (PMC2965181; doi:10.1186/1471-2105-11-504)
Supplement: Additional files 2 — The MCL-CAw software package: The source code and installation details for the MCL-CAw software. [file 1471-2105-11-504-S2.ZIP › Additional_files_2/mcl-09-149.tar.gz!/mcl-09-149/doc/clmclose.html]

The clm close manual


29 May 2009   
**clm close**
1.008, 09-149

1.

NAME

2.

SYNOPSIS

3.

DESCRIPTION

4.

OPTIONS

5.

AUTHOR

6.

SEE ALSO

## NAME

clm close — Fetch connected components from graphs or subgraphs

clmclose is not in actual fact a program. This manual
page documents the behaviour and options of the clm program when
invoked in mode *close*. The options **-h**, **--apropos**,
**--version**, **-set**, **--nop** are accessible
in all **clm** modes. They are described
in the clm manual page.

## SYNOPSIS

**clm close** -imx <fname> [options]

**clm close**
**-imx** fname (*specify matrix input*)
**-abc** fname (*specify label input*)
**-dom** fname (*input domain/cluster file*)
**[-o** fname (*output file*)**]**
**[--write-count** (*output component count*)**]**
**[--write-sizes** (*output component sizes (default)*)**]**
**[--write-cc** (*output components as clustering*)**]**
**[-cc-bound** num (*select components with size at least num*)**]**
**[-tf** spec (*apply tf-spec to input matrix*)**]**
**[-h** (*print synopsis, exit*)**]**
**[--apropos** (*print synopsis, exit*)**]**
**[--version** (*print version, exit*)**]**

## DESCRIPTION

Use **clm close** to fetch the connected components from a graph. Different
output modes are supported (see below). In matrix mode (i.e. using
the **-imx** option) the output returned with
**--write-cc** can be used in conjunction with **mcxsubs**
to retrieve individual subgraphs corresponding to connected components.

## OPTIONS

**-abc** <fname> (*label input*)

The file name for input that is in label format.

**-imx** <fname> (*input matrix*)

The file name for input that is in mcl native matrix format.

**-o** fname (*output file*)

Specify the file where output is sent to. The default is STDOUT.

**-dom** fname (*input domain/cluster file*)

If this option is used, clm close will, as a first step,
for each of the domains in file *fname* retrieve the associated
subgraph from the input graph. These are then further decomposed into
connected components, and the program will process these in the normal
manner.

**--write-count** (*output component count*)

**--write-sizes** (*output component sizes (default)*)

**--write-cc** (*output components as clustering*)

The default behaviour is currently to output the sizes of the
connected components. It is also possible to simply output
the number of components with **--write-count** or to
write the components as a clustering in mcl format with
**-write-cc**.

**-cc-bound** num (*select components with size at least num*)

**-tf** spec (*apply tf-spec to input matrix*)

Transform the input matrix values according
to the syntax described in mcxio.

## AUTHOR

Stijn van Dongen.

## SEE ALSO

mclfamily for an overview of all the documentation
and the utilities in the mcl family.
