# Supplementary material for: MCL-CAw: a refinement of MCL for detecting yeast complexes from weighted PPI networks by incorporating core-attachment structure
Source: BMC Bioinformatics. 2010 Oct 12;11:504. doi: 10.1186/1471-2105-11-504 (PMC2965181; doi:10.1186/1471-2105-11-504)
Supplement: Additional files 2 — The MCL-CAw software package: The source code and installation details for the MCL-CAw software. [file 1471-2105-11-504-S2.ZIP › Additional_files_2/mcl-09-149.tar.gz!/mcl-09-149/doc/clmresidue.html]

The clm residue manual


29 May 2009   
**clm residue**
1.008, 09-149

1.

NAME

2.

SYNOPSIS

3.

DESCRIPTION

4.

OPTIONS

5.

AUTHOR

6.

SEE ALSO

## NAME

clm residue — extend clustering of subgraph to clustering of graph.

clmresidue is not in actual fact a program. This manual
page documents the behaviour and options of the clm program when
invoked in mode *residue*. The options **-h**, **--apropos**,
**--version**, **-set**, **--nop** are accessible
in all **clm** modes. They are described
in the clm manual page.

## SYNOPSIS

**clm residue**
**-icl** fname (*input clustering*)
**-imx** fname (*input graph*)
**[-rpm** fname (*residue projection matrix*)**]**
**[-o** fname (*output clustering file*)**]**

## DESCRIPTION

The cluster file presumably contains a clustering of a subgraph of the
graph G contained by the matrix file, implying that the row domain of
that clustering is a subset of the node domain of the graph. **clm residue** will
compute a simple upwards projection of that clustering, resulting in a
clustering of G.

## OPTIONS

**-imx** fname (*input graph*)

**-icl** fname (*input clustering*)

**-rpm** fname (*residue projection matrix*)

The name for the file in which the residue projection matrix is stored.
It contains for each node and for each cluster in the input clustering
the combined weight of the (edges to the) neighbours of that node
in that cluster.

The default output file name is out.rpm.

**-o** fname (*output clustering file*)

The name for the file in which the majority vote projection clustering
is stored. Aka the majority vote projection clustering storage file name.

This embodies a very crude approach to distributing the missing nodes in the
input clustering onto that same cluster. For each node, the cluster is
taken for which the residue projection is the largest (see above). The
residue (i.e. the set of missing nodes) is initially viewed as a cluster;
the set of nodes for which the projection is largest for the residue itself
will form a new cluster, if it is not empty.

By default output is sent to STDOUT.

## AUTHOR

Stijn van Dongen.

## SEE ALSO

mclfamily for an overview of all the documentation
and the utilities in the mcl family.
