# Supplementary material for: MCL-CAw: a refinement of MCL for detecting yeast complexes from weighted PPI networks by incorporating core-attachment structure
Source: BMC Bioinformatics. 2010 Oct 12;11:504. doi: 10.1186/1471-2105-11-504 (PMC2965181; doi:10.1186/1471-2105-11-504)
Supplement: Additional files 2 — The MCL-CAw software package: The source code and installation details for the MCL-CAw software. [file 1471-2105-11-504-S2.ZIP › Additional_files_2/mcl-09-149.tar.gz!/mcl-09-149/doc/clmvol.html]

The clm vol manual


29 May 2009   
**clm vol**
1.008, 09-149

1.

NAME

2.

SYNOPSIS

3.

DESCRIPTION

4.

AUTHOR

5.

OPTIONS

6.

SEE ALSO

## NAME

clm vol — compute volatile nodes from a set of clusterings

clmvol is not in actual fact a program. This manual
page documents the behaviour and options of the clm program when
invoked in mode *vol*. The options **-h**, **--apropos**,
**--version**, **-set**, **--nop** are accessible
in all **clm** modes. They are described
in the clm manual page.

## SYNOPSIS

**clm vol**
**[-nff-fac** num (*nff factor*)**]**
**[-o** fname (*output file*)**]**
<cl file>+

## DESCRIPTION

**clm vol** computes a score for each node, which at a minimum is equal to one.
The output is in the form of a matrix containing a single column. The score
correlates with what is deemed to be volatile behaviour. For all pairs of
clusterings the set of all intersections is computed, and these are
considered in turn.
An intersection is deemed to be volatile if the size of the intersection is
less than half the size of either parent cluster.
In this case, for each node in the intersection the score is incremented by
one over the size of the intersection.
The size comparison can be made more stringent by increasing
the factor using **-nff-fac** *<num>* option. It is by default set to
0.5, the fraction taken from the parent cluster sizes.
If it is increased all the way up to 1.0, the implication is that an
intersection is always considered volatile unless one of the parent
clusters is a subset of the other parent cluster.

## AUTHOR

Stijn van Dongen.

## OPTIONS

**-nff-fac** num (*nff factor*)

See the discussion at DESCRIPTION.

**-o** fname (*output file*)

## SEE ALSO

mclfamily for an overview of all the documentation
and the utilities in the mcl family.
