# Supplementary material for: MCL-CAw: a refinement of MCL for detecting yeast complexes from weighted PPI networks by incorporating core-attachment structure
Source: BMC Bioinformatics. 2010 Oct 12;11:504. doi: 10.1186/1471-2105-11-504 (PMC2965181; doi:10.1186/1471-2105-11-504)
Supplement: Additional files 2 — The MCL-CAw software package: The source code and installation details for the MCL-CAw software. [file 1471-2105-11-504-S2.ZIP › Additional_files_2/mcl-09-149.tar.gz!/mcl-09-149/doc/clminfo.html]

The clm info manual


29 May 2009   
**clm info**
1.008, 09-149

1.

NAME

2.

SYNOPSIS

3.

DESCRIPTION

4.

OPTIONS

5.

AUTHOR

6.

SEE ALSO

7.

REFERENCES

## NAME

clm info — Compute performance measures for graphs and clusterings.

clminfo is not in actual fact a program. This manual
page documents the behaviour and options of the clm program when
invoked in mode *info*. The options **-h**, **--apropos**,
**--version**, **-set**, **--nop** are accessible
in all **clm** modes. They are described
in the clm manual page.

## SYNOPSIS

**clm info** [options] <graph file> <cluster file> <cluster file>\*

**clm info**
**[-o** fname (*write to file **fname***)**]**
**[-pi** f (*apply inflation beforehand*)**]**
**[-tf** spec (*apply tf-spec to input matrix*)**]**
**[-cl-tree** fname (*expect file with nested clusterings*)**]**
**[-cl-ceil** <num> (*skip clusters of size exceeding <num>*)**]**
**[-cat-max** num (*do at most **num** tree levels*)**]**
**[--node-self-measures** (*dump measure for native cluster*)**]**
**[--node-all-measures** (*dump measure for incident cluster*)**]**
**[-h** (*print synopsis, exit*)**]**
**[--apropos** (*print synopsis, exit*)**]**
**[--version** (*print version, exit*)**]**
<matrix file> <cluster file> <cluster file>\*

## DESCRIPTION

**clm info** computes several numbers indicative for the efficiency with
with a clustering captures the edge mass of a given graph.
Use it in conjunction with **clm dist** to determine which clusterings
you accept. See the EXAMPLES section in **clm dist**
for an example of **clm dist** and **clm info** (and **clm meet**) usage.
Output can be generated for multiple clusterings at the same time.

The **efficiency** factor is described in [1] (see
the REFERENCES section). It tries to balance the dual aims of
capturing a lot of edges or edge weights and keeping the cluster footprint
or area fraction small. The efficiency number has several appealing
mathematical properties, cf. [1]. It is related to, but not derivable from,
the second and third numbers, the *mass fraction* and the
*area fraction*.

The **mass fraction** is defined as follows.
Let **e** be an edge of the graph. The clustering *captures* **e**
if the two nodes associated with **e** are in the same cluster.
Now the mass fraction is the joint weight of all captured edges divided
by the joint weight of all edges in the input graph.

The **area fraction** is roughly the sum of the
squares of all cluster sizes for all clusters in the clustering, divided by
the square of the number of nodes in the graph. It says *roughly*,
because the actual formula uses the quantity **N**\*(**N-1**) wherever it
says square (of **N**) above. A low/high area fraction indicates a
fine-grained/coarse clustering.

## OPTIONS

**-o** fname (*output file name*)

**-pi** f (*apply inflation beforehand*)

Apply inflation to the graph matrix and compute the performance
measures for the result.

**-tf** spec (*apply tf-spec to input matrix*)

Transform the input matrix values according
to the syntax described in mcxio.

**-cl-tree** fname (*expect file with nested clusterings (cone format)*)

**-cl-ceil** <num> (*skip (nested) clusters of size exceeding <num>*)

The specified file should contain a hierarchy of nested
clusterings such as generated by **mclcm**. The output is then
in a special format, undocumented but easy to understand.
Its purpose is to help cherrypick a single clustering
from a tree, in conjunction with the slightly experimental
and undocumented program **mlmfifofum**.

The measure that is used is very slow to compute for large clusters, and
generally it will be outside any interesting range (i.e. it will be small).
Use **-cl-ceil** to skip clusters exceeding the specified size —
**clm info** will directly proceed to subclusters if they exist.

**-cat-max** num (*do at most num levels*)

This only has effect when used with **-cl-tree**.
**clm info** will start at the most fine-grained level, working upwards.

**--node-all-measures** (*dump node-wise criteria for all incident clusters*)

**--node-self-measures** (*dump node-wise criteria for native cluster*)

## AUTHOR

Stijn van Dongen.

## SEE ALSO

mclfamily for an overview of all the documentation
and the utilities in the mcl family.

## REFERENCES

[1] Stijn van Dongen. *Performance criteria for graph clustering and Markov
cluster experiments*. Technical Report INS-R0012, National Research
Institute for Mathematics and Computer Science in the Netherlands,
Amsterdam, May 2000.  
http://www.cwi.nl/ftp/CWIreports/INS/INS-R0012.ps.Z
