# Supplementary material for: MCL-CAw: a refinement of MCL for detecting yeast complexes from weighted PPI networks by incorporating core-attachment structure
Source: BMC Bioinformatics. 2010 Oct 12;11:504. doi: 10.1186/1471-2105-11-504 (PMC2965181; doi:10.1186/1471-2105-11-504)
Supplement: Additional files 2 — The MCL-CAw software package: The source code and installation details for the MCL-CAw software. [file 1471-2105-11-504-S2.ZIP › Additional_files_2/mcl-09-149.tar.gz!/mcl-09-149/doc/clmmate.html]

The clm mate manual


29 May 2009   
**clm mate**
1.008, 09-149

1.

NAME

2.

SYNOPSIS

3.

DESCRIPTION

4.

OPTIONS

5.

AUTHOR

6.

SEE ALSO

## NAME

clm mate — compute best matches between two clusterings

clmmate is not in actual fact a program. This manual
page documents the behaviour and options of the clm program when
invoked in mode *mate*. The options **-h**, **--apropos**,
**--version**, **-set**, **--nop** are accessible
in all **clm** modes. They are described
in the clm manual page.

## SYNOPSIS

**clm mate**
**[-o** fname (*output file name*)**]**
**[-b** (*omit headers*)**]**
**[--one-to-many** (*require multiple hits in <clfile1>*)**]**
**[-h** (*print synopsis, exit*)**]**
**[--apropos** (*print synopsis, exit*)**]**
**[--version** (*print version, exit*)**]**
<clfile1> <clfile2>

## DESCRIPTION

**clm mate** computes for each cluster X in clfile1 all clusters
Y in clfile2 that have non-empty intersection and outputs
a line with the data points listed below.

```
   overlap(X,Y)               # 2 * size(meet(X,Y)) / (size(X)+size(Y))
   index(X)                   # name of cluster
   index(Y)                   # name of cluster
   size(meet(X,Y))
   size(X-Y)                  # size of left difference
   size(Y-X)                  # size of right difference
   size(X)
   size(Y)
   projection(X, clfile2)     # see below
   projection(Y, clfile1)     # see below
```

The projected size of a cluster X relative to a clustering K is
simply the sum of all the nodes shared between any cluster Y in K
and X, duplications allowed. For example, the projected size of
(0,1) relative to {(0,2,4), (1,4,9), (1,3,5)} equals 3.

The overlap between X and Y is exactly
1.0 if the two clusters are identical, and for nearly identical
clusterings the score will be close to 1.0.

All of this information can also be obtained from the
contingency matrix defined for two clusterings.
The [i,j] row-column entry in a contigency matrix between
to clusterings gives the number of entries in the intersection
between cluster i and cluster j from the respective
clusterings. The other information is implicitly present;
the total number of nodes in clusters i and j
for example can be obtained as the sum of entries in row i
and column j respectively, and the difference counts
can then be obtained by substracting the intersection count.
The contingency matrix can easily be computed using **mcx**;
e.g.

```
mcx /clfile2 lm /clfile1 lm tp mul /ting wm
```

will create the contingency matrix in mcl matrix format
in the file ting, where columns range over the clusters
in clfile1.

The output can be put to good use by sorting it numerically on
that first score field. It is advisable to use a stable sort routine
(use the **-s** option for UNIX sort)
From this information one can quickly extract the closest
clusters between two clusterings.

## OPTIONS

**-o** fname (*output file name*)

Specify the name of the output file.

**-b** (*omit headers*)

Batch mode, omit column names.

**--one-to-many** (*require multiple hits in <clfile1>*)

Do not output information for clusters in the first file
that are subset of a cluster in the second file.

## AUTHOR

Stijn van Dongen.

## SEE ALSO

mclfamily for an overview of all the documentation
and the utilities in the mcl family.
