# Supplementary material for: MCL-CAw: a refinement of MCL for detecting yeast complexes from weighted PPI networks by incorporating core-attachment structure
Source: BMC Bioinformatics. 2010 Oct 12;11:504. doi: 10.1186/1471-2105-11-504 (PMC2965181; doi:10.1186/1471-2105-11-504)
Supplement: Additional files 2 — The MCL-CAw software package: The source code and installation details for the MCL-CAw software. [file 1471-2105-11-504-S2.ZIP › Additional_files_2/mcl-09-149.tar.gz!/mcl-09-149/doc/clmmeet.html]

The clm meet manual


29 May 2009   
**clm meet**
1.008, 09-149

1.

NAME

2.

SYNOPSIS

3.

DESCRIPTION

4.

OPTIONS

5.

AUTHOR

6.

SEE ALSO

## NAME

clm meet — compute the intersection of a set of clusterings.

clmmeet is not in actual fact a program. This manual
page documents the behaviour and options of the clm program when
invoked in mode *meet*. The options **-h**, **--apropos**,
**--version**, **-set**, **--nop** are accessible
in all **clm** modes. They are described
in the clm manual page.

## SYNOPSIS

**clm meet**
**[-o** fname (*output file name*)**]**
**[-h** (*print synopsis, exit*)**]**
**[--apropos** (*print synopsis, exit*)**]**
**[--version** (*print version, exit*)**]**
<file name>+

## DESCRIPTION

**clm meet** computes the intersection of a set of clusterings, that is,
the largest clustering that is a subclustering of all the clusterings
in the set. It stores the intersection (or meet) in the argument
to the **-o** flag, which must be the first option given.
All remaining arguments are interpreted as names of files containing
clusterings in mcl format (see mcxio), which must all
pertain to a set of the same cardinality.

This utility can be useful in measuring (in conjunction with
clm dist) the consistency of a set of clusterings at different
levels of granularity — if the meet of all those clusterings is close to
being a subclustering of each of them, consistency is good. See
clm dist for an example.

## OPTIONS

**-o** fname (*output file name*)

## AUTHOR

Stijn van Dongen.

## SEE ALSO

mclfamily for an overview of all the documentation
and the utilities in the mcl family.
