# Supplementary material for: MCL-CAw: a refinement of MCL for detecting yeast complexes from weighted PPI networks by incorporating core-attachment structure
Source: BMC Bioinformatics. 2010 Oct 12;11:504. doi: 10.1186/1471-2105-11-504 (PMC2965181; doi:10.1186/1471-2105-11-504)
Supplement: Additional files 2 — The MCL-CAw software package: The source code and installation details for the MCL-CAw software. [file 1471-2105-11-504-S2.ZIP › Additional_files_2/mcl-09-149.tar.gz!/mcl-09-149/doc/clmorder.html]

The clm order manual


29 May 2009   
**clm order**
1.008, 09-149

1.

NAME

2.

SYNOPSIS

3.

DESCRIPTION

4.

OPTIONS

5.

AUTHOR

6.

SEE ALSO

## NAME

clm order — reorder indices to represent blocks from different clusterings.

clmorder is not in actual fact a program. This manual
page documents the behaviour and options of the clm program when
invoked in mode *order*. The options **-h**, **--apropos**,
**--version**, **-set**, **--nop** are accessible
in all **clm** modes. They are described
in the clm manual page.

## SYNOPSIS

**clm order**
**[-o** fname (*output file*)**]**
<cl-stack-file>

## DESCRIPTION

Given a set of concatenated input clusterings (encoded in a single file/stream),
**clm order** reorders indices so that
the ordering represents blocks from different clusterings (which are
preferably more or less hierarchically organized). It puts nodes that
share many clusters over the different clusters nearby, and puts nodes
in larger clusters earlier in the ordering. It is presumed that the
clusterings are successive subclusterings, but it need not be strictly
the case - **clm order** will convert the input clusterings to a strictly
nested sequence.

**clm order** expects a trailing file name argument for a file
containing concatenated matrices (see mcxio).

## OPTIONS

**-o** fname (*output file*)

## AUTHOR

Stijn van Dongen.

## SEE ALSO

mclfamily for an overview of all the documentation
and the utilities in the mcl family.
