# Supplementary material for: MCL-CAw: a refinement of MCL for detecting yeast complexes from weighted PPI networks by incorporating core-attachment structure
Source: BMC Bioinformatics. 2010 Oct 12;11:504. doi: 10.1186/1471-2105-11-504 (PMC2965181; doi:10.1186/1471-2105-11-504)
Supplement: Additional files 2 — The MCL-CAw software package: The source code and installation details for the MCL-CAw software. [file 1471-2105-11-504-S2.ZIP › Additional_files_2/mcl-09-149.tar.gz!/mcl-09-149/doc/mcxio.html]

The mcl matrix format specification


29 May 2009   
**mcxio**
1.008, 09-149

## NAME

mcxio — the format specifications for input and output in the mcl family.

## DESCRIPTION

This document describes

native matrix format

The format that can be read in by any mcl application
expecting a matrix argument. The native format closely resembles
the layout of matrices as residing in computer memory. There are
two distinct encodings, respectively *interchange* and *binary*.
Their relative merits are described further below.

concatenated native matrix format

This always pertains to matrices in native format concatenated in a single
file, refered to as a *cat file*. It is used for example to encode
hierarchical clusterings as generated by **mclcm**. A cat file either
consists of matrices in interchange format or of matrices in binary format.

raw intermediate format

This is read by mcxassemble.

tab format

Used by applications such as mcl and mcxdump to
convert between meaningful labels describing the input data and the
numerical identifiers used internally.

label format

The format used when streaming labels directly into
mcl or mcxload.

transformation syntax

The syntax accepted by mcl, mcxload, mcxsubs
and mcxassemble to transform values in an input stream
or in input or output matrices.

The interchange format is a portable format that can be transmitted
across computers and over networks and will work with any version
of mcl or its sibling programs. It is documented (here) and very stable.
Applications can easily create matrices in this format.
The drawback of interchange format is that for very large graphs
matrix encodings grow very big and are slow to read.

The binary format is *not* garantueed to be portable across
machines or different versions of mcl or differently compiled
versions of mcl. Its distinct advantage is that for very large
graphs the speed advantage over interchange format is dramatic.

Conversion between the two formats is easily achieved with
mcxconvert. Both mcl and mcxload
can save a matrix in either format after constructing it from
label input.

The concatenated format is generated e.g. by mclcm and can
be transformed by mcxdump using the **-imx-cat**
option. In cat format matrices are simply concatenated,
so it is easily generated from the command line if needed.
For native binary format it is imperative that no additional
bytes are inserted inbetween the matrix encodings. For native
interchange format the only requirement is that the last
matrix is followed by nothing but white space.

A remark on the sloppy naming conventions used for **mcl** and its sibling
utilities may be in order here. The prefix **mcx** is used for generic
matrix functionality, the prefix **clm** is used for generic cluster
functionaliy. The *utility* **mcx** is a general purpose interpreter for
manipulating matrices (and grahps, sets, and clusterings). The set of all
**mcl** siblings (cf. mclfamily) is loosely refered to as the mcl
family, which makes use of the mcl libraries (rather than the mcx
libraries). The full truth is even more horrible, as the mcl/mcx prefix
conventions used in the C source code follow still other rules.

In this document, 'MCL' means 'the mcl setting' or 'the mcl family'. An MCL
program is one of the programs in the mcl family. The remainder of this
document contains the following sections.

3.

Internal representation of matrices in MCL

4.

Specifying matrices

5.

Specifying graphs

6.

Raw format

7.

Tab format / label information

8.

Label input

9.

Transformation syntax

10.

SEE ALSO

11.

AUTHOR

## Internal representation of matrices in MCL

There are several aspects to the way in which MCL represents matrices.
Internally, indices never act as an ofset in an array, and neither do they
participate in ofset computations. This means that they purely act as
identifiers. The upshot is that matrices can be handled in which the index
domains are *non-sequential* (more below). Thus one can work with
different graphs and matrices all using subsets of the same set of
indices/identifiers. This aids in combining data sets in different ways and
easily comparing the respective results when experimenting. Secondly, only
*nonzero values* (and their corresponding indices) are stored. Thirdly,
MCL stores a matrix as a listing of columns. Iterating over a column is
trivial; iterating over a row requires a costly transposition computation.
The last two points should matter little to the user of MCL programs.

In textbook expositions and in many matrix manipulation implementations,
matrices are represented with sequentially indexed rows and columns, with
the indices usually starting at either zero or one. In the MCL setting, the
requirement of sequentiality is dropped, and it follows naturally that no
requirement is posed on the first index. The only requirement MCL poses on
the indices is that they be nonnegative, and can be represented by the
integer type used by MCL. On many machines, the largest allowable integer
will be 2147483647.

MCL associates two domains with a matrix M, the row domain and column
domain. The matrix M can only have entries M[i,j] where i is
in the row domain and j is in the column domain. This is vital when
specifying a matrix: it is illegal to specify an entry M[i,j] violating
this condition. However, it is not necessary to specify *all*
entries M[i,j] for all possible combinations of i and j. One
needs only specify those entries for which the value is nonzero, and only
nonzero values will be stored internally. In the MCL matrix format, the
matrix domains must be specified explicitly if they are not canonical (more
below).

**Strictly as an aside**, the domains primarily exist to ensure data
integrity. When combining matrices with addition or multiplication (e.g.
using the **mcx** utility), MCL will happily combine matrices for which the
domains do not match, although it will usually issue a warning.
Conceptually, matrices auto-expand to the dimensions required for the
operation. Alternatively, a matrix can be viewed as an infinite quadrant,
with the domains delimiting the parts in which nonzero entries may exist.
In the future, facilities could be added to MCL (c.q. **mcx**) to allow for
placing strict domain requirements on matrices when submitted to binary
operations such as addition and multiplication.

## Specifying matrices

From here on, all statements about matrices and graphs are really statements
about matrices and graphs *in the MCL setting*. The *specification*
of a matrix quite closely matches the internal representation.

A matrix M has two domains: the column domain and the row domain. Both
simply take the form of a set (represented as an ordered list) of indices. A
*canonical domain* is a domain of some size K where the indices are
simply the first K nonnegative integers 0,1..,K-1. The domains
dictate which nonzero entries are allowed to occur in a matrix; only entries
M[i,j] are allowed where i is in the row domain and j is in the column
domain.

The matrix M is specified in three parts, for which the second is optional.
The parts are:

Header specification

This specifies the dimensions K and L of the matrix, where K is the
size of the row domain, and L is the size of the column domain.
It looks as follows:

```
(mclheader
mcltype matrix
dimensions 9x14
)
```

This dictates that a matrix will be specified for which the row
domain has dimension 9 and the column domain has dimension 14.

Domain specification

The domain specification can have various forms: if nothing is specified,
the matrix will have canonical domains and a canonical representation,
similar to the representation encountered in textbooks. Alternatively, the
row and column domains can each be specified separately, and it is also
possible to specify only one of them; the other will simply be a canonical
domain again. Finally, it is possible to declare the two domains identical
and specify them simultaneously. It is perfectly legal in each case to
explicitly specify a canonical domain. It is *required* in each case
that the number of indices listed in a domain corresponds with the dimension
given in the header.

An example where both a row domain and a column domain are specified:

```
(mclrows
 100 200 300 400 500 600 700 800 900 $
)
(mclcols
 30 32 34 36 38 40 42 44 46 48 50 52 56 58 $
)
```

This example combines with the header given above, as the dimensions fit.
Had the row domain specification been omitted, the row domain would
automatically be set to the integers 0,1,..8. Had the column
specification been omitted, it would be set to 0,1,..13.

Suppose now that the header did specify the dimensions 10x10.
Because the dimensions are identical, this raises the possibility
that the domains be identical.
A valid way to specify the row domain and column domain in one go is this.

```
(mcldoms
 11 22 33 44 55 66 77 88 99 100 $
)
```

Matrix specification

The matrix specification starts with the sequence

```
(mclmatrix
begin
```

The 'begin' keyword in the '(mclmatrix' part is followed by a list of
listings, where the primary list ranges over all column indices in M (i.e.
indices in the column domain), and where each secondary lists encodes all
positive entries in the corresponding column. A secondary list (or matrix
column) starts with the index c of the column, and then contains a listing
of all row entries in c (these are matrix entries M[r,c] for varying r). The
entry M[r,c] is specified either as 'r' or as 'r:f', where f is a float. In
the first case, the entry M[r,c] defaults to 1.0, in the second case, it is
set to f. The secondary list is closed with the `$' character. A full
fledged examples thus looks as follows:

```
(mclheader
mcltype matrix
dimensions 12x3
)
(mclrows
 11 22 33 44 55 66 77 88 99 123 456 2147483647 $
)
(mclcols
  0  1  2 $
)
(mclmatrix
begin
0    44 88 99 456 2147483647 $
1    11 66 77 123 $
2    22 33 55 $
)
```

Note that the column domain is canonical; its specifiation could have been
omitted. In this example, no values were specified. See below for more.

## Specifying graphs

A graph is simply a matrix where the row domain is the same as the column
domain. Graphs should have positive entries only. Example:

```
(mclheader
mcltype matrix
dimensions 12x12
)
(mcldoms
11 22 33 44 55 66 77 88 99 123 456 2147483647 $
)
(mclmatrix
begin
11    22:2  66:3.4  77:3  123:8 $
22    11:2  33:3.8  55:8.1 $
33    22:3.8  44:7  55:6.2 $
44    33:7  88:5.7  99:7.0 456:3 $
55    22:8.1  33:6.2  77:2.9  88:3.0 $
66    11:3.4  123:5.1 $
77    11:3  55:2.9  123:1.5 $
88    44:5.7  55:3.0  99:3.0 456:4.2 $
99    44:7.0  88:3.0 456:1.8 2147483647:3.9 $
123   11:8  66:5.1  77:1.5 $
456   44:3  88:4.2  99:1.8 2147483647:6.3 $
2147483647   99:3.9 456:6.3 $
)
```

Incidentally, clustering this graph with mcl, using default parameters,
yields a cluster that is represented by the 12x3 matrix shown earlier.

The following example shows the same graph, now represented on a
canonical domain, and with all values implicitly set to 1.0:

```
(mclheader
mcltype matrix
dimensions 12x12
)
(mclmatrix
begin
0    1  5  6  9 $
1    0  2  4 $
2    1  3  4 $
3    2  7  8 10 $
4    1  2  6  7 $
5    0  9 $
6    0  4  9 $
7    3  4  8 10 $
8    3  7 10 11 $
9    0  5  6 $
10   3  7  8 11 $
11   8 10 $
)
```

**Additional notes**  
There are few restrictions on the format that one might actually expect.
Vectors and entries may occur in any order and need not be sorted.
Repeated entries and repeated vectors are allowed but are always
discarded while an error message is emitted.

If you want *functionally interesting behaviour* in combining
repeated vectors and repeated entries, have a look at the next section
and at mcxassemble.

Within the vector listing, the '#' is a token that introduces
a comment until the end of line.

## Raw format

A file in *raw format* is simply a listing of vectors without any
sectioning structure. No header specification, no domain specification, and
no matrix introduction syntax is used - these are supplied to the processing
application by other means. The end-of-vector token '$' must still be used,
and the comment token '#' is still valid. mcxassemble
imports a file in raw
format, creates a native matrix from the data therein, and writes the
matrix to (a different) file. It allows customizable behaviour in how to
combine repeated entries and repeated vectors. This is typically used in the
following procedure. A) Do a one-pass-parse on some external cooccurrence
file/format, generate raw data during the parse and write it to file
(without needing to build a huge data structure in memory). B) mcxassemble
takes the raw data and assembles it according to instruction into a native
mcl matrix.

## Tab format / label information

Several mcl programs accept options such as **-tab**, **-tabc**,
**-tabr**, **-use-tab**, **-strict-tab**,
and **-extend-tab**.
The argument to these options is invariably the name of
a so-called *tab file*.
Tab files are used to convert between labels (describing entities
in the data) and indices as used in the mcl matrix format.
In a tab file each line starts with a unique number which presumably
corresponds to an index used in a matrix file.
The rest of the line contains a
descriptive string associated with the number. It is required
that each string is unique, although not all mcl programs enforce
this at the time of writing.
The string may contain spaces.
Lines starting with
# are considered comment and are disregarded.

**Tab domain**  
The ordered set of indices found in the tab file
is called the *tab domain*.

Tab files are almost always employed in conjunction with an mcl matrix file.
mcxdump and clmformat require by
default that the tab domain coincides with the matrix domain (either row or
column or both) to which they will be applied. This can be relaxed for
either by supplying the **--lazy-tab** option.

mcl provides explicit modes for dealing with tab structures by means of
the **-extend-tab**, **-restrict-tab** and
**-strict-tab** options. Refer to the mcl
documentation.

## Label input

Label input is a line based input where two nodes and an optional value are
specified on each line. The nodes should be specified by labels. If the
labels contain spaces they should be separated by tabs (and the value if
present should be separated from the second label by a tab as well). The
parse code will assume tab-separated labels if it sees a tab character in
the input, otherwise it will split the input on any kind of whitespace.
Any line where the first non-whitespace character is the octothorp (#) is
ignored. The following is an example of label input.

```
---8<------8<------8<------8<------8<---
# the cat and the hat example
cat hat  0.2
hat bat  0.16
bat cat  1.0
bat bit  0.125
bit fit  0.25
fit hit  0.5
hit bit  0.16
--->8------>8------>8------>8------>8---
```

mcl can read in label input and cluster it when it is
given the **--abc** option. It can optionally save
the input graph in native format and save the label information
in a tab file with the **-save-graph** and **-save-tab**
options.

Refer to the MCL getting started and
MCL manual examples sections
for more information on how MCL deals with label input.

mcxload is a general purpose program for reading
in label data and other stream formats. It
encodes them in native mcl format and tab files.
It allows intermediate transformations on the values.

## Transformation syntax

mcl, mcxload, mcxsubs, mcxassemble
all accept the same transformation language in their
respective **tf**-type options and mcxsub's **val**
specification.

A statement in this language is simply a comma-separated
list of functions accepting a single numerical value.
The syntax of a function invocation in general is
**func**(*arg*).
The functions **exp**, **log**, **neglog** can
also be given an empty parameter list, indicating that
*e* is taken as the exponent base. In this case,
the invocation looks like **func**(). The following
functions are supported.

lt

Filter out values greater than or equal to arg.

lq

Filter out values greater than arg.

gq

Filter out values less than arg.

gt

Filter out values less than or equal to arg.

ceil

Set everything higher than arg to arg.

floor

Set everything lower than arg to arg.

mul

Multiply by arg.

add

Add arg to it.

power

Raise to power arg.

exp

Raise arg (*e* if omitted) to value.

log

Take log in base arg (*e* if omitted).

neglog

Take minus log in base arg (*e* if omitted).

**NOTE**  
mcl accepts **--abc-log** and **--abc-neg-log** to specify
log transformations. Similarly, mcxload accepts **--stream-log**
and **--stream-neg-log**. The reason is that probabilities are
sometimes encoded below the precision dictated by the IEEE (32 bit) float
specification. This poses a problem as the mcl applications encode values
by default as floats, and the transformation specifications are always
applied to the mcl encoding. The options just mentioned are applied
after a value has been read from an input stream and *before* it
is converted to the native encoding.

## SEE ALSO

mcxassemble,
and mclfamily for an overview of all the documentation
and the utilities in the mcl family.

## AUTHOR

Stijn van Dongen.
