# Supplementary material for: MCL-CAw: a refinement of MCL for detecting yeast complexes from weighted PPI networks by incorporating core-attachment structure
Source: BMC Bioinformatics. 2010 Oct 12;11:504. doi: 10.1186/1471-2105-11-504 (PMC2965181; doi:10.1186/1471-2105-11-504)
Supplement: Additional files 2 — The MCL-CAw software package: The source code and installation details for the MCL-CAw software. [file 1471-2105-11-504-S2.ZIP › Additional_files_2/mcl-09-149.tar.gz!/mcl-09-149/doc/tingea.log.html]

How to set the Tingea log parameters


29 May 2009   
**tingea.log**
1.008, 09-149

## NAME

tingea.log — How to set the Tingea log parameters

## DESCRIPTION

The Tingea logging framework is part of the Tingea library.
In this context logging means that a pgrogram issues statements
about what it is doing. It can do so for different purposes
and at different levels of verbosity. By default logging statements
are written on STDERR.

Tingea logging provides a quick and easy way for programmers to associate
verbosity levels with logging statements.
Only logging statements for which the verbosity
level does not exceed the user-imposed threshold will be executed.
Users can easily regulate the verbosity level by setting the environment
variable TINGEA\_LOG\_TAG.
Alternatively, programs may accept a command line argument.
The format accepted by both environment variable and command line argument
is identical.
For the command line the programmer is free in choosing the option name.
It is customarily named **-q**.
The availability of such an option may vary from program to program.
However, any program that makes use of the Tingea logging facilities
can be regulated with the TINGEA\_LOG\_TAG environment variable.
If a **-q** type option is present and the environment variable
is set, then the environment variable is interpreted first followed
by the **-q** argument.

Tingea logging allows a programmer to assign categories to logging
statements. The categories FUNCTION and DATA have a subdivision
ranging from fine-grained to coarse-grained. The categorie MONITORING has
a subdivision ranging from low priority to high priority.
The other categories are unimodal. These are IO, THREAD, PROCESS,
and GAUGE. Three unspecified unimodal categories are SLOT1, SLOT2,
and SLOT3. They can be used to encode program-specific semantics.

The programmer may assign multiple categories to a single logging statement.
It is unusual for more than two categories to be specified. For example, IO
and DATA at the LIST level may be combined to indicate a logging statement
that provides data summaries for a certain IO related information. In order
of granularity the DATA levels are CELL, LIST, and AGGREGRATE. If the user
accepts IO logging and accepts DATA logging at level CELL or LIST the
statement will be executed. If no IO logging is accepted or DATA logging is
only accepted at the AGGREGRATE level, the statement will be skipped.

By default, all categories that are specified by the programmer need
to pass the threshold specified by the user for that category.
The user may relax this requirement so that only one category needs
to pass the user threshold. In the above example, the statement
categorized as both IO and DATA at LIST level will be accepted
if the user specifies IO and DATA at AGGR level with OR semantics.

## SYNTAX

The syntax of the TINGEA\_LOG\_TAG environment variable is described by

```
   [[189x]]{<[dfgimpstABC][1-9]>*,[V]}
```

which translates to the following. An optional lead tag is followed
by a concatenation of units. A unit is either a pair in
[dfgimpstABC] x [1-9x] or the single character V.
The leading tag semantics are described further below.
The single character V, if present, specifies that OR semantics
should be used rather than the default AND semantics. The semantics
for the other units are given below.

```
   d     DATA
            1  CELL
            2  LIST
            3  AGGR
            x  turned off

   f     FUNCTION
            1  LINE
            2  FUNCTION
            3  MODULE
            4  APPLICATION
            x  turned off

   m     MONITORING
            1  DEBUG
            2  INFO
            3  WARNING
            4  ERROR
            5  PANIC
            x  turned off

   g     GAUGE    |
   i     IO       |
   n     NETWORK  |
   p     PROCESS  |  (Inter Process really)
   t     THREAD   |______  1 on
                  |        x off
   A     SLOT1    |
   B     SLOT2    |
   C     SLOT3    |
```

The leading tag can be used to set levels for all categories at once.
Subsequent units may then alter this intial setting.
The lead tag settings and their meaning are these:

```
   1     d1f1m1g1i1p1s1t1A1B1C1     # very yappy
   9     d3f4m5gxixpxsxtxAxBxCx     # very terse, only d f m
   8     d3f4m5g1i1p1s1t1A1B1C1     # less terse
   x     dxfxmxgxixpxsxtxAxBxCx     # silent
```

All categories accept values between 1 and 9 in addition to the
value x. As seen above, only a few categories contain more than
one level and no category contains more than five levels.
The rule is that if a level exceeds the maximul level available for a category
it is simply interpreted as the maximum level.

The GAUGE category, if set, indicates that a program may write line based
progress bars or other output in which a single line is accumulated over
multiple statements. This implies that a single GAUGE logging statement may
not result in newline-terminated output. This is undesirable in case the
logging stream is directed to a file that is written to by other
applications as well. In that case, turn off GAUGE. All other categories are
garantueed to result in line-terminated output, by virtue of the programmer
contract.

## AUTHOR

Stijn van Dongen.
